# Abstracts from Hydrocephalus 2019: The Eleventh Meeting of the International Society for Hydrocephalus and Cerebrospinal Fluid Disorders

**DOI:** 10.1186/s12987-019-0156-3

**Published:** 2019-12-05

**I1**

Thomas J. Zwimpfer MD, PhD, President, Hydrocephalus 2019

Department of Surgery, University of British Columbia, Vancouver, Canada

**Correspondence:** Thomas J. Zwimpfer - thomas.zwimpfer@ubc.ca

It has been a great pleasure to host the **11th Meeting of the Hydrocephalus Society (International Society for Hydrocephalus and Cerebrospinal Fluid Disorders) in Vancouver, British Columbia, Canada from September 13 to 16, 2019.** This year we hosted 216 individuals from 19 countries. This annual meeting brought together individuals with a wide variety of backgrounds, including clinicians and basic scientists, varying from professors to trainees, as well as nurses, nurse-practitioners, therapists, patient advocates and volunteers. They all shared common interests and goals: to better understand the normal physiology of cerebrospinal fluid and intracranial pressure, to improve the diagnosis and treatment of hydrocephalus and other CSF disorders in both children and adults.

The Pre-Meeting Educational Day focused on broad topics such as: (1) Physiology of CSF/ICP and Pathophysiology of Hydrocephalus; (2) Experimental Hydrocephalus; (3) Pediatric Hydrocephalus and; (4) Chronic Hydrocephalus, a cause of reversible cognitive impairment.

Highlights of the Meeting included;

- 36 high quality submissions to the Young Investigator Award competition.
- Two Hakim lectures: (1) Interactions between iNPH and AD and; (2) Factors related to Ventriculomegaly and its relationship to Aging.
- The Marmarou Lecture: The Umea Experience in CSF Dynamics in Hydrocephalus,
- Presentation by the New International iNPH Guidelines Working group.
- Keynote lectures on: (1) Results of the UK Basics trial on antibiotic-impregnated shunt catheters; (2) Basic science research on shunt blockage; (3) Research on shunt outcomes; (4) Diffusion Tensor Imaging in Pediatric Hydrocephalus; (5) Artificial Intelligence in Hydrocephalus Research and; (6) Update on Models of Experimental Hydrocephalus.
- Panel Presentation on the various International Hydrocephalus Registries.
- International Hydrocephalus Imaging Working Group Presentations.

The highlight of the Social activities was a cable car trip to the top of Grouse Mountain for fine city views and dining, to experience a Lumberjack Show and a very close-up visit with two 1000 lb Grizzly bears, Grinder and Coola, who have called Grouse Mountain home for the past 18 years after being orphaned as cubs.

I look forward to seeing many of you in Gothenburg, Sweden in September 2020 for the 12th Meeting of the Hydrocephalus Society.

I wish you a productive and exciting year.

Tom Zwimpfer

## O1 Prevalence and clinical associations of disproportionately enlarged subarachnoid space hydrocephalus, an imaging feature of idiopathic normal pressure hydrocephalus

### Chihiro Akiba^1^, Bibek Gyanwali^2^, Steven Villaraza^2^, Madoka Nakajima^1^, Masakazu Miyajima^1^, Narayanaswamy Venketasubramanian^2^, Saima Hilal^2^, Christopher Chen^2^

#### ^1^Department of Neurosurgery, Juntendo University, Tokyo, Japan; ^2^Memory Aging and Cognition Centre, National University of Singapore, Singapore

##### **Correspondence:** Chihiro Akiba - chihi-rocket@hotmail.co.jp

*Fluids and Barriers of the CNS* 2019, **16(Suppl 3):**O1

**Introduction:** Disproportionately Enlarged Subarachnoid space Hydrocephalus (DESH) is a major imaging feature of idiopathic normal pressure hydrocephalus (iNPH). High incidence of iNPH has been reported in subjects with DESH. We aimed to investigate prevalence of DESH in Singaporean cohorts and to assume prevalence of iNPH.

**Methods:** A cross-sectional case–control design was used. Subjects aged 60 and over in a memory clinic and a community-based cohort were assessed for the presence of DESH, i.e. ventriculomegaly, Sylvian dilatation, and high convexity tightness and a clinical triad of iNPH, i.e. cognitive, gait and urinary symptoms. We defined subjects that fulfilled all of DESH components as DESH-group, subjects with ventriculomegaly but lacking some of other DESH components as Ventriculo-group, subjects with Sylvian dilatation but lacking some of other DESH components as Sylvian-group, and subjects with high convexity tightness but lacking some of other DESH components as Convexity-group.

**Results:** The prevalence of DESH was 1.1% in the memory clinic (548 subjects) and 1.0% in the community (946 subjects), which increased with age. The clinical triad was significantly more frequent in DESH-group (memory clinic/community: 50%/11%), compared to those with normal images (none/none), Sylvian-group (7%/2%), and Ventriculo-group (12%/7%). In the triad, gait disturbance was significantly more frequent in DESH-group (83%/33%) compared to those with normal images (2%/1%), Sylvian-group (14%/4%), and ventriculo-group (26%/10%).

**Conclusions:** The prevalence of DESH was around 1% in Singaporean cohorts. High convexity tightness was specifically associated with the clinical triad of iNPH. Gait disturbance seemed to be the most specific to DESH and high convexity tightness.

## O2 The association between the MMSE score improvement after tap test and that after shunt surgery in idiopathic normal pressure hydrocephalus

### Yuki Asahara^1^, Shinji Miyagawa^1^, Masamichi Atsuchi^2^, Hiroyasu Nagashima^2^, Kazushige Kobayashi^3^, Masahiko Suzuki^1^

#### ^1^Department of Neurology, The Jikei University Katsushika Medical Center, Tokyo, Japan; ^2^Department of Neurosurgery, The Jikei University Katsushika Medical Center, Tokyo, Japan; ^3^Department of Rehabilitation, The Jikei University Katsushika Medical Center, Tokyo, Japan

##### **Correspondence:** Yuki Asahara - yuki.asahara.1988@gmail.com

*Fluids and Barriers of the CNS* 2019, **16(Suppl 3):**O2

**Introduction:** Idiopathic normal pressure hydrocephalus (iNPH) causes dementia as well as gait disturbance. Tap test can predict the gait improvement after shunt surgery, but the cognitive improvement after the operation is obscure. The objective of this study was to evaluate the association between the cognitive function recovery after tap test and that after shunt surgery.

**Methods:** We retrospectively evaluated 48 iNPH patients who underwent shunt surgery from January 2016 to July 2018. Their mean age was 78 years old. 43 and 5 cases underwent LP and VA shunt surgery respectively. We assessed whether the pre- and post-tap test difference of Mini-Mental State Examination (MMSE) score was associated with the pre-tap test and post-shunt surgery difference by linear regression analysis. Multivariate logistic regression analysis was also performed to reveal the associations between clinical parameters and the MMSE score improvement after shunt surgery. The study was approved by the ethics committee for medical research at our hospital.

**Results:** The pre- and post-tap test MMSE score difference was significantly associated with the pre-tap test and post-shunt surgery difference (p = 0.005). The correlation coefficient was 0.396. On multivariate logistic regression analysis, the MMSE score improvement after the operation was associated with the score improvement after tap test (p = 0.026) and serum total cholesterol (p = 0.023).

**Conclusions:** In our study, the MMSE score improvement after tap test was significantly associated with that after shunt surgery in iNPH.

## O3 Ovine PIA arachnoid complex: biomechanical characterization

### Gabryel Conley Natividad^1^, Sophia K. Theodossiou^1^, Nathan R. Schiele^1^, Gordon Murdoch^2^, Goutham Burla^1^, Gabriel Potirniche^3^, Martin Mortazavi^4,5^, Anastasia Vechera^5^, Farzad H. Adl^5^, Bryn A. Martin^1^

#### ^1^Department of Biological Engineering, University of Idaho, Moscow, USA; ^2^Department of Animal and Veterinary Science, University of Idaho, Moscow, USA; ^3^Department of Mechanical Engineering, University of Idaho, Moscow, USA; ^4^California Institute of Neuroscience, Thousand Oaks, California, USA; ^5^National Skull Base Foundation, Thousand Oaks, California, USA

##### **Correspondence:** Bryn A. Martin - brynm@uidaho.edu

*Fluids and Barriers of the CNS* 2019, **16(Suppl 3):**O3

**Introduction:** The pia arachnoid complex (PAC) is a cerebrospinal fluid-filled tissue that surrounds the brain and spinal cord. Within the PAC, arachnoid trabeculae (AT) fibers, AT sheets, and blood vessels span the space between the arachnoid and pia surfaces. Due to its structural role, alterations to the biomechanical properties of the PAC caused by sub-concussive hits could impact traumatic brain injury (TBI). The aim of this study was to quantify the mechanical and structural properties of ovine PAC.

**Methods:** Ovine brain samples (n = 10) were harvested and removed from the skull within 30 min post-mortem. To access the brain tissue, skulls were split medially from the occipital region to down to the nose on the superior and inferior aspects of the skull. A template was used to remove brain samples from different regions of the brain. At < 2-h PAC samples were tested with uniaxial tension at ~ 2 mm/s until failure. The force and displacement data were acquired at 100 Hz using LabVIEW and tissue structure was characterized using confocal microscopy.

**Results:** An average strain rate of 0.59 s^−1^ was observed for PAC samples subjected to uniaxial tension. Using a Mooney-Rivlin model for average stress-strain curve fit, Young’s modulus of the linear region was found to be 7.68 ± 3.0 MPa. The mean ultimate stress and strain were found to be 2.69 ± 0.76 MPa and 0.60 ± 0.13, respectively. There were no significant differences across sample locations.

**Conclusions:** To our knowledge, this preliminary study represents the first biomechanical characterization of ovine PAC.

## O4 CSF dynamics following endoscopic repair of anterior skull base defects

### Claudia L. Craven, Sophie R. Mullins, Hasan Asif, Linda D’Antona, Simon Thompson, Laurence D. Watkins, Ahmed K. Toma

#### Victor Horsley Department of Neurosurgery, National Hospital for Neurology and Neurosurgery, London, WC1N 3BG, UK

##### **Correspondence:** Claudia Craven - claudia.craven@gmail.com

*Fluids and Barriers of the CNS* 2019, **16(Suppl 3):**O4

**Introduction:** High intracranial pressure (ICP) states can be the major driving force behind an anterior CSF leak. Cerebrospinal fluid (CSF) diversion prior to endoscopic repair of a skull base defect is a therefore common. Our centre utilizes shunt reservoirs that non-invasively record ICP. We present our experience of the Sensor Reservoir^®^ (Meithke) through three patient cases and report the observed changes in CSF dynamics before and after repair.

**Methods:** Prospective case series of patients with a Sensor Reservoirs^®^ in situ who underwent endoscopic repair of an anterior skull base leak. ICP and pulse amplitude (PA) were non-invasively recorded in 2 postural positions (lying flat and sitting up) on day − 1, day + 1 and week + 2 after endoscopic repair.

**Results:**4 Female and 1 male patient, mean age (± SD) 43.4 years, underwent VP shunt insertion with Sensor Reservoirs^®^ in situ. Day one prior to endoscopic repair, mean ICP was − 0.2 mmHg. Day one post-endoscopic repair, ICP and PA had increased by a mean of 2 mmHg. After 2 weeks the CSF dynamics had returned to pre-endoscopic repair level. Postural differences in ICP and PA in sitting and lying were significantly reduced following endoscopic repair (p = 0.003). At the point of follow-up there were no recurrences in the CSF leaks.

**Conclusions:** Sensor Reservoirs^®^ enabled non-invasive confirmation that ICP was sufficiently reduced to prevent failure of the leak repair. Furthermore, we observed both a novel alteration in CSF dynamics following endoscopic repair of an anterior skull base CSF leak, in which postural fluctuations in ICP are reduced.

## O5 Absence of spontaneous retinal venous pulsation can be seen with severe shunt overdrainage

### Linda D’Antona^1^, James A. McHugh^2^, Claudia L. Craven^1^, Simon D. Thompson^1^, Lewis Thorne^1^, Manjit S. Matharu^3^, Laurence D. Watkins^1^, Fion Bremner^2^, Ahmed K. Toma^1^

#### ^1^Victor Horsley Department of Neurosurgery, The National Hospital for Neurology and Neurosurgery, London, UK; ^2^Department of Neuro-Ophthalmology, The National Hospital for Neurology and Neurosurgery, London, UK; ^3^Headache and Facial Pain Group, UCL Queen Square Institute of Neurology, London, UK

##### **Correspondence:** Linda D’Antona - linda.d’antona@nhs.net

*Fluids and Barriers of the CNS* 2019, **16(Suppl 3):**O5

**Introduction:** Spontaneous retinal venous pulsations (SVP) are a complex physiological phenomenon related to the balance between the pulse pressures in the intraocular and intracranial compartments. The absence of SVP is considered a sign of raised intracranial pressure (ICP) but the absence of SVP in patients with low ICP has never been described before. In this case series we report 2 cases of patients with severe shunt overdrainage and absence of SVP.

**Methods:** Single centre prospective observational study. Patients admitted for elective intraparenchymal ICP monitoring between December 2017 and April 2019 underwent ophthalmology exam during the monitoring period. The exam included infrared OCT video recordings of the retina and assessment of the SVP by two neuro-ophthalmologists both masked to the ICP results.

**Results:** Among a group of 111 patients assessed with ICP monitoring for various clinical indications, 7 patients had shunt overdrainage (median ICP over 24 h < − 1 mmHg) and required valve adjustment, shunt revision or shunt ligation. Two patients (2F, age 31 ± 18 years) with severe shunt overdrainage had absence of SVP bilaterally according to both examiners, no papilloedema and normal intraocular pressure. Their median ICP over 24 h was particularly low (− 5.33 mmHg and − 2.98 mmHg respectively).

**Conclusions:** Two out of 7 patients with shunt overdrainage had bilateral absence of SVP. Extremely low ICP could be associated with the absence of SVP on ophthalmic exam due to the imbalance between the ICP and intraocular pulse pressures. Larger studies could confirm this finding and support the absence of SVP as a potential marker of severe shunt overdrainage.

## O6 Can we reduce the duration of intracranial pressure monitoring?

### Linda D’Antona^1^, Claudia Craven^1^, Joana Ramos^1^, Simon Thompson^1^, Joseph Davies^1^, Manjit S. Matharu^2^, Lewis Thorne^1^, Laurence D. Watkins^1^, Ahmed K. Toma^1^

#### ^1^Victor Horsley Department of Neurosurgery, The National Hospital for Neurology and Neurosurgery, London, UK; ^2^Headache and Facial Pain Group, UCL Queen Square Institute of Neurology, London, UK

##### **Correspondence:** Linda D’Antona - linda.d’antona@nhs.net

*Fluids and Barriers of the CNS* 2019, **16(Suppl 3):**O6

**Introduction:** Elective intraparenchymal intracranial pressure (ICP) monitoring is commonly performed for periods of 24–48 h. This study investigates the value of a short standardised ICP assessment in predicting the final diagnosis of patients undergoing intraparenchymal ICP monitoring.

**Methods:** Single centre prospective study. Patients admitted for elective 24 h ICP monitoring between January 2018 and March 2019 who underwent a standardised exercise battery were included. The exercise battery included 4 positions (2 min each): supine, lumbar puncture (LP) position (in lateral decubitus), sitting and standing. Mean ICP and pulse amplitude were obtained for each position. The diagnosis of “High ICP” was based on the clinical presentation, imaging and 24 h ICP monitoring results. ROC and area under the curve (AUC) analysis were performed to investigate the accuracy of the short exercise battery in predicting the final diagnosis of “High ICP”.

**Results:** Eighty patients (59F, mean age 40 ± 14 years) were included. The final diagnostic groups were as follow: 30 high ICP, 28 normal ICP, 16 low ICP, 6 abnormal brain compliance. The statistical analysis showed that a 2-min supine mean ICP above 10.42 mmHg predicts the final diagnosis of “High ICP” with an accuracy of 91.3%, sensitivity and specificity of 90% and 92% respectively (AUC 0.84). The predictive value of the standardised battery for the other final diagnostic groups was also investigated.

**Conclusions:** Short standardised ICP assessments can effectively predict the results of 24 h ICP monitoring. These results support the possibility of reducing the duration ICP monitoring and patients’ length of stay.

## O7 Prospective study on the effects of position on intracranial compliance

### Linda D’Antona^1^, Claudia Craven^1^, Simon Thompson^1^, Joana Ramos^1^, Jonathan Funnell^1^, Saniya Mediratta^1^, Manjit S. Matharu^2^, Lewis Thorne^1^, Laurence D. Watkins^1^, Ahmed K. Toma^1^

#### ^1^Victor Horsley Department of Neurosurgery, The National Hospital for Neurology and Neurosurgery, London, UK; ^2^Headache and Facial Pain Group, UCL Queen Square Institute of Neurology, London, UK

##### **Correspondence:** Linda D’Antona - linda.d’antona@nhs.net

*Fluids and Barriers of the CNS* 2019, **16(Suppl 3):**O7

**Introduction:** Intracranial pressure (ICP) changes dynamically depending on the body posture. This study investigates the effect of different body positions on brain compliance testing the correlation between pulse amplitude and body position in patients undergoing intraparenchymal ICP monitoring.

**Methods:** Single centre prospective study. Patients admitted for elective 24-h ICP monitoring between January 2018 and March 2019 who underwent a standardised exercise battery were included. The exercise battery included 4 positions (2 min each): supine, lumbar puncture (LP) position (in lateral decubitus), sitting and standing. Mean pulse amplitude for each position was calculated using the software ICM Plus^®^ (University of Cambridge, UK). The effect of position on pulse amplitude was assessed through a linear regression model including the following covariates: age, gender, shunt presence and final diagnosis.

**Results:** Twenty-nine patients (19F, mean age 39 ± 12 years) having a total of 116 pulse amplitude measurements were included. The indications for ICP monitoring were heterogeneous and 12 patients had a shunt in place at the time of monitoring. Their 24-h median ICP was 7.9 ± 8.3 mmHg and the median pulse amplitude was 6.1 ± 3.3 mmHg. The linear regression model did not show any association between pulse amplitude and body position (p = .93, 95% CI − 0.5 to 0.49) but demonstrated that patients with a shunt have a lower pulse amplitude compared to patients without a shunt (p = .003, 95% CI − 3.57 to − .77).

**Conclusions:** These results suggest that brain compliance is not significantly affected by body position.

## O8 The effect of sedation on intracranial opening pressure

### Linda D’Antona^1^, Claudia L. Craven^1^, Abhiney Jain^1^, Simon D. Thompson^1^, Manjit S. Matharu^2^, Lewis Thorne^1^, Astri M. V. Luoma^3^, Ahmed K. Toma^1^, Laurence D. Watkins^1^

#### ^1^Victor Horsley Department of Neurosurgery, The National Hospital for Neurology and Neurosurgery, University College London Hospital, UK; ^2^Headache and Facial Pain Group, UCL Queen Square Institute of Neurology, London, UK; ^3^Department of Anaesthesia and Intensive Care, The National Hospital for Neurology and Neurosurgery, University College London Hospital, UK

##### **Correspondence:** Linda D’Antona - linda.d’antona@nhs.net

*Fluids and Barriers of the CNS* 2019, **16(Suppl 3):**O8

**Introduction:** Sedation is often employed in order to improve the experience of patients who undergo lumbar punctures. The effect of sedation on lumbar puncture opening pressures is not entirely known. In this study we investigated the effect of sedation on intracranial opening pressure in a cohort of patients who underwent continuous intraparenchymal ICP monitoring.

**Methods:** Observational study. Intraoperative intracranial opening pressures of patients undergoing ICP monitoring under sedation was compared to median 24 h ICP results and mean ICP obtained in different body positions (including lumbar puncture position). In a subset of patients, the level of sedation and CO_2_ were also monitored continuously.

**Results:** Forty-two patients (32 F, age 42 ± 14 years) were included. Fifteen patients had a shunt. The indications for ICP monitoring were diverse: 16 diagnostic, 12 hydrocephalus, 8 Chiari malformation, 6 IIH.

Patients’ median 24-h ICP, night ICP and lumbar puncture position ICP were significantly lower when compared to the intracranial opening pressure first noted via the bolt (paired t-tests: p < 0.01 95% CI 13.8 to 19.8, p < 0.01 95% CI 10.9 to 17.2 and p < 0.01 95% CI 4.4 to 11.9 respectively).

The average difference between opening pressure and median 24 h ICP was 17 ± 10 mmHg and ranged from 6 to 59 mmHg.

**Conclusions:** In this cohort of patients, sedation caused a significant increase in intracranial opening pressure and made this measurement an unreliable estimate of the ICP. Larger studies will be needed to investigate the effect of specific sedative agents on ICP.

## O9 Evaluation of Alzheimer’s disease related CSF biomarkers in idiopathic normal pressure hydrocephalus

### Jacqueline A. Darrow, Alexandria E. Lewis, Kristina Khingelova, Seema Gulyani, Abhay R. Moghekar

#### Department of Neurology, Johns Hopkins University, Baltimore, MD, 21287, USA

##### **Correspondence:** Abhay Moghekar - am@jhmi.edu

*Fluids and Barriers of the CNS* 2019, **16(Suppl 3):**O9

**Introduction:** The role of biomarkers in the selection of idiopathic normal pressure hydrocephalus (iNPH) patients for shunt surgery has been studied in small populations. Our aim was to evaluate potential differences in canonical Alzheimer’s disease related biomarkers [abeta-42 (aβ42), aβ40, tau (t-tau), and phosphorylated-tau (p-tau)] between patients selected for shunt surgery based on their response to a CSF diversion procedure and to determine if they correlate with age, cognition, and gait in a large iNPH population (n = 220).

**Methods:** CSF was obtained from iNPH patients after a baseline assessment including Montreal Cognitive Assessment (MOCA), Timed Up and Go (TUG), and 10 M walk testing prior to their procedure. Patients were deemed responders and referred for shunt surgery based on iNPH guidelines. CSF was analyzed on the electrochemiluminescent Lumipulse G1200 (Fujirebio) platform.

**Results:** In this iNPH cohort, the mean age was 73 ± 10 years and there were 140 males and 80 females. There was no correlation between these biomarkers and cognition and gait measurers in our population.

**Conclusions**: In this iNPH population, we did not observe differences in AD biomarkers between CSF diversion responders and non-responders; therefore, they should not be used to screen patients for shunt surgery. Future studies will be necessary to determine if these biomarkers predict long-term shunt response.

## O10 Bolt external ventricular drains—a paradigm shift in the management of acute hydrocephalus?

### Debayan Dasgupta^1^, Jonathan Funnell^2^, Lena K. Pfeffer^2^, Selma Al-Ahmad^1^, Ugan Reddy^3^, Lewis Thorne^1^, Carmel Curtis^4^, Laurence D. Watkins^1^, Ahmed Toma^1^

#### ^1^Victor Horsley Department of Neurosurgery, National Hospital for Neurology and Neurosurgery, University College London Hospitals NHS Foundation Trust, UK; ^2^University College London Medical School, UK; ^3^Department of Neuroanaesthesia and Neurocritical Care, National Hospital for Neurology and Neurosurgery, University College London Hospitals NHS Foundation Trust, UK; ^4^Department of Clinical Microbiology, University College London Hospitals NHS Foundation Trust, UK

##### **Correspondence:** Debayan Dasgupta - debayan.dasgupta1@nhs.net

*Fluids and Barriers of the CNS* 2019, **16(Suppl 3):**O10

**Introduction:** The management of acute hydrocephalus is a constantly developing and improving area within neurosurgery. We recently demonstrated a standardised perioperative care bundle and simulation training improves placement and infection rates of tunnelled external ventricular drains (EVDs)—we have since developed our practice further to include minimally-invasive bolt EVDs. These are inserted with a smaller calibre hand drill, and can be inserted in an ITU environment. This study compares the infection rates, placement accuracy, and time from decision to CSF access between the modalities. Furthermore, we have begun to introduce the use of the LiquoGuard drainage system instead of the standard gravitational Becker drain, particularly in cases with significant intraventricular blood, or where lead clinicians feel there is a high risk of drain blockage.

**Methods:** A combined retrospective and prospective cohort study of every EVD at our quarternary referral neurosurgical centre, 1/12/18–30/4/19.

**Results:** In the 5 months, 46 EVDs were inserted—23 of each type. Of these, 7 were connected to a LiquoGuard. Preliminary data demonstrates average time to CSF access was 140 min for bolt EVDs, and 337 min for tunnelled (p = 0.0015). Accuracy of placement was comparable between the modalities at approximately 90%. There were no infections noted. Average length of drainage was 9.7 days in bolt EVDs, and 8 days in tunnelled.

**Conclusions:** Our data demonstrates that bolt EVDs provide a statistically significant faster time to access of the CSF, have a comparably low infection rate, and are accurately placed in trained hands as often as tunnelled EVDs.

## O11 Non-human algorithmic AI agents for systematic online ct image classification and the recognition of hydrocephalus

### Joseph Davids^1^, Susruta Manivannan^2^, Mohammed Elborady^2^, Hadie Adams^2^, Firat Aktalay^2^, James Samarasekara^1^, William Dawes^1^, Claudia Craven^1^, Linda D’Antona^1^, Lewis Thorne^1^, Ahmed Toma^1^, Laurence D. Watkins^1^

#### ^1^Department of Neurosurgery, University College London Hospitals, Queen Square, UK; ^2^Department of Neurosurgery, Cardiff University Hospitals, Wales, UK

##### **Correspondence:** Joseph Davids - josephdarlington.davids@nhs.net

*Fluids and Barriers of the CNS* 2019, **16(Suppl 3):**O11

**Introduction:** CT imaging remains the most common initial diagnostic modality for hydrocephalus in the United Kingdom. We explored whether the diagnosis of hydrocephalus can be achieved by non-human diagnostic agents. Several machine learning models exist for image classification, but their adoption in clinical practice for hydrocephalus in the UK remains underutilised. This study attempted to use a machine learning framework engine to classify CT images of hydrocephalus patients.

**Methods**: A systematic image literature search through Google scholar and Google image analysis was performed to acquire suitable public domain images, while avoiding the geographic impracticalities and differences that surround patient consent and the General Data Protection Regulations for clinical image data. Selected training and test images were verified by specialist neurosurgeons, who also reviewed normal control brain images. A hierarchical supervised learning algorithm was implemented for axial static radiographs at lateral ventricular, third ventricular and cisternal level. The model was evaluated by introducing newer images, which it had not been presented with before.

**Results:** 77.78% accuracy of recognition of hydrocephalus was achieved through training the algorithm on the small number of CT images identified in the search online (N = 20 hydrocephalus and N = 12-control images).

**Conclusions:** Non-human diagnostic agents can achieve relatively good predictive accuracy for hydrocephalus with very little training data. Further training with more images to improve the accuracy and sensitivity of detection is currently underway.

## O12 Sub-10-minute algorithmic demonstration of an artificial intelligence framework engine for ct image feature extraction and recognition of hydrocephalus—a mobile app hydrocephalus predictor

### Joseph Davids^1^, Pranoy Das^1^, Mohammed Elborady^1^, Hadie Adams^2^, James Samarasekara^1^, William Dawes^1^, Claudia Craven^1^, Linda D’Antona^1^, Lewis Thorne^1^, Ahmed Toma^1^, Laurence Watkins^1^

#### ^1^Department of Neurosurgery, University College London Hospitals, Queen Square, UK; ^2^Department of Neurosurgery, Imperial College London, UK

##### **Correspondence:** Joseph Davids - josephdarlington.davids@nhs.net

*Fluids and Barriers of the CNS* 2019, **16(Suppl 3):**O12

**Introduction:** Doctors in the United Kingdom are being encouraged by the Health Secretary to design and utilize mobile and desktop apps, to enable diagnostic and therapeutic efficiency. Machine learning is considered a cost-effective option that could cater for such high throughput of patients with neurological diseases in emergency radiological clinical settings. Currently high costs and complexity of infrastructure for image analysis limits widespread adoption. We live-demo a framework implementation on neurosurgical CT Head and MRI images in patients with suspected hydrocephalus.

**Methods**: We show that it can be used to classify images of patients with hydrocephalus, compared to controls. Preliminary training was performed on a laptop. Subsequently, trained models were used to build a basic mobile application that allowed a clinician to identify hydrocephalus on a new CT image not yet introduced to the model. A > 75% threshold for classification precision was deemed an acceptable accuracy marker warranting further improvement by using greater numbers of training scans.

**Results:** We demonstrate accuracy of > 75% on a small image sample space (N = 32) and show that it is efficient and effective at enabling early low barrier entry into development of artificially intelligent applications for hydrocephalus.

**Conclusions:** Democratisation of Artificial Intelligence is possible and can be achieved cheaply with computer vision technologies from companies like NVIDIA(Clara), Apple(CoreML), Google(TensorFlow), Intel(Movidius). We aimed to leverage these technologies in the design of a fast algorithm for the prediction of CT and MRI images, and demonstrate good preliminary precision on CT images.

## O13 Model for predicting the outcome of diagnostic lumbar punctures for patients presenting with idiopathic normal pressure hydrocephalus

### Alexander J. Davis^1^, Abhay Moghekar^1^, Mark Luciano^3^, Sevil Yasar^2^

#### ^1^Department of Neurology, Johns Hopkins University, Baltimore, Maryland, USA; ^2^Johns Hopkins University School of Medicine, Johns Hopkins-Center on Aging and Health, Baltimore, Maryland, USA; ^3^Department of Neurosurgery, Johns Hopkins University, Baltimore, Maryland, USA

##### **Correspondence:** Abhay Moghekar - am@jhmi.edu

*Fluids and Barriers of the CNS* 2019, **16(Suppl 3):**O13

**Introduction:** The literature is sparse regarding which objective and subjective measures are predictive of improvement in gait following a large volume Lumbar Puncture (LP). There is no established model that is both sensitive and specific for predicting gait improvement after an LP. The present study aims to address the lack of information regarding the predictive value of baseline assessments of patients with Idiopathic Normal Pressure Hydrocephalous (iNPH). We hypothesize that we will identify a sensitive and specific model for predicting which patients will have significant improvement in their gait following an LP.

**Methods:** A retrospective chart review of 238 patients that underwent a LP at the Johns Hopkins Cerebral Fluid Center within the departments of Neurology, between June 2013 and March 2019. Multiple Logistic regression was used to create a sensitive and predictive model for predicting the outcome of LPs for patients with suspected iNPH.

**Results:** Our current model based on analysis of baseline gait (TUG, DualTUG, 10 M Walk, 6 Minute distance, Mini-BEST and Biodex balance scales) and cognitive measures showed the following characteristics:

Sensitivity: 63.64%

Specificity: 94.37%

Positive Predictive Value: 77.78%

Negative Predictive Value: 89.33%

Correctly classified: 87.10%

**Conclusions:** We present a model that is both sensitive and specific for predicting the outcome of LPs. The aim of this model is to assist clinicians and researchers in selecting which patients presenting with iNPH should be referred for an LP. The high specificity and negative predictive value should help identify subjects unlikely to improve from CSF drainage and avoid shunt surgery.

## O14 Reliable change indices for patients presenting with idiopathic normal pressure hydrocephalus

### Alexander J. Davis^1^, Sevil Yasar^2^, Mark Luciano^3^, Abhay Moghekar^1^

#### ^1^Department of Neurology, Johns Hopkins University, Baltimore, Maryland, USA; ^2^Johns Hopkins University School of Medicine, Johns Hopkins-Center on Aging and Health, Baltimore, Maryland, USA; ^3^Department of Neurosurgery, Johns Hopkins University, Baltimore, Maryland, USA

##### **Correspondence:** Abhay Moghekar - am@jhmi.edu

*Fluids and Barriers of the CNS* 2019, **16(Suppl 3):**O14

**Introduction:** Presently, there are no established reliable change indices (RCI) for use in the idiopathic Normal Pressure Hydrocephalous (iNPH) population. The present study aims to establish the first RCIs for patients presenting with iNPH. We calculated RCIs for the following core measures: Montreal Cognitive Assessment (MoCA), Symbol Digit Modalities Test (SDMT), Timed Up & Go (TUG), Dual Timed UP & Go (Dual TUG), 10 Meter Walk Test (10 MWT), Mini-Balance Evaluation Systems test (Mini-BESTest), Modified clinical test of sensory interactions and balance (mCTSIB) and 6-Minute Walk Test (6 MWT).

**Methods:** A retrospective chart review of 382 patients at the Johns Hopkins Cerebral Fluid Center within the departments of Neurosurgery and Neurology, between June 2013 and March 2019. Hierarchal Linear modeling and McSweeney regression based RCIs were calculated.

**Results:** The following is a table representing the Pre-LP vs Post LP mean percent change which is accounted for the inherent variability in the measurement of individual variables.

**Table:** 

Measure	n	Proportion of Pre-LP vs Post-LP test mean (% change)	95% CI		P-value
MoCA	157	3.7%	1.4%	5.9%	0.002
SDMT	148	2.4%	0.7%	5.4%	0.135
TUG	217	− 18.2%	− 23.5%	− 12.8%	0.001
Dual TUG	207	− 18.3%	− 23.3%	− 13.3%	0.001
TenMWT	211	− 21.1%	− 26.1%	− 16.1%	0.001
Mini-BESTest	208	17.3%	15.1%	19.5%	0.001
6 MWT	214	16.2%	13.5%	18.8%	0.001

**Conclusions:** Identifying scores that fall beyond the normal range of measurement error is essential for assessing statistically significant change. The RCIs presented in this paper allow for clinicians to make evidence-based decisions while treating iNPH.

## O15 Infantile flexible neuroendoscopic aqueductoplasty with stenting: a technical note and long-term follow-up

### Xuanwei Dong, Guoqiang Chen, Jiaping Zheng, Qing Xiao, Yiyang Huang

#### Department of Neurosurgery, Aviation General Hospital, Beijing, China

##### **Correspondence:** Jiaping Zheng - redleo@sina.com

*Fluids and Barriers of the CNS* 2019, **16(Suppl 3):**O15

**Introduction:** The aim was to technically review and explore long-term follow-up results of Aqueductoplasty with Stenting under Flexible Neuroendoscope in infantile obstructive hydrocephalus(A polyester fiber wrapped shunt is used as a new type of stent placed in the midbrain aqueduct).

**Methods:** From 2008 to 2010, the clinical data, surgical techniques and long-term effects of 14 infants with obstructive hydrocephalus treated by Flexible Neuroendoscopic Aqueductoplasty with Stenting were retrospectively analyzed.

**Results:** The 14 infants with a mean age of 5.71 ± 3.10 months (range, 2–11 months), and with a mean follow-up period of 62.64 ± 34.52 months (range, 9–121 months). After surgery, subdural effusion was observed in 4 infants (28.6%). These occurred without deaths or serious complications related to intracranial stent placement. 3 infants (21.4%) failed, of which 2 cases had a proximal aqueduct occlusion due to a short stent length, and 1 case intraluminal ependymal adhesion obstruction (1 case abandoned for the second surgical adjustment stent was unsuccessful, while the other 2 cases underwent shunt surgery).

**Conclusions:** Neuroendoscopic Aqueductoplasty with Stenting is a safe and effective method for the treatment of obstructive hydrocephalus in infants due to aqueductal stenosis or aqueductal membranous obstruction, but the operative indication should be obeyed strictly. A specialized stent material is needed. Higher case numbers and long-term follow-up data are required for final conclusions.

## O16 Evaluation of outcome after shunt surgery in iNPH by blinded assessment of pre- and postoperative videos—preliminary results

### Maria Ekblom, Dag Nyholm, Johan Virhammar

#### Department of Neuroscience, Neurology, Uppsala University, Uppland, Sweden

##### **Correspondence:** Johan Virhammar - johan.virhammar@gmail.com

*Fluids and Barriers of the CNS* 2019, **16(Suppl 3):**O16

**Introduction:** Few studies have used blinded assessment of outcome after shunt surgery in patients with idiopathic normal pressure hydrocephalus (iNPH). Assessment of gait is a common method to evaluate the result of shunt surgery since gait disturbance is often the most prominent symptom in iNPH. The aim of this study was to investigate the correlations between blinded assessments of outcome with the patients’ self-perceived rating and with outcome measured by the iNPH-scale.

**Methods:** Each patient (n = 16) was filmed on 2 occasions, before and at 3 months after surgery. One investigator, blinded to patient data and time of the recording, assessed the videos. The investigator tried to decide which video that was recorded before and after shunt surgery. The grade of improvement was rated on a 10-level scale and the characteristics of the gait disturbance were rated. Patients’ motor symptoms were graded according to the iNPH scale prior and post-surgery. Each patient graded their self-perceived improvement after surgery using a VAS-scale.

**Results:** The investigator correctly decided the time of the recording in 14 out of 16 patients (87.5%). There was a correlation between blinded grading of outcome and the patients’ self-perceived rating (r = 0.57, p < 0.01) and between blinded grading of outcome and change in iNPH-scale (r = 0.66, p < 0.01). There was no significant correlation between change in iNPH-score after surgery and the patients’ self-perceived rating of improvement.

**Conclusions:** Blinded grading of outcome correlated with the patients’ self-perceived improvement after shunt surgery and could be an alternative method to assess outcome in research studies.

## O17 Gait characterization after ventriculoperitoneal shunt placement in idiopathic normal pressure hydrocephalus

### Stacy R. Loushin^1^, Ryan M. Naylor^2^, Jeremy K. Cutsforth-Gregory^3^, David T. Jones^3^, Jonathan Graff-Radford^3^, Kenton R. Kaufman^1^, Benjamin D. Elder^2,4,5^

#### ^1^Motion Analysis Laboratory, Department of Orthopedic Surgery, Mayo Clinic, Rochester, Minnesota, 55902, USA; ^2^Department of Neurosurgery, Mayo Clinic, Rochester, Minnesota, 55902, USA; ^3^Department of Neurology, Mayo Clinic, Rochester, Minnesota, 55902, USA; ^4^Department of Orthopedic Surgery, Mayo Clinic, Rochester, Minnesota, 55902, USA; ^5^Department of Biomedical Engineering, Mayo Clinic, Rochester, Minnesota, 55902, USA

##### **Correspondence:** Benjamin D. Elder - elder.benjamin@mayo.edu

*Fluids and Barriers of the CNS* 2019, **16(Suppl 3):**O17

**Introduction:** While gait has been investigated using 3-D motion analysis pre and post shunt surgery, these gait parameters have not been compared to healthy persons. The objective was to characterize gait in patients with iNPH before and after VP shunt placement and compare to an unimpaired population.

**Methods:** Seven patients with a diagnosis of iNPH were analysed preoperatively and 4 weeks postoperatively. Patients walked barefoot and unassisted on a 10 m walkway. Kinematic data from a 10 camera system and kinetic data from five force plates embedded in the walkway were processed in Visual3D, and compared to laboratory normative data from 20 healthy young adults using t-tests.

**Results**: Preoperative temporal distance parameters were significantly different from healthy adults, and increased significantly following VP shunting, though did not reach values of healthy adults, and was much greater than the effect due to aging. Total sagittal plane range of motion (ROM) in the hip, knee and ankle showed decreased ROM compared to healthy adults (*p *< 0.05), and significant increases in hip and knee ROM were observed postoperatively again short of normal values. Ankle dorsiflexion and hip extension increased post shunt surgery, with maximum knee flexion showing a significant increase (*p *< 0.05).

**Conclusions**: Gait was objectively quantified pre and post VP shunt placement in patients with iNPH, with all measures trending toward normative values post surgery. Further studies comparing gait patterns pre and post VP shunt placement can provide insight into the efficacy of the surgical treatment and aide in guiding clinical practice.

## O18 A predictive classification for post-traumatic hydrocephalus following decompressive craniectomy for acute subdural haematoma: a london major trauma centre experience

### Amin Elyas^1^, Hasan Asif^1^, Curtis Offiah^2^, Chris Uff^1^

#### ^1^Department of Neurosurgery, The Royal London Hospital, E1 1BB, UK; ^2^Department of Radiology, The Royal London Hospital, E1 1BB, UK

##### **Correspondence:** Hasan Asif - hasan.asif@live.co.uk

*Fluids and Barriers of the CNS* 2019, **16(Suppl 3):**O18

**Introduction:** Post-traumatic hydrocephalus (PTH) is a known complication of acute subdural haematoma (ASDH) that has been managed with decompressive craniectomy (DC). The management usually requires permanent CSF diversion which in itself carries a risk of morbidity. The aetiology of PTH is unclear and has been suggested to be due to CSF flow disturbance secondary to the craniectomy but there is no correlation between DC size and incidence of PTH. We suggest a novel mechanism and predictive score for PTH in patients who have undergone DC for ASDH.

**Methods:** Retrospective review of prospectively maintained database of patients undergoing DC for ASDH between October 2016 and April 2019. Pre-DC computerised tomography imaging was used to assess for obliteration of four CSF spaces: right Sylvian fissure, left Sylvian fissure, right cerebral convexity and left cerebral convexity. Post-operative interval CT imaging was examined to assess for incidence of PTH.

**Results:** Fifty-eight patients (38M:20F) mean age 39.7 (± SD 15.2) underwent unilateral DC for ASDH, of these 38 (69.0%) went onto develop PTH. Our test cut off of “obliteration of 2 or more CSF spaces” was able to predict PTH with sensitivity of 97.5% (95% CI: 86.8–99.9) and a specificity of 50.0% (95% CI: 26.0–74.0) with 81.25% PPV and 90% NPV. Area under ROC curve for “obliteration of 2 or more CSF spaces” predicting PTH was 0.77 (P < 0.001).

**Conclusions:** Early identification of radiological features of acute or impending PTH may allow for appropriate and timely CSF diversion facilitating reduction of morbidity, early discharge, cranioplasty and rehabilitation.

## O19 Inpatient health care burden of adult idiopathic intracranial hypertension

### Iris Emerman^1^, Jiangxia Wang^2^, Abhay Moghekar^1^

#### ^1^Department of Neurology, Johns Hopkins Medical Institutions, Baltimore, Maryland, USA; ^2^Department of Biostatistics, Johns Hopkins Biostatistics Center, Baltimore, Maryland, USA

##### **Correspondence:** Abhay Moghekar - am@jhmi.edu

*Fluids and Barriers of the CNS* 2019, **16(Suppl 3):**O19

**Introduction:** Idiopathic intracranial hypertension (IIH) is a rare cerebrospinal fluid (CSF) disorder with a substantial cost to those who suffer from it. This study aims to quantify trends in the financial burden of IIH-related inpatient admissions, allowing researchers and policy-makers to better predict the future burden IIH may pose on the US healthcare system.

**Methods:** Data from 2001 to 2014 were extracted from the National Inpatient Sample (NIS) database, the largest publicly available all-payer inpatient health care database in the US. Our analyses were conducted on adult inpatient admissions directly relating to IIH, as determined by International Classification of Diseases, Ninth Revision (ICD-9-CM) codes. Analyses were conducted using Stata (version 14.2).

**Results:** The number of IIH-related inpatient admissions in the United States increased 130% from 2001 to 2014. In contrast, the obesity rate in the general adult population has increased approximately 24% over the same time period. Hospital charges for IIH-related admissions were an estimated total of $280,000,000 for IIH-related inpatient admissions in 2014 with a median charge per visit of $32,000.

**Conclusions:** While prior research has indicated that rising obesity rates are driving increased rates of IIH, our results suggest that IIH-related inpatient admissions are increasing at a faster rate than the obesity rate of the general population. Our analyses indicate that variables associated with higher cost of IIH-related inpatient admissions include length of stay, number of procedures, number of comorbid diagnoses, sex (male inpatient visits are typically more expensive), non-routine discharge, and ventriculo-peritoneal (VP) shunt replacements.

## O20 Patient characteristics affecting inpatient care of adult idiopathic intracranial hypertension

### Iris Emerman^1^, Jiangxia Wang^2^, Abhay Moghekar^1^

#### ^1^Department of Neurology, Johns Hopkins Medical Institutions, Baltimore, Maryland, USA; ^2^Department of Biostatistics, Johns Hopkins Biostatistics Center, Baltimore, Maryland, USA

##### **Correspondence:** Abhay Moghekar - am@jhmi.edu

*Fluids and Barriers of the CNS* 2019, **16(Suppl 3):**O20

**Introduction:** Idiopathic intracranial hypertension (IIH) is a rare cerebrospinal fluid (CSF) disorder which can lead to headaches and vision loss. Studies examining patient characteristics of IIH are often limited in sample size. The aim of this study was to describe patient characteristics for IIH-related inpatient care in a national dataset.

**Methods:** Data from 2001 to 2014 were extracted from the National Inpatient Sample (NIS) database, the largest publicly available all-payer inpatient health care database in the US. We assessed adult inpatient admissions directly relating to a diagnosis of IIH, determined by International Classification of Diseases, Ninth Revision (ICD-9-CM) codes. Survey logistic and Poisson regression analyses were conducted using Stata (version 14.2).

**Results:** Women aged 18–44 accounted for 79% of adult inpatient IIH-related admissions records. Records of Black patients and male patients were more strongly associated with impaired vision (Odds Ratio (OR) = 1.37, CI: 1.20–1.58; OR = 1.39, CI: 1.15–1.69, respectively) when accounting for age, insurance, obesity status and reporting year. Number of procedures, number of comorbid diagnoses, Medicaid insurance, and non-routine discharge were all significantly associated with longer length of stay (Incidence Rate Ratio (IRR) = 1.22, 95% Confidence Interval (CI) = 1.16–1.28; IRR = 1.07, CI = 1.05–1.08; IRR = 1.19, CI = 1.05–1.35; IRR = 1.47, CI = 1.28–1.68, respectively).

**Conclusions:** Our model provides information on the patient characteristics associated with longer inpatient visits. Our results clarify previously conflicting reports about the effect of sex and race on IIH-related morbidity.

## O21 Pediatric hydrocephalus in the united states as measured through inpatient care

### Iris Emerman^1^, Aamir Khan^1^, Jiangxia Wang^2^, Abhay Moghekar^1^

#### ^1^Department of Neurology, Johns Hopkins Medical Institutions, Baltimore, Maryland, USA; ^2^Department of Biostatistics, Johns Hopkins Biostatistics Center, Baltimore, Maryland, USA

##### **Correspondence:** Abhay Moghekar - am@jhmi.edu

*Fluids and Barriers of the CNS* 2019, **16(Suppl 3):**O21

**Introduction:** This study was designed as an update on the state of hospital care for children with hydrocephalus. Our aims were to measure inpatient healthcare utilization of children with hydrocephalus in the US; assess trends in surgical interventions for pediatric hydrocephalus; describe patient, hospital, and hospitalization characteristics for inpatient admissions related to pediatric hydrocephalus; and determine characteristics associated with pediatric mortality among children admitted with hydrocephalus.

**Methods:** Data from 2000, 2003, 2006, 2009, and 2012 were extracted from the Kid’s Inpatient Databases (KID) database, a nationally representative database of pediatric inpatient admissions in the US. We assessed pediatric inpatient admissions with a diagnosis of hydrocephalus, as determined by International Classification of Diseases, 9th Revision, Clinical Modification (ICD-9-CM) codes.

**Results:** Each year there were 24,000–26,000 admissions, 266,000–315,000 hospital days, and total hospital charges of $1.2–2.8 billion for pediatric hydrocephalus. Hydrocephalus accounted for 0.35–0.41% of all pediatric hospital admissions in the US but for 1.1–1.3% of all pediatric hospital days and 1.8–2.2% of all pediatric hospital charges. There has been no statistically significant change in mortality rates over time for pediatric hydrocephalus.

**Conclusions:** Inpatient admissions for children with hydrocephalus account for a disproportionate share of hospital days and healthcare dollars in the US. Despite advances in shunt technology and the advent of newer procedures such as endoscopic third ventriculostomy, mortality rates for pediatric hydrocephalus do not appear to have decreased.

## O22 Beyond the pillars of hercules: transaqueductal navigation to manage hydrocephalus due to blood clots, membranes, and arachnoid cysts

### Alberto Feletti, Giuliano Giliberto, Elisa Moriconi, Adelaide Valluzzi, Stavros Dimitriadis, Riccardo Stanzani, Giacomo Pavesi

#### Department of Neurosurgery, University Hospital of Modena, Italy

##### **Correspondence:** Alberto Feletti - alberto.feletti@gmail.com

*Fluids and Barriers of the CNS* 2019, **16(Suppl 3):**O22

**Introduction:** Intraventricular neuroendoscopy has rapidly developed over the recent years, due to its effectiveness in the management of several ventricular and paraventricular pathologies with a minimally invasive approach. Although many issues can be solved using a rigid endoscope, which has better image quality and more complete instrumentation, some procedures can be performed only with a flexible scope.

**Methods:** Between 2014 and 2018, 88 patients underwent neuroendoscopic procedures with a flexible scope (Karl Storz, Tuttlingen, Germany). Patients who underwent transaqueductal navigation were selected. For all these cases, preoperative imaging, intraoperative recordings, and postoperative imaging were reviewed. Preoperative clinical data were compared with postoperative outcomes.

**Results:** With the use of the flexible scope, we were able to completely aspirate intraventricular clots in patients affected by tetraventricular hemorrhage. We could effectively manage arachnoid cysts in the fourth ventricle and even in the cisterna magna from a precoronal paramedian burr hole. It was also possible to detect membranous obstructions of the cerebral aqueduct, treating hydrocephalus with endoscopic perforation of the membranes.

**Conclusions:** Despite the lower image quality compared to the rigid scope, and the lack of dedicated instrumentation, only the flexible scope allows complete navigation of the cerebral aqueduct and fourth ventricle for cyst fenestration or complete aspiration of intraventricular hemorrhage, using a single burr hole access. A non-stenotic aqueduct can be safely navigated by a well-experienced neuroendoscopist. In our experience, there is no risk of damage to the fornix using a flexible scope.

## O23 Neuroendoscopic aspiration of intraventricular hemorrhage

### Alberto Feletti, Adelaide Valluzzi, Elisa Moriconi, Giuliano Giliberto, Annette Puzzolante, Giorgio Casoli, Giacomo Pavesi

#### Department of Neurosurgery, University Hospital of Modena, Italy

##### **Correspondence:** Alberto Feletti - alberto.feletti@gmail.com

*Fluids and Barriers of the CNS* 2019, **16(Suppl 3):**O23

**Introduction:** The amount of intraventricular blood is a strong negative prognostic predictor on outcome. Therefore, massive intraventricular hemorrhages (IVHs) require aggressive and rapid management to decrease intracranial hypertension. Flexible neuroendoscopy can be used for intraventricular clots removal, allowing for rapid reduction of intracranial pressure and early removal of external ventricular drainage.

**Methods:** We present the series of 22 patients who were treated to remove IVH at our Institution. Neuroendoscopy is indicated when IVH causes hydrocephalus, brainstem compression, and ultimately intracranial hypertension. If aneurysm is the primary cause of IVH, it must be secured before proceeding with neuroendoscopic removal of intraventricular clots.

**Results:** All ventricles could be explored and cleared from clots, in order to restore patency of CSF pathways. The external ventricular drainage (EVD) was always left in the ventricle after surgery, although in most of the cases early removal was possible. The length of stay in ICU was reduced compared to patients who were treated only with EVD.

**Conclusions:** Early neuroendoscopic removal of blood casting from the lateral to the fourth ventricle is a feasible approach, allowing in most instances the rapid improvement of the IVH, the decrease of EVD dependency, and shorter ICU stay.

## O24 Revisions after primary shunt insertion by catheter and valve type: a registry based study

### Rocío Fernández-Méndez^1,2,3^, Hugh K. Richards^1,2^, Helen M. Seeley^1,2^, John D. Pickard^1,2,3^, Alexis J. Joannides^1,2,3^

#### ^1^Department of Clinical Neurosciences, University of Cambridge, UK; ^2^United Kingdom Shunt Registry (UKSR), Cambridge, UK; ^3^NIHR Brain Injury MedTech Co-operative, NIHR, Cambridge, UK

##### **Correspondence:** Rocío Fernández-Méndez - rociofmendez.inv@gmail.com

*Fluids and Barriers of the CNS* 2019, **16(Suppl 3):**O24

**Introduction:** The aim of this study was to determine frequency and characteristics of revisions of primary cerebrospinal fluid (CSF) shunts in a large patient cohort from the UK Shunt Registry (UKSR).

**Methods:** A retrospective, multi-centre study was conducted based on 10 years’ UKSR data (2004–2013). Descriptive statistics were calculated stratified by age group. Revision rates and characteristics were compared by antibiotic-impregnated catheters (AIC) vs non-AIC; and programmable vs fixed valves.

**Results:** There were 20947 primary procedures during the 10-year study-period. First-year revision rates were 31.0%, 25.2% and 17.4% in infants, children and adults respectively. By AIC vs non-AIC, these rates were: 29.1% vs 29.8%; 23.5% vs 24.8%; 16.3% vs 17.3%, among the respective age groups. In infants with AIC there was a lower proportion of revisions for shunt infection (9.3% vs 15.0%, P = 0.049), but a higher proportion for underdrainage (34.4% vs 22.7%, p = 0.004) and migration (3.8% vs 0.5%, p = 0.025), as compared to those with non-AIC. In children with AIC there was a lower proportion of revisions for shunt infection (3.8% vs 7.2%, P = 0.046) and fracture (1.2% vs 3.4%, p = 0.043). In adults, however, the distributions of the reasons for revision under study did not show statistically significant differences by catheter type. By programmable vs fixed valve, first-year revision rates were 22.7% vs 25.0%, 18.8% vs 20.8%, and 10.1% vs 16.1%, in infants, children and adults respectively.

**Conclusions:** Primary CSF shunts using antibiotic-impregnated catheters or programmable valves are associated with lower overall first-year revision rates. Revision characteristics differed between AICs and non-AICs.

## O25 Non-invasive visualisation of shunt obstruction with constructive interference in steady state (CISS) MRI sequences

### Aimee Goel, Claudia L. Craven, Hasan Asif, Linda D’Antona, Simon Thompson, Lewis Thorne, Laurence D. Watkins, Ahmed K. Toma

#### Victor Horsley Department of Neurosurgery, National Hospital for Neurology and Neurosurgery, London, WC1N 3BG, UK

##### **Correspondence:** Aimee Goel - aimee.goel@hotmail.com

*Fluids and Barriers of the CNS* 2019, **16(Suppl 3):**O25

**Introduction:** Common methods of assessment to determine proximal shunt lumen obstruction include shunt tapping, shunt-o-gram, infusion studies, ICP monitoring and intra-operative assessment. Such methods are invasive and therefore carry an associated infection risk.

CT scans and T2 weighted MRI sequences are used to evaluate for proximal shunt obstruction by evaluating ventricular size, however ventricular size is not always affected in shunt malfunction. Constructive Interference in Steady State (CISS) is a 3D gradient echo MRI sequence that accentuates T2 values between cerebrospinal fluid (CSF) and pathological structures. We hypothesise that a coronal CISS sequence may be a novel non-invasive sequence to demonstrate proximal shunt lumen obstructions

**Methods:** Three patients with diagnosis of IIH with parieto-occipital shunts in situ had suspected proximal catheter blockage. Both underwent standard MR imaging with additional CISS sequences to assess lumen patency.

**Results:** In all three cases, the CISS sequence image of the catheter lumen demonstrated a clear obstruction with choroid plexus. This was subsequently confirmed intra-operatively during surgical revision. In all cases, the CISS sequence demonstrated the location of shunt obstruction as well as the segment and length of shunt affected. CISS sequences were able to provide the CSF-shunt differentiation that T2 sequences were not.

**Conclusions:** CISS sequences may be a valuable non-invasive tool for identifying proximal shunt obstruction.

## O26 Regional scalp blockade for painless removal of ICP bolts: a technical note and patient reported outcomes

### Aimee Goel, Hasan Asif, Pranoy Das, Claudia Craven, Lewis Thorne, Laurence D. Watkins, Ahmed K. Toma

#### Victor Horsley Department of Neurosurgery, National Hospital for Neurology and Neurosurgery, London, WC1N 3BG, UK

##### **Correspondence:** Hasan Asif - hasan.asif@live.co.uk

*Fluids and Barriers of the CNS* 2019, **16(Suppl 3):**O26

**Introduction:** Intracranial pressure (ICP) monitoring through insertion of a bolt is a useful tool for the purpose of diagnosing and treating disorders of cerebrospinal fluid (CSF) dynamics and hydrocephalus. Typically patients report severe discomfort on bolt removal. We determined the feasibility of implementing a commonly used form of scalp anaesthesia for bolt removal, comparing efficacy and safety against current practise.

**Methods:** Prospective case cohort of patients undergoing removals of diagnostic ICP bolts between June 2017 and April 2019. Two groups were identified: “A” receiving oral analgesia only and “B” receiving ipsilateral supraorbital and supratrochlear nerve blocks with 5 ml of 1% lidocaine. Subjective outcomes were collected by review of patient-completed questionnaires with white space, yes–no and 5-point Likert scale questions.

**Results:** Eighty-five patients were fitted with ICP monitoring bolts (32M:53F, mean age 42.7 ± 16.0). Fifty-four were removed with oral analgesia only (group A) and 31 were removed with oral and regional anaesthesia (group B). Overall removal experience was 3/5 for group A and 4/5 for group B (p < 0.01). Thirty-six (70.6%) patients would have preferred a scalp block in group A. In group A, patients reported the best part of removal was “having it out” and worst was “pain and slowness”. In group B, the best part of removal was that “it was painless” and worst part was during anaesthetic infiltration.

**Conclusions:** Regional nerve blocks to the ipsilateral supraorbital and supratrochlear nerves are a safe and effective adjuvant for the painless removal of frontal ICP monitoring bolts.

## O27 Discordant CSF and PET Alzheimer disease biomarkers in ADNI related to CSF dynamics

### Jeff Gunter, Ryan Townley, Heather Wiste, Matthew Senjem, David Jones, Jonathan Graff-Radford, Neill Graff-Radford, Clifford Jack, Jr

#### Mayo Clinic, Rochester, Minnesota, 55905, USA

##### **Correspondence:** Jeff Gunter - gunter.jeffrey@gmail.com

*Fluids and Barriers of the CNS* 2019, **16(Suppl 3):**O27

**Introduction:** CSF and PET imaging biomarkers for brain amyloid are known to be discordant in 5–10% of elderly individuals participating in Alzheimer disease and natural history studies. Discordance is also well established in clinical NPH. Data from the ADNI study (not an NPH cohort) are dichotomized using two independent imaging signatures associated with DESH or NPH to investigate if CSF and PET amyloid values differ between the dichotomized groups.

**Methods:** ADNI-2 participants were dichotomized by FDG PET striatal hypometabolism (R. Townley et al. 10.1016/j.nicl.2018.02.031). Kolmogorov–Smirnov (K–S) tests were used to test for differences in CSF Aβ_42_, pTau, tTau, and Florbetapir PET SUVR distributions between the dichotomized groups. Independently, the data were also dichotomized based on a structural pattern matching score for the DESH imaging signature (CDESH, N. Gunter et al. 10.1016/j.nicl.2018.11.015) and K–S tests were performed.

**Results:** Florbetapir SUVR distributions were not significantly different for either dichotomization method but the CSF markers were significantly different for both (p < 0.01). Furthermore, distributions of CSF Aβ_42_ to pTau ratio were, as in PET, not different based on dichotomization. For either dichotomization, the fraction of participants with discordant CSF and Florbetapir levels was higher in the abnormal groups.

**Conclusions**: CSF Aβ_42_ and tau measures differ when dichotomized by imaging-based signatures associated with NPH detected using FDG PET or structural MRI. That is not the case for PET assessment of brain amyloid. Ratios of CSF markers are also not different which may suggest, for example, a dilution effect or impeded clearance from brain to CSF.

## O28 Difference of risk factors and clinical symptoms between idiopathic and symptomatic normal pressure hydrocephalus

### Rongrong Hua^1^, Jinwu Zhu^2^, Chunyan Liu^1^, Yan Xing^1^

#### ^1^Department of Neurology, Aviation General Hospital, Beijing, 100012, China; ^2^Aviation Medical Engineering Center of Aviation General Hospital, Beijing, 100012, China

**Correspondence:** Yan Xing - hkzyysjnk@sina.com

*Fluids and Barriers of the CNS* 2019, **16(Suppl 3):**O28

**Introduction:** Normal pressure hydrocephalus (NPH) is a critical brain disorder with gait failure, cognition impairment and urinary incontinence as its core symptoms. The high morbidity and mortality in older patients lead to a heavy economic and social burden. The diagnosis of NPH, especially the idiopathic normal pressure hydrocephalus (iNPH) is a challenge for the diversity and of coexistence of symptoms.

**Methods:** 43 patients with NPH and 129 community residents as control were recruited in this study and accepted a face-to-face questionnaire about risk factors, a clinical examination and magnetic resonance imaging (MRI) test during the visit.

**Results:** The prevalence of stroke in NPH patients was significantly higher than that in control group. There were 6 patients with symptomatic normal pressure hydrocephalus (sNPH), whose causes were brain trauma, hemorrhage and meningitis. The vascular risk factors were similar in sNPH and iNPH group, in which the percentage of hypertension was significantly higher than control. The major first symptom of iNPH was gait disorder or cognitive impairment, which accounted for 78.38% and 21.62% respectively. The percentage of coexistence of three or two symptoms in iNPH were 45.95% and 40.54%, both of which were significantly higher than single symptom occurrence. The coexistence of gait disorder and cognitive impairment was 27.03%, although 10.81% patients had gait disorder and urinary incontinence simultaneously. Only 1 patient had cognitive impairment and urinary incontinence. Unexpectedly, there was no significant difference for sex or age between iNPH patients with one, two and three symptoms or with different first symptoms.

**Conclusions:** Hypertension and history of stroke may be the major risk factors for NPH. There was no difference in cardiovascular risk factors between sNPH and iNPH. The results revealed different first and coexisted symptoms in iNPH patients, which may be parallel to each other.

## O29 Identification of normal pressure hydrocephalus by disease-specific patterns of brain stiffness and damping ratio

### John Huston^1^, Matthew C. Murphy^1^, Arvin Arani^1^, Fredric B. Meyer^2^, Armando Manduca^3^, Kevin J. Glaser^1^, Richard L. Ehman^1^

#### ^1^Department of Radiology, Mayo Clinic, Rochester, Minnesota, 55905, USA; ^2^Department of Neurologic Surgery, Mayo Clinic, Rochester, Minnesota, 55905, USA; ^3^Department of Physiology and Biomedical Engineering, Mayo Clinic, Rochester, Minnesota, 55905, USA

##### **Correspondence:** John Huston - jhuston@mayo.edu

*Fluids and Barriers of the CNS* 2019, **16(Suppl 3):**O29

**Introduction:** Altered brain biomechanics, which can be measured noninvasively by magnetic resonance elastography (MRE), represent one hypothesis of normal pressure hydrocephalus (NPH) pathogenesis. Here we evaluated the accuracy of MRE-based viscoelasticity measurements to discriminate patients with NPH from both cognitively normal (CN) subjects and patients with probable Alzheimer’s disease (AD).

**Methods:** Thirty-three NPH, 44 CN and 8 AD subjects were scanned after obtaining IRB approval and written informed consent. MRE exams were collected with a spin-echo EPI pulse sequence (60-Hz motion, 3-mm isotropic final image resolution). Stiffness and damping ratio maps were computed using neural network inversion. A voxel-wise analysis was performed to compute maps of stiffness and damping ratio changes due to NPH. P < 0.025 was considered significant for each mechanical property (cluster-level family-wise error corrected). Each subject’s MRE result was summarized by computing the correlation coefficient between that subject’s age- and sex-corrected maps and the estimated group map while leaving out that subject.

**Results:** Subjects with NPH exhibited a concentric pattern of stiffness changes with periventricular softening and stiffening near the dural surface. Damping ratio was also significantly decreased in NPH subjects. The correlation of an individual’s mechanical maps to the estimated group maps discriminated NPH subjects both from CN (area under receiver operating characteristic curve [AUROC] = 0.98) and AD (AUROC = 0.98) subjects.

**Conclusions:** NPH subjects exhibit unique patterns of mechanical properties and this analysis helps reconcile previous, seemingly discrepant results from MRE studies of NPH using different regions of interest.

## O30 Reducing the risks of proximal and distal shunt failure in adult hydrocephalus (the shout-Qi initiative)

### Albert M. Isaacs^1^, Chad Ball^2^, Geberth Urbaneja^3^, Jarred Dronyk^3^, Heather Yong^4^, Rich Holubkov^5^, Mark G. Hamilton^1^

#### ^1^Division of Neurosurgery, University of Calgary, Alberta, Canada; ^2^Department of Surgery, University of Calgary, Alberta, Canada; ^3^Adult Hydrocephalus Program, University of Calgary, Alberta, Canada; ^4^Cumming School of Medicine, University of Calgary, Alberta, Canada; ^5^Department of Biostatistics, University of Utah School of Medicine, Salta lake City, USA

##### **Correspondence:** Albert M. Isaacs - akm.isaacs@gmail.com

*Fluids and Barriers of the CNS* 2019, **16(Suppl 3):**O30

**Introduction:** Shunt failures are common and subject patients to multiple surgeries and decreased quality of life. A Shunt Outcomes Quality Improvement (**S**h**O**ut-**QI**) initiative was implemented to reduce shunt failure incidence (**SFI**) through: (1) neuronavigation-assisted proximal catheter insertion; and (2) laparoscopy-guided distal catheter anchoring over the liver dome to drain into the right upper quadrant (RUQ), away from omentum and common shunt obstruction-prone debris. A prospective cohort study tested the hypothesis *neuronavigation and laparoscopy*-*guided VP shunt insertion (VPSI) will reduce incidence of shunt failure.*

**Methods:** “Pr**e-**S**hOut**” and “Post**-S**h**O**ut” groups of patients were assessed, which included those who had their initial VPSIs done before or after the **S**h**O**ut-QI initiative, and without or with neuronavigation/laparoscopy, respectively. A 3-point CT index assessed proximal catheter placement, postop X-rays confirmed distal catheter placement, and a standardized protocol determined the primary outcome (SFI) as any return to surgery for shunt revision.

**Results:** 244 patients (97 Pre**-S**h**O**ut, 147 Post**-S**h**O**ut), mean age 73 years, were observed for ~ 4 years. Neuronavigation improved proximal catheter placement accuracy by 20% (*p *< .001), and 90% of laparoscopy-guided distal catheters drained into the RUQ. SFI occurred in 57% vs 23%, with a mean duration of 380 vs 283 days to revision surgery, in the Pre**-S**h**O**ut and Post**-S**h**O**ut groups, respectively (*p *= .008).

**Conclusions:** Adult SFI may be reduced by improving the accuracy of proximal catheter placement with neuronavigation and reducing the risk of distal catheter failure with neuronavigation-guided placement. Further studies are necessary to assess the effect of these interventions on long-term patient outcomes.

## O31 Transesophageal echocardiography facilitates ventriculo-atrial shunt placement to reduce risk of perioperative complications

### Albert M. Isaacs^1^, Danae Krahn^2^, Andrew M. Walker^2^, Heather Hurdle^2^, Mark G. Hamilton^1,3,4^

#### ^1^Division of Neurosurgery, Department of Clinical Neuroscience, University of Calgary, Canada; ^2^Department of Anesthesia, University of Calgary, Canada; ^3^Adult Hydrocephalus Program, Department of Clinical Neuroscience, University of Calgary, Canada; ^4^Department of Neuroscience, Washington University School of Medicine, St. Louis, Missouri, USA

##### **Correspondence:** Albert M. Isaacs - akm.isaacs@gmail.com

*Fluids and Barriers of the CNS* 2019, **16(Suppl 3):**O31

**Introduction:** Determining an optimal location within the right atrium for placement of the distal ventriculo-atrial (VA) shunt catheter offers several operative challenges that place patients at risk for perioperative complications and downstream VA shunt failure. Utilizing transesophageal echocardiography (TEE) guidance to place distal VA shunt catheters may help to circumvent these risks.

**Methods:** A retrospective review of all consecutive patients who underwent VA shunt procedures between December 19, 2016 and January 22, 2019, during which time intraoperative TEE used for shunt placement was performed. Data on the time required for shunt placement and total procedure time, baseline echocardiography findings, and short- and long-term complications of shunt placement were assessed.

**Results:** Thirty-three patients underwent VA shunt procedures, with a median follow-up time of 250 [88–412] days. The only immediate complication related to shunt placement or TEE use, was transient ectopy in one patient. The mean time for atrial catheter insertion was 12.6 ± 4.8 min. Right heart catheters were inserted between the right atrium (RA)-superior vena cava (SVC) junction and 22 mm within the RA in all but three procedures. 7/33 patients (21%) underwent shunt revision. Indications for revisions included distal clots, proximal obstruction, positive blood culture and shunt valve revision. No other complications of VA shunt insertion were reported.

**Conclusions:** VA shunt insertion using TEE allows for precise distal catheter placement. Early patient experience confirms this technique has a low complication rate. However, further studies are needed to assess long-term patient outcomes.

## O32 Agreement in gait assessment with video-rating in patients with idiopathic normal pressure hydrocephalus

### Masatsune Ishikawa^1,2^, Shigeki Yamada^2,3^, Kazuo Yamamoto^3^, Hachiro Moriguchi^4^

#### ^1^Rakuwa Villa Ilios, Kyoto, Japan; ^2^Normal pressure hydrocephalus Center, Otowa Hospital, Kyoto, Japan; ^3^Department of Neurosurgery, Juntendo Tokyo Koto Geriatric Medical Center, Japan; ^4^Rehabilitation Center, Otowa Hospital, Kyoto, Japan

##### **Correspondence:** Shigeki Yamada - shigekiyamada39@gmail.com

*Fluids and Barriers of the CNS* 2019, **16(Suppl 3):**O32

**Introduction:** Gait disturbance is a major symptom of the idiopathic normal pressure hydrocephalus (iNPH), and it is assessed by many personnel including doctors and rehabilitation staff (rehabs). Here, agreement among multiple raters was examined using video-based gait analysis in patients with iNPH.

**Methods:** Fifteen patients with definite iNPH were enrolled. Timed go and test (TUG) was done twice in all patients. The assessment of gait was done in 8 patterns including freezing, shuffling, wide-base and short-step. On the video-rating method, seven staff of 2 doctors and 5 rehabs assessed simultaneously. The iNPH grading scale (GS) was also scored. Agreement study was done with the Krippendorff alpha. The alpha value ≥ 0.67 was defined as good.

**Results:** In the first assessment, no patterns were regarded as good in both all 7 staff and 4 staff (2 doctors and 2 rehabs), while agreement between 2 doctors and 2 rehabs, respectively, was good in some patterns. Good agreement was observed in the GS score both in all 7 staff and the 4 staff. After making consensus in gait patterns of iNPH, the second assessment was done. The consensus-making was helpful to improve agreement in some of patterns and GS in 4 staffs, but not in 7 staffs.

**Conclusions:** Agreement study using Krippendorff alpha among multiple raters revealed that the agreement of gait patterns in the iNPH was not good with multiple raters, while the GS was useful in scale because of good agreement even for multiple raters.

## O33 On-line follow-up of hydrocephalus

### Marianne Juhler

#### Aarhus University Hospital, 8200, Hellerup, Denmark

##### **Correspondence:** Marianne Juhler - marianne.juhler@gmail.com

*Fluids and Barriers of the CNS* 2019, **16(Suppl 3):**O33

**Introduction**: Hydrocephalus is a chronic disease. Most patients are treated with a shunt implant regardless of age. Numerous publications attest to the limited durability of this treatment. Endoscopic fenestration (ETV) is possible as an alternative in case of visibly obstructive hydrocephalus. Recent reports suggest that the durability of ETV is superior to shunt survival, but there are so far no long term statistics. In addition, cognitive and physical handicaps are often present. However, there is no evidence-based consensus for recommended follow-up, and complications or shortcomings to treatment are often not anticipated or prevented by short out-patient visits.

**Methods:** We have developed an on-line systematic questionnaire for follow-up of patients with hydrocephalus. The questionnaire is organized into domains (shunt-dysfunction symptoms, cognition, physical function, quality of life, etc.) with 3–6 validated indicator questions for each domain. Answers are flagged as “green” for no problems; “yellow” as possible need of clinical assessment or “red” as very likely/definite need of clinical assessment. All-green answers continue on-line follow-up; one or more “yellow answers” are interviewed by telephone and called in if necessary; one or more “red answers” result in an outpatient consultation.

**Results**: Screening of questionnaires has been successfully integrated into the work process in the hydrocephalus out-patient clinic. Unnecessary visits with absence from school/work/daily activities are avoided. Clinical follow-up has become systematically standardized.

**Conclusions:** We believe that hydrocephalus follow up by on-line questionnaires is applicable in a much wider context. It may be particularly useful in cases of large geographical distances between home and clinic.

## O34 Tips for LP shunt surgery with fluoroscopic-guided paramedian approach

### Yoshinaga Kajimoto^1^, Adam Tucker^2^

#### ^1^Department of Neurosurgery, Osaka Medical College, Takatsuki, 569-8686, Japan; ^2^Department of Neurosurgery, Japanese Red Cross Society Red Cross Hospital Kitami, Hokkaido, 090-8666, Japan

##### **Correspondence:** Yoshinaga Kajimoto - neu039@osaka-med.ac.jp

*Fluids and Barriers of the CNS* 2019, **16(Suppl 3):**O34

**Introduction:** Recently, we have reported the usefulness of fluoroscopic-guided paramedian approach in LP shunt surgery. In this method, the catheter insertion success rate is 100%, and complications such as catheter insertion difficulty and nerve root pain are rare. This paper reports on practical tips for this method.

**Methods:** LP shunt selection checklist: The main cause of nerve root pain was the passage of the subarachnoid catheter into the narrow spinal canal. Therefore, in cases with L2/3 or L1/2 spinal stenosis where the catheter passes, VP shunt surgery should be selected because of the high risk of nerve root pain. No nerve root pain has occurred since adopting this checklist. Skin marking method (2345 and 60 method): It is not easy for beginners to decide in which direction the puncture should be corrected even under fluoroscopy. This method facilitates the right correction of the puncture direction. Confirmation of the catheter in the fluoroscopic image: 100% prevention of caudal insertion of the catheter is possible. Valve placement to the optimal site and depth: Thick subcutaneous fat on the valve makes post-operative valve resetting and pumping reservoir difficult. To prevent these, the paramedian approach is performed from the contralateral side, and a valve is placed on the paraspinal muscle under a thin subcutaneous fat. One-pass passer method: This method prevents the valve from getting stuck during its insertion. Insert a peritoneal catheter and valve at the same time as making a subcutaneous pocket with a plate-like shunt passer.

## O35 Amyloid-β oligomers in cerebrospinal fluid distinguish idiopathic normal pressure hydrocephalus from other neurodegenerative diseases

### Kaito Kawamura^1^, Masakazu Miyajima^1,2^, Madoka Nakajima^1^, Ikuko Ogino^1^, Chihiro Akiba^1,2^, Jyo Kanpyo^1^, Chihiro Kamohara^1^, Wei Meng^1^, Hajime Arai^1^

#### ^1^Department of Neurosurgery, Graduate School of Medicine, Juntendo University, Tokyo, Japan; ^2^Department of Neurosurgery, Juntendo Tokyo Koto Geriatric Medical Center, Japan

##### **Correspondence:** Kaito Kawamura - k-kawamu@juntendo.ac.jp

*Fluids and Barriers of the CNS* 2019, **16(Suppl 3):**O35

**Introduction:** Idiopathic normal pressure hydrocephalus (iNPH) sometimes mimics the clinical symptoms of other neurodegenerative diseases. Various biomarkers have been used to distinguish iNPH, but none have been entirely successful. We hypothesized that, in iNPH, stagnation of cerebrospinal fluid (CSF) turnover may cause amyloid-β peptide (Aβ) accumulation, which may be improved by shunt placement. Therefore, measuring high molecular weight Aβ42 oligomer (HMAβ) with at least nine subunits (≥ 30 kDa), could support differentiation of iNPH from Alzheimer’s disease (AD), Parkinson’s disease (PD), and progressive supranuclear palsy (PSP). It could also elucidate changes in amyloid aggregates in CSF after shunt placement.

**Methods:** Fifty-three patients with NPH were included: healthy controls (HC, 30), AD (16), PD (14), and PSP (14). All patients with NPH had lumbo-peritoneal shunt (LPS); CSF samples were taken before and 1 year after surgery to measure phosphorylated tau (p-Tau), Aβ42, toxic Aβ42 conformer, and HMAβ via sandwich ELISA. NPH patients were divided into four subgroups: iNPH (18), NPH with AD pathology (17), NPH with Parkinson’s spectrum (PS) (14), and NPH with AD pathology and PS (5), according to the p-Tau level and ^123^I-ioflupane SPECT.

**Results:** iNPH had significantly higher levels of HMAβ (7.26 ± 0.88 pM) than HC (3.38 ± 1.45 pM), AD (6.01 ± 1.18 pM), PD (3.33 ± 0.90 pM), and PSP (4.46 ± 0.98 pM). HMAβ levels in iNPH visibly declined with advanced CSF drainage after shunt placement (5.51 ± 2.13 pM).

**Conclusions:** CSF HMAβ level supported differential diagnosis of iNPH. Aβ accumulation may be due to CSF stagnation and shunt placement may improve HMAβ clearance in iNPH patients.

## O36 Facilitated ventricle catheter placement during shunt surgery with mixed reality

### Uwe Kehler^1^, Simon Furrer^2^, Nadja Parfenov^2^, Ahmed El-Allawy^1^

#### ^1^Department of Neurosurgery, Asklepios Klinik Altona, Hamburg, Germany; ^2^apoQlar, Hamburg, Germany

##### **Correspondence:** Uwe Kehler - u.kehler@asklepios.com

*Fluids and Barriers of the CNS* 2019, **16(Suppl 3):**O36

**Introduction:** Implantation of ventricular catheters for hydrocephalus shunts still remains a source of complications due to suboptimal or even wrong placement. The risk grows with smaller ventricles. Additional partially time-consuming tools like neuronavigation, Thomale guide, endoscopy etc. were used to improve the ventricular catheter placement. However, a simple, easy applicable tool is necessary for acceptance by everybody to improve the overall outcome. The solution might be mixed reality—an overlap of virtual reality with reality—our very first experience is presented here.

**Methods:** The new VSI-technology for HoloLens allows the surgeon to see the bore hole of the skull with the surface of the brain as well as the (holographic) superposed ventricles in the depth at the same time. With this visualization he is able to puncture the ventricle without the help of landmarks to two different planes of reference.

**Results:** The first shunt surgeries with the augmented/mixed reality demonstrated the feasibility. The technique is convincing, although the method is new; only few minutes were needed for implementing the system.

**Conclusions:** The technique of safe ventricular puncture is convincing and can be easily integrated in the surgical work-up to make shunt surgery safer. The potential is obvious in all surgical fields to guide needles, screws and other implants. The potential of reducing the overall complication rate of ventricle puncture of course has to be proven in randomized prospective trials.

## O37 Normal pressure hydrocephalus: questionnaire to detect gait changes and the timeline after spinal tap test (STT)

### Uwe Kehler, Martin Mersch, Max Greiner-Perth, Nico Sinning

#### Department of Neurosurgery, Asklepios Klinik Altona, Hamburg, Germany

##### **Correspondence:** Uwe Kehler - u.kehler@asklepios.com

*Fluids and Barriers of the CNS* 2019, **16(Suppl 3):**O37

**Introduction:** The European NPH Study showed that STT can be used to select patients for but not to exclude patients from shunt surgery. But clinical evaluation was only done once after 3 h. However, the timeline of recovery after STT differs individually. Additionally, recovery may not be detected by gait tests despite subjective improvement. To overcome these shortcomings, we implemented a questionnaire.

**Methods:** On the questionnaire, patients had to log the level of gait changes every hour on day 1, and then once a day for a week after STT. All questionnaires (n = 101) were analyzed for duration and timeline of gait improvement.

**Results:** We had 101 questionnaires from suspected NPH patients (49 males, 52 females, mean age 76.7 years) who had undergone an STT in the last year. Sixty patients declared a substantial, 25 a minimal and 16 no gait improvement. 31 patients experienced improvement despite lacking improved gait tests. 56 patients showed a gait improvement within 30 min and 80 in the first 3 h, lasting for around 6 (0.5–168) h for most patients.

**Conclusions:** After STT, time and duration of improvement vary substantially-so patients should be examined multiple times. Additionally, the high number of only subjective improvement underlines the value of the questionnaire for patients who remain undetected otherwise. However, it remains to be shown, if these also benefit from shunt surgery.

## O38 Diagnostic and prognostic roles of morphological indices in the characterisation of NPH cohorts

### Shereen Soon^1^, Christine Lock^1^, Sumeet Kumar^2^, Janell Kwok^1^, Nicole C. Keong^1,3^, the Alzheimer’s Disease Neuroimaging Initiative^4^

#### ^1^Department of Neurosurgery, National Neuroscience Institute, Singapore; ^2^Department of Neuroradiology, National Neuroscience Institute, Singapore; ^3^Duke-NUS Medical School, Singapore; ^4^The Alzheimer’s Disease Neuroimaging Initiative

##### **Correspondence:** Nicole C. Keong - nchkeong@cantab.net

*Fluids and Barriers of the CNS* 2019, **16(Suppl 3):**O38

**Introduction:** External lumbar CSF drainage (ELD) models shunt responsiveness in patients with NPH. However, brain/ventricular changes predictive of responsiveness remain unclear. We used traditional linear and 3-directional measures to characterize morphological differences between cohorts of NPH responders vs. non-responders, Alzheimer’s Disease (AD) and healthy controls (HC).

**Methods:** 21 participants with NPH underwent ELD and pre- and post-intervention imaging, according to a published NPH protocol. T1-weighted MRI scans from 21 age-matched AD and HC cohorts were acquired from the Alzheimer’s Disease Neuroimaging Initiative (ADNI). We used the NIH platform, 3DSlicer, to derive traditional linear indices, [Evans Index (EI), Bicaudate Index (BCI) and Callosal Angle (CA)], and 3-directional measures [z-Evans Index and Brain per Ventricle Ratio (BVR)].

**Results:** Mean age for NPH study participants was 71.1 ± 6.3 years (18 males, 3 females). There was good intra-rater agreement for all measures (ICC > 0.9). All indices distinguished NPH from AD and HC cohorts (p < 0.001). Within the NPH cohort, nine patients responded to ELD and twelve were non-responders. There were no significant differences in pre-ELD measurements between these groups. Post-drainage, non-responders had a significant decrease in z-Evans Index (p = 0.001) and an increase in BVR at PC (p = 0.024). The increase in BVR was a result of a decrease in the ventricle component of the ratio (p = 0.005).

**Conclusions:** Morphological indices play a role in characterizing NPH vs. non-NPH. Degree of change in ventriculomegaly is not predictive of responsiveness. 3-directional linear measures are superior to traditional indices in differentiating between disease cohorts.

## O39 Utility of DTI profiles across the spectrum of hydrocephalus vs. non-hydrocephalus

### Christine Lock^1^, Janell Kwok^1^, Shereen Soon^1^, Sumeet Kumar^2^, Zofia Czosnyka^3^, Marek Czosnyka^3^, John D. Pickard^3^, Nicole C. Keong^1,4^, the Alzheimer’s Disease Neuroimaging Initiative^5^

#### ^1^Department of Neurosurgery, National Neuroscience Institute, Singapore; ^2^Department of Neuroradiology, National Neuroscience Institute, Singapore; ^3^Neurosurgery Division, Department of Clinical Neurosciences, University of Cambridge, United Kingdom; ^4^Duke-NUS Medical School, Singapore; ^5^The Alzheimer’s Disease Neuroimaging Initiative

##### **Correspondence:** Nicole C. Keong - nchkeong@cantab.net

*Fluids and Barriers of the CNS* 2019, **16(Suppl 3):**O39

**Introduction:** Normal pressure hydrocephalus (NPH) is confounded by similar presentation to other neurodegenerative conditions. We examined the use of diffusion tensor imaging (DTI) profiles to characterize patterns of white matter injury across the NPH spectrum vs. Alzheimer’s disease (AD) and healthy controls.

**Methods:** We performed DTI analysis to generate profiles for three matched datasets of patients vs. controls across the hydrocephalus to non-hydrocephalus spectrum. Our final dataset comprised cohorts with Complex NPH and comorbidities (twelve patients vs. five controls, National Neuroscience Institute), Classic NPH (sixteen patients vs. nine controls, Cambridge) and AD (forty-five AD, forty-seven controls, ADNI). We derived fractional anisotropy (FA) and mean (MD), axial (L1) and radial (L2 and 3) diffusivity measures for four regions of interest (ROIs) in an at-risk model of brain injury—genu (GCC) and body of the corpus callosum (BCC), posterior limb of the internal capsule (PLIC), and inferior fronto-occipital fasciculus/uncinate fasciculus (IFO/UNC).

**Results:** Classic NPH demonstrated increased axial and radial diffusivity at GCC (L1 p = 0.017; L2 and 3 p < 0.001) and BCC (L1 p = 0.029; L2 and 3 p = 0.002) compared to controls. DTI profiles for complex NPH showed similar directional trends (non-significant). DTI profiles for PLIC demonstrated stretch/compression (increased MD, p = 0.004) and L1, p = 0.006) in Classic NPH; in Complex NPH, PLIC displayed more evidence of stretch/oedema. AD displayed general, non-directional deterioration of white matter integrity compared to controls.

**Conclusions:** DTI profiles for AD confirmed deterioration of white matter integrity, whereas both NPH cohorts displayed degrees of stretch/compression indicative of a potential reversibility of injury. Classic and complex NPH had patterns congruent with disease progression.

## O40 Automatic method for measuring Callosal Angle Index (CAI) in differential diagnosis of idiopathic normal pressure hydrocephalus (iNPH)

### Julius Kiilava^1^, Juha Koikkalainen^2^, Jyrki Lötjönen^2^, Ritva Vanninen^1^, Anne M. Koivisto^3^, Hilkka Soininen^3^, Ville Leinonen^4,5^, Valtteri Julkunen^3^

#### ^1^Department of Radiology, University of Eastern Finland and Kuopio University Hospital, Finland; ^2^Combinostics Ltd., Tampere, Finland; ^3^Department of Neurology, University of Eastern Finland and Kuopio University Hospital, Finland; ^4^Department of Neurosurgery, University of Oulu and Oulu University Hospital, Finland; ^5^Department of Neurosurgery, University of Eastern Finland and Kuopio University Hospital, Finland

##### **Correspondence:** Julius Kiilava - kiilava.julius@gmail.com

*Fluids and Barriers of the CNS* 2019, **16(Suppl 3):**O40

**Introduction:** Diagnosis of iNPH is based on clinical symptoms and structural changes in brain imaging including enlargement of lateral ventricles especially to the upwards direction. Widely used method for evaluation of that enlarging is determining the angle between the ventricles seen in coronal section (callosal angle, CA). Manual measurements are though laborious to use and suffer from subjective errors. That is why our goal was to create a fully automatic method for CA measurement.

**Methods:** Patients with iNPH (n = 116) were compared to seven different groups including Alzheimer’s disease (AD, n = 32), frontotemporal dementia (FTD, n = 17), dementia with Lewy bodies (LBD, n = 4), mild cognitive impairment (MCI, n = 9), healthy controls (CN, n = 14), Parkinson’s disease (PD, n = 6) and combined group of patients with vascular dementia or stroke (VaDS, n = 13). MRI image voxel positions were standardized before measurement. After preprocessing, measurements of angle between the lateral ventricles in coronal view were automatically defined in predetermined interval in AP-direction. Total of 50 measurements were performed for each patient in that interval and the results were pooled to make a patient specific CAI-average. Normality of averages was tested, and t-test defined the statistical significance in differences between the groups.

**Results:** CAI was statistically smaller in iNPH (average + standard deviation = 95.4 ± 12.9) compared with all the other groups including AD (121.8 ± 9.9, p = 1.7 * 10^−18^), FTD (121.1 ± 9.0, p = 7.8 * 10^−11^), LBD (118.2 ± 10.3, p = 0.019), MCI (119.5 ± 7.7, p = 2.1 * 10^−6^), CN (120.7 ± 7.5, p = 1.4 * 10^−10^), PD (113.8 ± 16.7, p = 0.042) and VaDS (119.2 ± 11.1, p = 2.3 * 10^−6^).

**Conclusions:** Automatic measurement of CAI provides potential fast and reliable method for differential diagnosis of iNPH.

## O41 Amelioration of CSF flow by creating a pseudomeningocele that enlarges the cisterna magna in pediatric Chiari I patients—uncommon complications

### Arthur R. Kurzbuch^1,2^, Jay Jayamohan^1^, Shailendra Magdum^1^

#### ^1^Department of Pediatric Neurosurgery, Oxford University Hospitals NHS Foundation Trust, John Radcliffe Hospital, UK; ^2^Service de Neurochirurgie, Hôpital du Valais-Centre Hospitalier du Valais Romand (CHVR), Hôpital de Sion, Switzerland

##### **Correspondence:** Arthur R. Kurzbuch - arthurrobert.kurzbuch@hopitalvs.ch

*Fluids and Barriers of the CNS* 2019, **16(Suppl 3):**O41

**Introduction:** In symptomatic pediatric patients with Chiari I malformation we perform in our department decompressive surgery with small sub-occipital craniectomy, C1 laminectomy, durotomy and arachnoid dissection, without dural closure.

**Methods:** 65 consecutive operated pediatric patients with Chiari I malformation were included in a single-center study.

**Results:** The mean age was 10.4 years. 32 patients had syringomyelia and Chiari I malformation. Concomitant scoliosis was present in 19 patients. 52 patients described postoperative improvement, 10 reported no change, 3 noticed clinical worsening. No patient had long term morbidity or mortality. 7 patients needed revision surgery. Complications: 6 patients had CSF leaks, 3 patients aseptic meningitis, and 3 patients subdural hematoma. Uncommon complications were suboccipital intradiploic CSF collections in 3 cases, intraosseous C2 CSF collection in 1 case and de-novo formation of cervical syrinx in 1 case.

**Conclusions:** Chiari I decompressive surgery without dural repair is a viable treatment option. The uncommon complications of iatrogenically induced suboccipital intradiploic and intraosseous CSF collections might be avoided by sealing the exposed suboccipital diploe and cortical breaches of the lamina of C2.

## O42 The iNPH radscale as a diagnostic tool in normal pressure hydrocephalus: sensitivity and specificity

### Karin Kockum^1^, Johan Virhammar^2^, Katrine Riklund^3^, Lars Söderström^4^, Elna-Marie Larsson^5^, Katarina Laurell^1^

#### ^1^Department of Pharmacology and Clinical Neuroscience, Neurology, Östersund, Umeå University, 901 87, Sweden; ^2^Department of Neuroscience, Neurology, Uppsala University Hospital, 751 85, Sweden; ^3^Department of Radiation Sciences, Diagnostic Radiology, Umeå University, 901 87, Sweden; ^4^Unit of Research, Education and Development, Östersund Hospital, 831 31, Sweden; ^5^Department of Surgical Sciences, Radiology, Uppsala University, 751 85, Sweden

##### **Correspondence:** Karin Kockum - karin.kockum@regionjh.se

*Fluids and Barriers of the CNS* 2019, **16(Suppl 3):**O42

**Introduction:** In this retrospective study the idiopathic normal pressure hydrocephalus (iNPH) Radscale scores were assessed in brain computed tomography, with the purpose to evaluate the diagnostic accuracy of the iNPH Radscale.

**Methods:** Seventy-five patients with iNPH, who had undergone ventriculoperitoneal shunt surgery and had been categorized as responders at clinical follow-up after 1 year, were compared with 55 asymptomatic controls (NPH score by Hellström > 90 points). One radiologist assessed the seven radiological features of the iNPH Radscale in computed tomography (CT) of the brain in the patients (preoperatively) and controls.

**Results:** There was a significant difference between the shunted group and control group, with a mean iNPH Radscale score of 10 (IQR 9–11) and 1 (IQR 1–2) respectively, p < 0.001. Receiver operated characteristics analysis yielded an area under the curve of 99.7%, and a cut off level of iNPH Radscale score of 4 corresponded to a sensitivity of 100% and a specificity of 91%, with an overall accuracy of 96.2%.

**Conclusions:** The iNPH Radscale can accurately separate shunt responsive iNPH patients from controls. This could be useful in excluding patients from the disease.

## O43 Evaluation of neurodegenerative and inflammatory CSF biomarkers in idiopathic normal pressure hydrocephalus

### Alexandria E. Lewis, Jacqueline A. Darrow, Kristina Khingelova, Seema Gulyani, Abhay R. Moghekar

#### Department of Neurology, Johns Hopkins University, Baltimore, Maryland, 21287, USA

##### **Correspondence:** Abhay Moghekar - am@jhmi.edu

*Fluids and Barriers of the CNS* 2019, **16(Suppl 3):**O43

**Introduction:** The role of biomarkers in the selection of idiopathic normal pressure hydrocephalus (iNPH) patients for shunt surgery has been studied in small populations. The aim of the study was to evaluate potential differences in neurodegenerative (Neurofilament Light-NF-L) and inflammatory (Leucine-rich alpha-2-glycoprotein-LRG) biomarkers between patients selected for shunt surgery based on their response to a CSF diversion procedure and determine any correlation with age, cognition, and gait.

**Methods:** CSF was obtained from iNPH patients after a baseline assessment of Montreal Cognitive Assessment (MOCA), Timed Up and Go (TUG), and 10 M walk testing prior to their procedure. Patients were deemed to be responders and referred for shunt surgery based on iNPH guidelines. CSF was analyzed using a Quanterix SIMOA NF-L immunoassay and a traditional LRG sandwich ELISA on the Filter Max F3 platform.

**Results:** In the iNPH cohort the mean age was 73 ± 10 years with 167 males and 98 females. In subjects who were deemed responders to CSF diversion the levels of NFL were 2051.62 ± 1758.67 vs 2732.16 ± 4209.78 in non-responders (p = 0.1882) and levels of LRG were 682.61 ± 492.80 in responders and 587.49 ± 423.96 in non-responders (p = 0.1231). There was no correlation between these biomarkers and cognition and gait measures in our sample.

**Conclusions**: In this large iNPH population we did not observe differences in neurodegenerative and inflammatory biomarkers between CSF diversion responders and non-responders and hence should not be used to screen patients for shunt surgery. Future studies will be necessary to determine if these biomarkers predict long-term shunt response.

## O44 Application of grooved pegboard test in cerebrospinal fluid tap test of patients with idiopathic normal pressure hydrocephalus

### Caiyan Liu^1^, Liling Dong^1^, Chenhui Mao^1^, Jie Li^1^, Xinying Huang^1^, Junji Wei^2^, Bo Hou^3^, Feng Feng^3^, Lingying Cui^1^,Jing Gao^1^

#### ^1^Department of Neurology, Peking Union Medical College Hospital, Chinese Academy of Medical Sciences, Beijing, China; ^2^Department of Neurosurgery, Peking Union Medical College Hospital, Chinese Academy of Medical Sciences, Beijing, China; ^3^Department of Neurosurgery and Radiology, Peking Union Medical College Hospital, Chinese Academy of Medical Sciences, Beijing, China

##### **Correspondence:** Jing Gao - liucy-pumch@163.com

*Fluids and Barriers of the CNS* 2019, **16(Suppl 3):**O44

**Introduction:** Motor impairment in NPH can extend beyond gait to include deficits in upper extremity functions and psychomotor speed. The upper extremity function evaluation will be helpful for the NPH patients who are unable to ambulate (e.g. patient is wheelchair bound) and may not be able to comply with the gait evaluation. Our study aimed to explore the role of grooved pegboard test in evaluating cerebrospinal fluid tap test in patients with idiopathic normal pressure hydrocephalus.

**Methods:** Forty-three possible iNPH patients were enrolled from 2013 to 2017. All patients underwent detailed neuropsychological and walking assessments, CSF tap test, as well as head magnetic resonance imaging. The correlation between grooved pegboard test performance and other clinical assessment were analyzed. In DTI analysis of white matter, the FA and MD values of 19 regions of interest were measured by ROI method. The correlation between grooved pegboard test performance and the white matter lesions were also analyzed.

**Results:** The results of the grooved pegboard test were significantly correlated with patients’ walking ability, cognitive function, function score (P < 0.05). The time for the left grooved pegboard test was significantly correlated with FA value of right periventricular lesions (P = 0.017).

**Conclusions:** The performance of the grooved pegboard test was related to lower extremity motor ability and cognitive function. It can be used as an alternative evaluation tool for patients who are unable to ambulate and may not be able to comply with the gait evaluation. This project was supported by grant from CAMS 2016-12M-1-004, National NSFC81550021, 2016YFC1306300, grant XDPB10.

## O45 Correlation between white matter lesions and clinical features of patients with idiopathic normal pressure hydrocephalus in CSF tap test

### Caiyan Liu^1^, Liling Dong^1^, Chenhui Mao^1^, Jie Li^1^, Xinying Huang^1^, Junji Wei^2^, Bo Hou^3^, Feng Feng^3^, Liying Cui^1^, Jing Gao^1^

#### ^1^Department of Neurology, Peking Union Medical College Hospital, Chinese Academy of Medical Sciences, Beijing, China; ^2^Department of Neurosurgery, Peking Union Medical College Hospital, Chinese Academy of Medical Sciences, Beijing, China; ^3^Department of Neurosurgery and Radiology, Peking Union Medical College Hospital, Chinese Academy of Medical Sciences, Beijing, China

##### **Correspondence:** Jing Gao - liucy-pumch@163.com

*Fluids and Barriers of the CNS* 2019, **16(Suppl 3):**O45

**Introduction:** It is still controversial about white matter lesions and the cerebrospinal drainage outcome in patients with idiopathic normal pressure hydrocephalus (iNPH). Our study aimed to explore the relationship between white matter lesions and clinical features and response of CSF tap test in patients with iNPH.

**Methods:** Forty-three possible iNPH patients were enrolled from 2013 to 2017. All patients underwent detailed neuropsychological and walking assessments, CSF tap test, as well as head magnetic resonance imaging. The Fazekas score of white matter lesions, the differences of the FA and MD values of 19 regions of interest area by means of DTI were compared between CSF tap test positive and negative response groups. And the correlation between DTI parameters and clinical characteristics was analyzed.

**Results:** Compared with the negative group, the positive group tended to have higher Fazekas score of periventricular white matter (beta = 0.895, P = 0.068). In DTI analysis, the positive group had the significantly higher ADC value in the left ventricle posterior area lesions (P = 0.003), tended to have the higher FA value of the lesions in the right ventricle anterior area and the ADC value of the right ventricle posterior area lesions (P = 0.058, P = 0.058). The FA value of right ventricular anterior area was significantly correlated with motor function, cognitive and functional score and iNPHGS score.

**Conclusions:** Periventricular white matter lesions in patients with idiopathic normal pressure hydrocephalus are significantly correlated with their clinical features, which may be one of the pathogenesis mechanisms and the target for improving symptoms after drainage surgery.

## O46 Diagnosis of idiopathic normal pressure hydrocephalus using protein tyrosine phosphatase receptor type Q concentration in the cerebrospinal fluid

### Nakajima Madoka^1^, Tuomas Rauramaa^2^, Petra M. Mäkinen^3^, Mikko Hiltunen^3^, Sanna-Kaisa Herukka^4,5^, Merja Kokki^6^, Henna-Kaisa Jyrkkänen^7,8^, Nils Danner^7,8^, Antti Jukkari^7,8^, Anne M. Koivisto^4,5^, Juha E. Jääskeläinen^7,8^, Masakazu Miyajima^1^, Ikuko Ogino^1^, Akiko Furuta^9^, Chihiro Akiba^1^, Kaito Kawamura^1^, Chihiro Kamohara^1^, Hidenori Sugano^1^, Yuichi Tange^1^, Kostadin Karagiozov^1^, Ville Leinonen^10^, Hajime Arai^1^

#### ^1^Department of Neurosurgery, Faculty of Medicine, Juntendo University, Tokyo, Japan; ^2^Institute of Clinical Medicine-Pathology, University of Eastern Finland and Department of Pathology, Kuopio University Hospital, Finland; ^3^Institute of Biomedicine, University of Eastern Finland, Kuopio, Finland; ^4^Institute of Clinical Medicine-Neurology, University of Eastern Finland, Kuopio, Finland; ^5^Neurocenter, Neurology, Kuopio University Hospital, Finland; ^6^Anesthesia and Operative Services, Kuopio University Hospital, Finland; ^7^Institute of Clinical Medicine-Neurosurgery, University of Eastern Finland; ^8^Neurocenter, Neurosurgery, Kuopio University Hospital, Finland; ^9^Department of Psychiatry and Behavioral Science, Juntendo University Faculty of Medicine, Tokyo, Japan; ^10^Unit of Clinical Neuroscience, Neurosurgery, University of Oulu and Medical Research Center, Oulu University Hospital, Oulu, Finland

##### **Correspondence:** Nakajima Madoka - madoka66@juntendo.ac.jp

*Fluids and Barriers of the CNS* 2019, **16(Suppl 3):**O46

**Introduction:** We investigated the possibility of using protein tyrosine phosphatase receptor type Q (PTPRQ) for auxiliary diagnosis of idiopathic normal pressure hydrocephalus (iNPH), and carried out the first intracerebral analysis of PTPRQ expression in autopsied brains of patients with iNPH.

**Methods:** We analyzed the feasibility of using PTPRQ concentrations in the cerebrospinal fluid (CSF) for auxiliary diagnosis of iNPH in the Finnish (n = 30) and Japanese (n = 30) population. PTPRQ concentrations in iNPH patients and healthy elderly subjects with normal cognition (NC, n = 40) were compared. PTPRQ expression levels were measured in autopsied brains of iNPH patients and NC subjects.

**Results:** PTPRQ concentration was increased by iNPH; the concentration was higher in the Finnish-iNPH (mean 762 [SD 570] pg/mL) and the Japanese-iNPH (712 [832]) groups than in the NC group (351 [100]) (p < 0.001 and p = 0.018, respectively). In a combined Finnish and Japanese iNPH group, using a PTPRQ cutoff of 370 pg/mL, iNPH was detected with a sensitivity, specificity, and area under receiver operating characteristic curve of 75%, 65%, and 0.771, respectively.

**Conclusions:** Measurement of PTPRQ in the CSF by ELISA showed levels approximately 2 times higher in patients with iNPH than in healthy elderly subjects, regardless of the racial group, confirming the validity of this assay for auxiliary diagnosis. The absence of a relationship between PTPRQ and p-Tau, t-Tau, and Aβ42 markers of AD pathology, and the high levels of PTPRQ in patients with iNPH have an important diagnostic merit.

## O47 Difference of water turnover in brain tissue and CSF spaces between normal volunteers and patients with idiopathic NPH: dynamic PET study using [^15^O]H_2_O

### Mitsuhito Mase^1^, Emi Hayashi^2^, Shin Hibino^2^, Yoshihiro Ito^2^, Akihiko Iida^2^, Toshiaki Miyati^3^, Etsuro Mori^4^

#### ^1^Department of Neurosurgery, Nagoya City University Graduate School of Medical Sciences, Japan; ^2^Department of Radiology, Nagoya City Rehabilitation Center, Japan; ^3^Faculty of Health Science, Institute of Medical, Pharmaceutical and Health Sciences, Kanazawa University, Japan; ^4^Department of Behavioral Neurology and Neuropsychiatry, Osaka University United Graduate School of Child Development, Japan

##### **Correspondence:** Mitsuhito Mase - mitmase@med.nagoya-cu.ac.jp

*Fluids and Barriers of the CNS* 2019, **16(Suppl 3):**O47

**Introduction:** In order to clarify changes of turnover of water molecules in CSF in idiopathic NPH (iNPH), dynamic PET was performed using radio labeled H_2_O.

**Methods:** Normal volunteers (n = 10) and patients with definite iNPH (n = 5) were included. Dynamic PET data were obtained for 15 min after intravenous [^15^O]H_2_O (500 MBq). Voxels of interest were set in internal carotid artery (ICA), superior sagittal sinus (SSS), cortical gray matter (GM), white matter (WM), basal ganglia (BG), lateral ventricle (LV), Sylvian fissure (FS), and prepontine cistern (PPC). Time and relative radio activity (RAA) curves of each VOI were analyzed.

**Results:** In the control group, the peak radio activities of GM, WM and BG were at 22.5, 50.0 and 22.5 s after the peak in ICA, respectively. Activities in whole brain structures decreased gradually. On the contrary, activities of LV, FS and PPC increased gradually until the end of measurement. In the iNPH group, RRA of BG was significantly lower than controls. RRA curves of GM and WM were decreasing and also getting closer, each other in late phase. This means diffusion of water molecules in brain resulting in equal distribution in time. Compared with the controls, it took significantly longer until the equal distribution in brain in iNPH. RRA of LV, FS and PPC in iNPH tended to be lower compared to controls. After L-P shunt, these delays tended to be normalize.

**Conclusions:** Water turnover in brain and CSF is reduced or delayed in iNPH compared to normal, which is normalized after shunt surgery.

## O48 Perforation holes in ventricular catheters. Is less more? Chapter three. Acute hydrocephalus and intraventricular haemorrhage

### Angelo L. Maset^1^, Jakeline F. S. Santos^1^, Felipe Muniz^1^, Ruy Monteiro^2^, Gustavo Botelho^3^, Dionei F. Morais^1^

#### ^1^Department of Neurosurgery, Neurosurgical Clinical Research, Fundaçāo Faculdade de Medicina de Sāo José do Rio Preto, Brazil; ^2^Department of Neurosurgery, Hospital Municipal Miguel Couto, Rio de Janeiro, Brazil; ^3^Pediatric Neurosurgeon

##### **Correspondence:** Angelo L. Maset - maset@terra.com.br

*Fluids and Barriers of the CNS* 2019, **16(Suppl 3):**O48

**Introduction:** Much has been published and attempted to improve EVD survival and clearance of haemorrhagic CSF from the ventricles in intraventricular haemorrhage. They do represent a very particular type of blockage, causing an increased morbidity. We applied and followed two different methodologies of external ventricular drainage by using two different concepts of EVD catheters and compared the effectiveness of each method in relation to its impact in the number of hospitalization days, intensive care days (I.C.U.), hydrocephalus occurrence and patient’s general outcome.

**Methods:** Electronic files of 30 patients with intraventricular haemorrhage between March 01, 2014 and April 30 2015 were analysed. They were divided in two groups: group H (15 patients who used catheters LCR600H and group C (15 patients who used conventional catheter LCR600A). The only difference was the design of the catheter.

**Results:** There was a significant difference on number of days that patients remained at the (I.C.U.) in favour of group H compared to group C (p < 0.01). Also, group H ventricular catheters were withdrawn earlier than group C catheters (p < 0.01).

**Conclusions:** Results allows us to conclude that the LCR600H catheter was able to show some advantages for influencing the risks exposure and permanence in the ICU compared to conventional catheters. Current results warrants a more detailed and multicentric study to evaluated social and financial impact on the Brazilian Health care.

## O49 Brain tissue stiffness decreases in children with hydrocephalus

### Mark E. Wagshul^1^, James P. McAllister^2^, I. Rick Abbott^1^, David D. Limbrick, Jr^2,3^, Diego M. Morales^1^, James T. Goodrich^1^, Richard Nagel^4^

#### ^1^Department of Radiology and the Gruss Magnetic Resonance Research Center, Albert Einstein College of Medicine, Bronx, New York, 10461, USA; ^2^Department of Neurosurgery, Division of Pediatric Neurosurgery, Washington University School of Medicine, St. Louis, Missouri, 63110, USA; ^3^Department of Pediatrics, Washington University School of Medicine and St. Louis Children’s Hospital, Missouri, 63110, USA; ^4^Department of Radiology, Washington University School of Medicine and the Mallinckrodt Institute of Radiology, St. Louis, Missouri, 63110, USA

##### **Correspondence:** James Mcallister - pat.mcallister@wustl.edu

*Fluids and Barriers of the CNS* 2019, **16(Suppl 3):**O49

**Introduction:** Brain compliance in hydrocephalus remains a controversial issue; i.e. stiffness reportedly increases in NPH and decreased stiffness may contribute to ETV failure. We used Magnetic Resonance Elastography (MRE), a new tool to measure brain stiffness non-invasively, to determine if stiffness is altered in pediatric hydrocephalus.

**Methods:** From 2 centers, 40 shunt-dependent patients (age 0.6–39 year, median 18.0) who developed hydrocephalus as infants were compared to 27 healthy age-matched controls (age 6–46 year, median 16.7). MRE was performed by inducing a 30 Hz vibration transmitted through the skull. Tissue elastance (G*, inverse of stiffness) averaged separately across white and grey matter masks and within lobar regions was calculated through Algebraic Helmholtz Inversion. The Headache Disability Index (HDI) and Hydrocephalus Outcome Questionnaire (HOQ) were collected in all Einstein patients.

**Results:** In periventricular white matter, brain tissue stiffness was reduced significantly (p < 0.005) in patients compared to controls (G* = 1.75 ± 0.28 kPa vs. 1.97 ± 0.22 kPa). Occipital grey matter stiffness correlated negatively with ventricular size (R^2^ = 0.23, p < 0.001). There was a weak positive correlation between occipital grey matter elastance and HOQ (R^2^ = 0.16, p < 0.05), and a negative trend correlation between occipital grey matter stiffness and HDI (R^2^ = 0.14, p = 0.056). One patient scanned 1 day prior to shunt revision, and 11 months following revision, exhibited increased stiffness (i.e. toward controls) in all lobes except the occipital lobe where stiffness decreased.

**Conclusions:** Brain stiffness was reduced in hydrocephalus patients, suggesting impaired biomechanical integrity of brain tissue.

## O50 Do co-morbidities influence shunt outcomes in idiopathic intracranial hypertension?

### Saniya Mediratta, Jonathan P. Funnell, Linda D’Antona, Lewis Thorne, Laurence D. Watkins, Ahmed K. Toma

#### Victor Horsley Department of Neurosurgery, The National Hospital for Neurology and Neurosurgery, London, UK

##### **Correspondence:** Saniya Mediratta - saniya.mediratta@gmail.com

*Fluids and Barriers of the CNS* 2019, **16(Suppl 3):**O50

**Introduction:** Idiopathic intracranial hypertension (IIH) is often associated with obesity, chronic pain, and mental health conditions. This study aims to investigate the prevalence of these co-morbidities and their impact on surgical management of IIH.

**Methods:** Single-centre retrospective study of IIH patients who received a new ventriculoperitoneal, ventriculoatrial or lumboperitoneal shunt between January 2015 and March 2019. Operation notes and clinic letters were used to record comorbidities and the number of shunt revisions and valve adjustments each patient had.

**Results:** Forty-five patients (39F:6M, aged 35.5 ± 11). 37.8% had a diagnosis of at least one chronic pain syndrome (CPS) including chronic fatigue syndrome, fibromyalgia and chronic back pain. Mental health conditions, particularly depression, were common (40%). 47% of patients with CPS had at least one subsequent shunt revision compared to 25% of patients without CPS. Shunt revisions were required in 38.8% of patients with a mental health condition and 29.6% of patients without this comorbidity. The mean number of valve adjustments was 1.65 and 1.68 in CPS and no CPS groups respectively. Mean valve adjustments was 2.11 for patients with a mental health condition and 1.33 for patients without.

**Conclusions:** Patients with CPS and mental health conditions may have a higher rate of shunt revisions, and patients with mental health conditions may undergo more valve adjustments. These co-morbidities could form an important part of IIH surgical planning. Further work to identify physical or mental co-morbidities as individual risk factors for multiple interventions and a greater understanding of the relationship between IIH and these co-morbidities would be useful.

## O51 Elucidation of the mechanism of hydrocephalus in H-TX rat

### Masakazu Miyajima^1,2^, Madoka Nakajima^2^, Ikuko Ogino^2^, Chihiro Akiba^1,2^, Kaito Kawamura^2^, Xu Hanbing^2^, Chihiro Kamohara^2^, Wei Meng^2^, Norihiro Tada^3^, Hajime Arai^2^

#### ^1^Department of Neurosurgery, Juntendo Tokyo Koto Geriatric Medical Center, Japan; ^2^Department of Neurosurgery, Graduate School of Medicine, Juntendo University, Tokyo, Japan; ^3^Laboratory of Biomedical Research Resources, Center for Biomedical Research Resources, Graduate School of Medicine, Juntendo University, Tokyo, Japan

##### **Correspondence:** Masakazu Miyajima - mmasaka@juntendo.ac.jp

*Fluids and Barriers of the CNS* 2019, **16(Suppl 3):**O51

**Introduction:** Aqueductal stenosis occurs with a high frequency of 0.1% to 0.3% of births and it is known to be the main cause of congenital hydrocephalus. The development and functional impairment of the subcommissural organ and ependymal cells have gained attention due to their involvement in the development of hydrocephalus. In H-Tx rats, immunoreactivities of the glycoprotein of the subcommissural organ and ependymal cells of the midbrain decrease before ventricular enlargement. However, the genetic abnormality causing congenital hydrocephalus in H-Tx rats remains unknown. In this study, we performed a copy number analysis to investigate the candidate genes responsible for inducing hydrocephalus in H-Tx rats.

**Methods:** DNA was extracted from the brains of H-Tx rats with and without hydrocephalus. The CGH array was performed and analysis was conducted using software. Expression levels of the identified genes and proteins were verified by employing qPCR, immunohistological staining and western blotting using another cohort. Additionally, the CRISPR/Cas9-mediated mutagenesis was used to generate knockout mice with the identified gene.

**Results:** The histidine-rich glycoprotein (Hrg) and protein-tyrosine phosphatase non-receptor type 20B (Ptpn20) were identified as candidate genes and their deletions were observed in the H-Tx rats with hydrocephalus. Ptpn20 knockout mice showed mild ventricular dilatation.

**Conclusions:** Hrg may be one of the glycoproteins constituting the Reissner’s fiber. Ptpn20 is a protein that is expressed in the ependymal cells and involved in cell polarity. These results suggest that abnormalities in the two genes may be associated with the development of hydrocephalus.

## O52 Altered regional cerebral glucose metabolism in preclinical stages of iNPH

### Koichi Miyazaki^1^, Kohei Hanaoka^2^, Hayato Kaida^1^, Kazunari Ishii^1,2^

#### ^1^Department of Radiology, Kindai University Faculty of Medicine, Osakasayama, Osaka, Japan; ^2^Institute of Advanced Clinical Medicine, Kindai University, Osakasayama, Osaka, Japan

##### **Correspondence:** Koichi Miyazaki - rpmy69126@nike.eonet.ne.jp

*Fluids and Barriers of the CNS* 2019, **16(Suppl 3):**O52

**Introduction:** Several reports have shown that cerebral glucose metabolism is reduced in patients with iNPH. However, the time at which the metabolic change occurs is still unknown. To elucidate the progress of cerebral glucose metabolism change, we evaluated FDG-PET images in patients with preclinical and developing iNPH.

**Methods:** We conducted a cross-sectional study in > 2000 elderly patients who had underwent whole body FDG-PET/CT scanning, and registered cases with hydrocephalus. 96 cases with hydrocephalus were found and classified into three groups: preclinical morphologic features of DESH (PMD), AVIM and iNPH. Cases with PMD were asymptomatic and have uncomplete DESH findings. Cases with AVIM were also asymptomatic but have complete DESH findings. We have been considering and reporting that iNPH develops in the order of PMD, AVIM, iNPH. We measured mean regional standardized uptake value ratio (SUVR) on FDG-PET images among three groups, and compared them with backgrounds matched controls for each regions: frontal lobes, medial parietal cortices, temporal lobes, striata, and thalami.

**Results:** The frontal SUVRs showed significantly decreased in AVIM and iNPH. The medial parietal SUVRs showed significantly increased in PMD and AVIM. The temporal lobes showed significantly decreased SUVRs in PMD and AVIM. In the striata, SUVR was decreased only in the iNPH groups. Thalamic metabolism had only a tendency to show decreasing SUVR following iNPH development.

**Conclusions:** Altered cerebral glucose metabolism in iNPH was observed even in the preclinical stages. The change tended to appear earlier in the cortices than in the basal ganglia.

## O53 Initial clinical experience with a novel ventricular catheter and flushing system for hydrocephalus patients prone to proximal catheter occlusions

### Michael Muhonen, Joffre Olaya

#### Department of Neurosurgery, Children’s Hospital Orange County (CHOC), Orange, California, 92868, USA

##### **Correspondence:** Michael Muhonen - mmuhonen@choc.org

*Fluids and Barriers of the CNS* 2019, **16(Suppl 3):**O53

**Introduction:** Ventricular Catheter (VC) obstruction is the most common cause of shunt failure. This abstract reports our initial clinical experience with the FDA cleared ReFlow Ventricular System (Anuncia, Inc, Lowell, MA). When activated the novel flusher generates a non-invasive retrograde flush to unblock the inlet holes of the VC or open its unique relief membrane to restore CSF flow.

**Methods:** We report our experience in two patients with congenital hydrocephalus associated with spina bifida: a 15 y/o female who presented with enlarged ventricles and ventricular catheter occlusion, and a 6 y/o male who presented with headaches and irritability after multiple previous shunt revisions. In both, complete shunt revisions were performed. The ReFlow device was implanted proximal to a standard adjustable shunt valve (Integra Codman) and peritoneal catheter, primed, and pumped.

**Results:** The implantation and flusher pumping procedure was well tolerated in both patients. CSF was observed to be flowing through the system. Both systems pumped easily and refilled when tested post-operatively. Workflow and procedure time were not significantly impacted. Patients remain absent of any symptoms to suggest shunt failure.

**Conclusions:** Preliminary observations indicate the implantation and use of the Reflow is a safe and may offer an effective, non-invasive method to restore flow in occluded proximal catheters to prevent revision surgery. Further studies are needed to evaluate its suitability for patients with a history of shunt failure, who may benefit from prophylactic flushing to proactively prevent occlusion. Miniaturization of the flusher component for infants and younger patients is recommended.

* *Informed consent to publish has been obtained from the parents of the two patients*

## O54 Cerebrospinal fluid diversion in idiopathic intracranial hypertension: should surgery come first?

### Declan G. Siedler, Asim Mujic

#### Department of Neurosurgery, Royal Hobart Hospital, TAS 7000, Australia

##### **Correspondence:** Declan Siedler - declan.siedler@ths.tas.gov.au

*Fluids and Barriers of the CNS* 2019, **16(Suppl 3):**O54

**Introduction:** Idiopathic intracranial hypertension (IIH) is a debilitating syndrome characterized by raised intracranial pressure (ICP) following exclusion of all other causes. Although managed pharmacologically in the first instance, medication side effects often result in poor treatment compliance. If not definitively managed, patients are placed at risk of worsening vision loss or blindness. Cerebrospinal fluid (CSF) diversion procedures are often considered in refractory disease, however early utilization may result in less patient morbidity.

**Methods:** We considered all patients who underwent insertion or revision of a CSF diversion procedure for IIH at the Royal Hobart Hospital between 2008 and 2019. Data was collected from the hospital’s digital medical records. Patient demographics, smoking status and comorbidities were considered. Factors such as presenting clinical symptoms and signs were compared with postoperative findings.

**Results:** A total of 20 patients underwent 26 CSF diversions, either an index or revision procedure, for IIH. All patients were female, with a mean age of 27.6 years (SD ± 8.7). Following CSF diversion, we observed a 70% reduction in reported headaches and visual symptoms. Of the 15 patients who underwent visual acuity testing pre- and post-surgery, nine demonstrated an improvement with no deterioration occurring as a consequence of the procedure.

**Conclusions:** CSF diversion procedures are a safe and effective option in the management of IIH. It has the capacity to decrease patient morbidity acutely. We advocate for early CSF diversion in IIH, although recognize further research needs to be undertaken.

## O55 Case series of ventriculo-atrial shunts in adults: a single-centre experience

### Sophie R. Mullins, Claudia L. Craven, Simon Thompson, Linda D’Antona, Lewis Thorne, Ahmed K. Toma, Laurence D. Watkins

#### Victor Horsley Department of Neurosurgery, National Hospital for Neurology and Neurosurgery, London, UK

##### **Correspondence:** Sophie R. Mullins - sophie.mullins@nhs.net

*Fluids and Barriers of the CNS* 2019, **16(Suppl 3):**O55

**Introduction:** Ventriculoatrial (VA) shunts are an alternative way of shunting cerebrospinal fluid (CSF) when the more common distal site of the peritoneal cavity is contraindicated. This study aims to look at a single centre’s experience with VA shunts in idiopathic intracranial hypertension (IIH).

**Methods:** Retrospective review of electronic records over a 9 year time period at our centre. Exclusion criteria were: duplication of same shunt insertion, no VA shunt insertion, paediatric patients and indication other than IIH. Notes were reviewed for demographics, survival (defined by time prior to revision) and reasons for revision.

**Results:** Seven VA shunt procedures were identified. All shunts were secondary procedures; 2 revisions from ventriculoperitoneal, 2 ventriculopleural, 1 lumbo-pleural, and 2 from previous VA. At time of completion of study median survival was 15 months (range 1 day–37 months) with 5 (71.4%) shunts remaining in situ at the end of the study. Revisions were required due to acute intracranial bleed (1 case)—revised at day 1, and thrombus at distal site (1 case)—revised at day 57. Both shunts were later reinserted. There were no cases of shunt nephritis reported in this cases series. From latest clinic letters 4 patients had their treatment optimised with this procedure, with one not yet seen for follow-up.

**Conclusions:** Ventriculoatrial shunts are a safe and efficacious option for second-line procedure in IIH. In this series only 1 shunt was revised for a VA shunt specific complication.

## O56 Intraoperative shuntography reduced catheter migration in lumbo-peritoneal shunt surgery

### Hisayuki Murai^1^, Toshimasa Shin^1,2^

#### ^1^Department of Neurosurgery, Saisekikai-Narashino Hospital, Narashino, Chiba, 275-8580, Japan; ^2^Chiba University, 260-0856, Japan

##### **Correspondence:** Hisayuki Murai - murai@chiba-saiseikai.com

*Fluids and Barriers of the CNS* 2019, **16(Suppl 3):**O56

**Introduction:** In Japan, iNPH patients prefer lumbo-peritoneal (LP) shunt more than ventriculo-peritoneal shunt recently. LP shunt does not need brain surgery and is less invasive. However, LP shunt has some pitfalls. Post-operative abdominal catheter migration was reported in Sinphoni 2 study and then abdominal catheter has been sutured to the abdominal fascia in our institute. We experienced another complication of lumber catheter migration during surgery. Intraoperative CSF out-flow was good, and we didn’t notice the epidural migration during the surgery. So, we started intraoperative shuntography.

**Methods:** From April 2015 to September 2017, 32 iNPH cases were lumbo-peritoneally shunted with intraoperative shuntography. After test tap with 21-gauge lumbar needle, Tuohy needle was inserted. During the lumbar catheter insertion through the Tuohy needle, 1 to 3 ml of the water-soluble nonionic iodinated contrast agent was slowly injected to visualize the tube form and surrounding structure. When the lumber catheter was in wave or hair pin form, or in the epidural cavity, tube insertion was re-performed. Also, when the abdominal catheter was suspected to be malpositioned in one case, abdominal shuntography was also performed.

**Results:** In 32 cases, there was no complication associated with shuntography. During the observation period, one cerebellar hemorrhage, one cerebral infarction, one prolonged intracranial hypotension which lasted over a week and one catheter disruption was found. The lumbar catheter was in the epidural cavity in two cases and the abdominal catheter was outside the peritoneal cavity in one case. Those were successfully replaced.

**Conclusions:** Intraoperative shuntography is safe and it reduced catheter migration in lumbo-peritoneal shunts.

## O57 Prevalence of cervical stenosis and myelopathy in patients with idiopathic normal pressure hydrocephalus

### Ryan M. Naylor^1^, Karina A. Lenartowicz^2^, Jonathan Graff-Radford^3^, David T. Jones^3^, Jeremy Cutsforth-Gregory^3^, Benjamin D. Elder^1,4,5^

#### ^1^Department of Neurological Surgery, Mayo Clinic, Rochester, Minnesota, 55905, USA; ^2^Mayo Clinic Alix School of Medicine, Mayo Clinic, Rochester, Minnesota, 55905, USA; ^3^Department of Neurology, Mayo Clinic, Rochester, Minnesota, 55905, USA; ^4^Department of Orthopedic Surgery, Mayo Clinic, Rochester, Minnesota, 55905, USA; ^5^Department of Biomedical Engineering, Mayo Clinic, Rochester, Minnesota, 55905, USA

##### **Correspondence:** Benjamin D. Elder - elder.benjamin@mayo.edu

*Fluids and Barriers of the CNS* 2019, **16(Suppl 3):**O57

**Introduction:** Both idiopathic normal pressure hydrocephalus (iNPH) and cervical myelopathy may result in progressive gait impairment. It is possible that some of the patients who do not respond to shunting despite a positive tap test may have gait dysfunction from cervical myelopathy. The objective of this study was to determine the prevalence of cervical stenosis with or without myelopathy in patients with iNPH.

**Methods:** We screened a consecutive series of patients who underwent shunt placement for iNPH for comorbid cervical stenosis. Clinical manifestations of iNPH and cervical myelopathy, grade of cervical stenosis based on previously published criteria, cervical spine surgical intervention, timing of intervention, and outcomes were recorded.

**Results:** Forty-two patients with iNPH were included for analysis. Slightly more patients were male (65%), with a mean age of 75 years (SD 7 years) for the entire cohort. All patients presented with gait disturbances and underwent cervical spine MRI. 30/42 (71%) had at least cervical stenosis, while 7/42 (17%) had significant (grade 2–3) cervical stenosis with myelopathy requiring surgical decompression. All patients with grade 2–3 cervical stenosis and symptoms of cervical myelopathy in addition to iNPH underwent cervical decompression surgery.

**Conclusions:** Clinically significant cervical stenosis is highly prevalent in patients with iNPH, though this finding requires validation in a larger population. Based on these results, cervical imaging should be considered preoperatively or in patients whose gait does not improve after shunt placement.

## O58 Spinal deformity rates in patients with idiopathic normal pressure hydrocephalus

### Ryan M. Naylor^1^, Jonathan Graff-Radford^2^, David T. Jones^2^, Jeremy Cutsforth-Gregory^2^, Jeremy Fogelson^1,3^, Benjamin D. Elder^1,3,4^

#### ^1^Department of Neurological Surgery, Mayo Clinic, Rochester, Minnesota, 55905, USA; ^2^Department of Neurology, Mayo Clinic, Rochester, Minnesota, 55905, USA; ^3^Department of Orthopedic Surgery, Mayo Clinic, Rochester, Minnesota, 55905, USA; ^4^Department of Biomedical Engineering, Mayo Clinic, Rochester, Minnesota, 55905, USA

##### **Correspondence:** Benjamin D. Elder - elder.benjamin@mayo.edu

*Fluids and Barriers of the CNS* 2019, **16(Suppl 3):**O58

**Introduction:** Postural instability, often with a forward leaning posture, is a common feature in idiopathic normal pressure hydrocephalus (iNPH). However, the spinopelvic alignment and presence of concomitant spinal sagittal plane deformity has not been well studied in the iNPH population. The objective of this study was to measure the baseline spinopelvic parameters and determine the prevalence of sagittal plane spinal deformity in patients with iNPH.

**Methods:** We reviewed a series of patients who underwent VP shunting for the treatment of iNPH and who also had standing scoliosis x-rays. We evaluated for comorbid spinal deformity based on the SRS-Schwab adult spinal deformity classification system by assessing pelvic incidence minus lumbar lordosis (PI-LL), pelvic tilt (PT), and sagittal vertical axis (SVA).

**Results:** Seventeen patients with iNPH were included for analysis. Six patients (35%) met criteria for having marked spinal deformity by at least one radiographic parameter: 5 (29%) had greater than 20° PI-LL mismatch, 3 (18%) had > 9.5 cm SVA, and 1 (6%) had PT exceeding 30°. Additionally, the degree of thoracic kyphosis exceeded that of lumbar lordosis in 9 patients (53%).

**Conclusions:** Sagittal plane spinal deformities may be common in iNPH patients and may contribute to postural instability in this patient population. Moreover, since thoracic kyphosis exceeded lumbar lordosis in more than half of the patients, the forward leaning postural instability in iNPH patients may be due to increased thoracic kyphosis. These findings warrant further investigation along with a determination if there is any change in the parameters following shunt placement.

## O59 Extended lumbar drainage in idiopathic normal pressure hydrocephalus: a systematic review of diagnostic test accuracy

### Adam C. Nunn^1^, Hayley E. Jones^2^, Cezar O. Morosanu^1^, William G. B. Singleton^1^, Michael A. Williams^3^, Sean Jeremy Nagel^3^, Mark G. Luciano^3^, Thomas J. Zwimpfer^3^, Richard Holubkov^3^, Jeffrey H. Wisoff^3^, Guy M. McKhann, II^3^, Mark G. Hamilton^3^, Richard J. Edwards^1,3^

#### ^1^Department of Neurosurgery, North Bristol NHS Trust, BS10 5NB, UK; ^2^Population Health Sciences, Bristol Medical School, University of Bristol, BS8 1TH, UK; ^3^Adult Hydrocephalus Clinical Research Network, Hydrocephalus Association, Bethesda, USA

##### **Correspondence:** Adam Nunn - adam.nunn@doctors.org.uk

*Fluids and Barriers of the CNS* 2019, **16(Suppl 3):**O59

**Introduction:** Extended lumbar drainage (ELD) is widely regarded as the most accurate test for shunt-responsive idiopathic normal pressure hydrocephalus (iNPH), however, recent studies have questioned the negative predictive value of this test, raising the possibility that diagnostic algorithms relying upon it may inappropriately exclude patients from treatment. To determine the accuracy of ELD as a diagnostic test for shunt-responsive (or ‘definite’) iNPH among patients referred to secondary/tertiary care with suspected iNPH, meta-analysis of appropriately rigorous studies was undertaken.

**Methods:** Databases were searched on 5th September 2018 using keywords derived from the terms ‘normal pressure hydrocephalus’ and ‘lumbar CSF drainage’. The steps of the PRISMA flow diagram were followed. The QUADAS-2 tool was used to screen studies for methodological concerns. Data on diagnostic test accuracy were extracted and subjected to bivariate random-effects meta-analysis.

**Results:** Only 4 of 273 screened studies met the inclusion criteria. All were small studies (range 7–38 subjects), and all but one had significant concerns over bias. They showed disparate results concerning diagnostic test accuracy. The summary estimates for sensitivity and specificity were 94% (CI 41–100%) and 85% (CI 33–100%), respectively. Assuming a prevalence of 60% among patients referred with suspected iNPH, the summary estimates of NPV and PPV were both 90% (CIs 65–100% and 48–100%, respectively).

**Conclusions:** ELD has a relatively high diagnostic accuracy, however, these estimates are imprecise owing to the quality of existing evidence. A large cohort study, or an RCT comparing diagnostic algorithms with and without ELD is urgently needed to determine best practice.

## O60 Revision of the cerebrospinal fluid dynamics using various types of magnetic resonance imaging

### Ryo Oike^1^, Mitsunori Matsumae^1^, Kagayaki Kuroda^2^

#### ^1^Department of Neurosurgery, School of Medicine, Tokai University, Isehara, Kanagawa, Japan; ^2^Department of Human and Information Sciences, School of Information Science and Technology, Tokai University, Hiratsuka, Kanagawa, Japan

##### **Correspondence:** Ryo Oike - ryo.st2b@gmail.com

*Fluids and Barriers of the CNS* 2019, **16(Suppl 3):**O60

**Introduction:** It has been widely considered that the cerebrospinal fluid (CSF) flows unidirectionally and circulates through the ventricles and subarachnoid space in downward and upward directions, which is called “CSF circulating theory”. A consensus of CSF motion, however, has been changing. We currently understand that CSF motion is not a circulatory flow, but a combination of various directions of flow in the ventricles and subarachnoid space and the acceleration of CSF motion differs depending on the CSF spaces. Classical theory has been based on the results from the quantitative analysis of CSF motion. Currently, water molecule motion in the order of centimeters per second can be detected with various magnetic resonance imaging (MRI) techniques. We made a comparative discussion about this CSF motion through different MRI techniques.

**Methods:** We analyzed CSF motion in the various parts of ventricular systems using different MRI techniques: time-resolved three-dimensional phase contrast (3DPC), time-spatial labeling inversion pulse (time SLIP), dynamic improved motion-sensitized driven-equilibrium steady-state free precession (dynamic iMSDE SSFP), and findings that were shared among these techniques were extracted.

**Results:** We observed various-directional CSF motion and the irregular acceleration, especially using the new MRI techniques with high-velocity sensitivity, such as in the order of 10 μm/s.

**Conclusions:** Every MRI method understandably has advantages and disadvantages based on its unique principle of imaging. However, it would be possible to determine the essence of CSF motion by taking the findings that are mutual between each imaging methods.

## O61 Continuous CSF flow visualisation using SSFP pulse sequence

### Koichi Oshio^1^, Shinya Yamada^2,3^, Masao Yui^4^, Seiko Shimizu^4^

#### ^1^Department of Diagnostic Radiology, Keio University School of Medicine, Tokyo, Japan; ^2^Department of Neurosurgery, Kugayama Hospital, Tokyo, Japan; ^3^Department of Neurosurgery, Juntendo University, Tokyo, Japan; ^4^Canon Medical Systems Corporation, Japan

##### **Correspondence:** Koichi Oshio - oshio@med.keio.ac.jp

*Fluids and Barriers of the CNS* 2019, **16(Suppl 3):**O61

**Introduction:** There are two groups of methods to visualise water motion using MRI, namely, phase contrast methods and spin labelling methods. The phase contrast methods measure each voxel’s average velocity, which may become a significant drawback when measuring flow where large intra-voxel velocity gradient exists, like the aqueduct. In the spin labelling techniques, the observation duration is limited to several seconds due to T1 relaxation. Here we propose a new technique that can visualise relatively slow flow continuously.

**Methods:** The pulse sequence is based on SSFP (steady-state free precession), and positively utilises the dark band artifacts of the SSFP sequence. A relatively large field gradient is introduced along one direction to make equally spaced dark bands appear. When the CSF moves, the dark bands follow the motion of the water.

We developed a modified version of SSFP, where the direction and the interval of the bands are freely controlled by the operator.

A numerical simulation was also performed to investigate the precise relationship between the band motion and the actual flow.

**Results:** CSF flow was clearly visualised with healthy volunteers. The imaging speed was 9 frames/s, and the in-plane spatial resolution was about 2 mm. It was possible to acquire images continuously for 60 s.

**Conclusions:** A new technique was developed to visualise CSF flow continuously.

## O62 CSF protein content estimation by T2 component analysis

### Koichi Oshio^1^, Shinya Yamada ^2,3^, Masao Yui^4^, Seiko Shimizu^4^

#### ^1^Department of Diagnostic Radiology, Keio University School of Medicine, Tokyo, Japan; ^2^Department of Neurosurgery, Kugayama Hospital, Tokyo, Japan; ^3^Department of Neurosurgery, Juntendo University, Tokyo, Japan; ^4^Canon Medical Systems Corporation, Japan

##### **Correspondence:** Koichi Oshio - oshio@med.keio.ac.jp

*Fluids and Barriers of the CNS* 2019, **16(Suppl 3):**O62

**Introduction:** CSF is thought to help large molecules to move in and out of the brain, but exactly how it is done is largely unknown. We tried to acquire some information about large molecule transport by CSF, by closely analysing the T2 of the CSF. We assume that the T2 of the CSF varies with protein content, the higher the content, the shorter the T2.

**Methods:** Multiple spin-echo images were acquired using a CPMG (Curr, Purcell, Meiboom and Gill) imaging sequence. Since each voxel may contain many T2 components, due to either intra-voxel small structures or due to simple partial volume effect, the decaying signal was decomposed into many components using NNLS (non-negative least squares) decomposition.

**Results:** 25 echoes were acquired with echo interval of 40 ms. The resulting echo times for each image were: 40, 80,…, 1000 ms. The decaying signal was decomposed into 25 components, with pixel by pixel basis. Components with 300 ms or longer T2 were considered to be CSF, without partial volume effect. The CSF component had mostly a single T2 group, and average T2 was calculated for each pixel.

Even within the components which purely consists of CSF had a wide range of T2 values, from 500 to 2000 ms, and the spatial variation of the average T2 values was visualised, which is considered to correlate to protein content of the CSF.

**Conclusions:** A method to estimate T2 component of CSF without partial volume effect was developed.

## O63 Clinical significance of vitamin D concentration in idiopathic normal pressure hydrocephalus after shunt surgery

### Ki-Su Park^1^, Kyunghun Kang^2^, Myong-Hun Hahm^3^, Mi Ju Kim^4^

#### ^1^Department of Neurosurgery, School of Medicine, Kyungpook National University, Daegu, 41404, Republic of Korea; ^2^Department of Neurology, School of Medicine, Kyungpook National University, Daegu, 41404, Republic of Korea; ^3^Department of Radiology, School of Medicine, Kyungpook National University, Daegu, 41404, Republic of Korea; ^4^Department of Obstetrics & Gynecology, School of Medicine, Kyungpook National University, Daegu, 41404, Republic of Korea

##### **Correspondence:** Ki-Su Park - kiss798@gmail.com

*Fluids and Barriers of the CNS* 2019, **16(Suppl 3):**O63

**Introduction:** Vitamin D has multiple functions in the central nervous system. Especially, many studies reported that decreased serum 25-hydroxyvitamin D (25OHD) concentrations may be associated with cognitive disorders and larger lateral cerebral ventricles. However, there has been no report about the relationship between the vitamin D concentration and idiopathic normal pressure hydrocephalus (iNPH). The purpose of this study was to investigate the effect of vitamin D concentration on the clinical prognosis in iNPH after surgery.

**Methods:** This research was conducted by Korea Brain Bank Network Project operated through Korea Brain Research Institute funded by the Ministry of Science and ICT. Between 2016 and 2018, 37 patients with iNPH underwent ventriculoperitoneal shunt surgery. Serum 25OHD concentration was quantified at shunt surgery. The patients were categorized into two groups, such as low 25OHD group (< 15 ng/ml) and high 25OHD group (> 15 ng/ml), and analyzed in terms of clinical and radiological findings.

**Results:** Thirty-seven patients consisted of 19 patients with low 25OHD concentration (mean, 73.1 ± 6.4 years; 36.8% female) and 18 patients with high 25OHD concentration (mean, 74.7 ± 4.3 years, 44.4% female). The relationship between the 25OHD concentration and Mini-Mental Status Examination showed the positive correlation with a statistically marginal significance (*r *= 0.299, *p *= 0.06). Additionally, 5 chronic subdural hematomas (CSDH) after shunt surgery were developed, and all of them with CSDH were included in low 25OHD group (*p *= 0.046).

**Conclusions:** Low serum 25OHD concentrations may be associated with cognition impairment before shunt surgery and CSDH after shunt surgery in iNPH.

## O64 Performance analysis of the initial pressure selection protocol for the sphera pro programmable valve

### Fernando C. G. Pinto, Rodolfo C. Reis, Manoel J. Teixeira

#### Department of Neurosurgery, University of São Paulo, 05403-903, Brazil

##### **Correspondence:** Fernando C. G. Pinto - neurofernando@gmail.com

*Fluids and Barriers of the CNS* 2019, **16(Suppl 3):**O64

**Introduction:** The correct choice of initial valve programming at the time of shunt implantation improves the clinical outcome. The objective of this study was to evaluate the efficiency of the initial adjustment protocol for Sphera Pro valve.

**Methods:** Twenty-four patients underwent surgical treatment for implantation of the Sphera Pro valve. The protocol for initial choice of valve programming was elaborated considering the diagnosis of the hydrodynamic disorder: NPH and arachnoid cyst at 3 cmH2O, pseudotumor cerebri at 16.5 cmH2O. The antigravitational device implanted in all patients was 15 cm H2O, except for arachnoid cyst.

**Results:** Twenty NPH patients were included in the study and presented with progressive clinical improvement, with the mean scores on the Japanese scale being 5.7, 3.9, 2.6, 1.3, 1.3 corresponding to the pre, 10 days, 3, 6 and 12 months postoperative periods. Three patients presented with improvement after reduction of the pressure from 3 to 1 cm H2O after 3 months, one patient presented with overdrainage with formation of subdural collection and reversed with pressure adjustment to 21 cm H2O for 30 days and progressive opening up to 10 cm H2O. Three patients with pseudotumor cerebri were included in the study and improved, one needing to have the valve removed by infection. A patient with arachnoid cyst presented clinical improvement and did not require adjustment.

**Conclusions:** The protocol was efficient in 83.3% of the cases. The valve pressure initially chosen was maintained in 20 of the 24 patients for a period of 1 year, with clinical evidence of progressive improvement.

## O65 Automated pressure and volume controlled CSF drainage for the management of a complex lumbar epidural pseudomeningocoele

### Laura Pradini-Santos, Claudia L. Craven, Joana Ramos, Simon Thompson, Lewis Thorne, Parag P. Sayal, Laurence D. Watkins, Ahmed K. Toma

#### Victor Horsley Department of Neurosurgery, National Hospital for Neurology and Neurosurgery, Queen Square, London, WC1N 3BG, UK

##### **Correspondence:** Laura Pradini-Santos - laurapradini@gmail.com

*Fluids and Barriers of the CNS* 2019, **16(Suppl 3):**O65

**Introduction:** Ehlers-Danlos syndrome (EDS) is known to be associated with cerebrospinal fluid (CSF) disturbances, including recurrent CSF leak and Chiari-I malformations. Persistent pseudomenginocoeles are known to be associated with raised ICP. We present an unusual case of a compressive epidural CSF collection after CT-guided L5 nerve root block and describe the effective management strategy.

**Methods:** Retrospective case report. ICP was monitored using Liquoguard^®^7 (Moller Medical GmbH). ICP data was processed using Excel (Microsoft) and on GraphPad Prism 6.0c.

**Results:** A 29 year old female known for EDS presented with a symptomatic L4-S2 epidural compressive CSF collection 5 weeks after CT-guided L5 nerve root block. Eleven days after decompression and repair, she developed a symptomatic subcutaneous pseudomeningocoele, for which she had a second repair and insertion of a lumbar drain, that was then connected to a Liquoguard^®^7 (Moller Medical), a pressure and volume controlled CSF drain that enables ICP monitoring with a safety mechanism to prevent over-drainage, due to her Chiari-I malformation. Median ICP decreased from 24 mmHg after post-repair to 12 mmHg on day 7, before drain removal. There was no further CSF leak or collection at 3 months.

**Conclusions:** EDS in the presence of raised ICP presents a unique challenge as the propensity for CSF leaks due to frail dura is exacerbated by the raised pressure driving the CSF leak. Digitally pressure and volume controlled lumbar drainage can assist with decisions regarding mobilising and the need for further CSF diversion. Nerve root blocks in EDS patients should be considered with caution.

* *Informed consent to publish has been obtained from the patient*

## O66 Idiopathic intracranial hypertension and ovarian cycle: correlation of oestrogen levels with intracranial pressure and pulse amplitude

### Laura Pradini-Santos, Claudia L. Craven, Simon Thompson, Lewis Thorne, Ahmed K. Toma, Laurence D. Watkins

#### Victor Horsley Department of Neurosurgery, National Hospital for Neurology and Neurosurgery, Queen Square, London, WC1N 3BG, UK

##### **Correspondence:** Laura Pradini-Santos - laurapradini@gmail.com

*Fluids and Barriers of the CNS* 2019, **16(Suppl 3):**O66

**Introduction:** Idiopathic intracranial hypertension (IIH), or pseudotumor cerebri, is a condition predominantly seen in women of childbearing age. Oestrogen is thought to have a role in this condition’s pathogenesis. In this specialised neurosurgical centre, diagnostic workup for IIH involves ambulatory 24 h intracranial pressure (ICP) monitoring. We hypothesise that hormone levels will correlate with cerebrospinal fluid (CSF) dynamics, measured with ICP and pulse amplitude (PA) monitoring.

**Methods:** Prospective analysis of ICP and pulse amplitude assessment. Day of ovulation cycle was recorded to estimate theoretical hormone levels.

**Results:** Five patients underwent continuous 24 h ICP monitoring. In all five patients there was a clear correlation between their ICP, their pulse amplitude and fluctuations in oestrogen according to ovarian cycle, with the patient on day 11 of her cycle (at the time of Oestrogen spike leading to ovulation) showing ICP levels of 20 mmHg and a PA of 11 mmHg, and the patient on day 18 (after Oestrogen dip) showing ICP levels of 5 mmHg and PA of 0 mmHg.

**Conclusions:** These preliminary findings suggests that exploring the relationship between hormonal fluctuations and CSF dynamics, with ICP and pulse amplitude monitoring as well as hormonal level testing on a larger prospective cohort, may aid in better understanding CSF dynamics and open novel avenues of research and eventually therapeutic targets in ICP management.

## O67 The unique challenge of hydrocephalus in achondroplasia

### Harold L. Rekate

#### Department of Neurosurgery, Hofstra Northwell School of Medicine, Hempstead, New York, USA

##### **Correspondence:** Harold L. Rekate - haroldrekate@gmail.com

*Fluids and Barriers of the CNS* 2019, **16(Suppl 3):**O67

**Introduction:** Hydrocephalus in the context of achondroplasia occurs in 15–50% of babies. The management strategies are controversial and complicated. The purpose of this retrospective study is to assess the pathogenesis and provide guidelines for management both at the time of first diagnosis and in follow up of older children and adults struggling with issues of shunt management

**Methods:** This is a retrospective study of children referred for hydrocephalus or treatment of shunt related difficulties seen first between 1985 and 2010.

**Results:** A total of 13 patients carrying both diagnoses of hydrocephalus and achondroplasia were treated. Seven patients were babies seen in infancy for diagnosis and treatment. The other 6 patients had already received ventriculoperitoneal shunts. All of the second group had had recurrent shunt failures (4–82 previous surgeries).

Of 7 children seen for diagnosis without previous treatment, only 1 received and intervention except watchful

The second group was managed with upgraded valves and devices for control of siphoning. These failed in 4 patients who were successfully treated with a second ventricular catheter accessing the subarachnoid space spliced proximal to the valve.

**Conclusions:** The developmental outcome in hydrocephalus in achondroplasia is generally quite good and shunting should be avoided if at all possible. For slit ventricle syndrome common in achondroplasia it is often necessary to include a cisterna magna to ventricle to peritoneal shunt to access both the ventricular CSF and that in the cortical subarachnoid space.

## O68 Long-term improvement of gait and cognition after primary endoscopic third ventriculostomy (ETV) in adult obstructive hydrocephalus

### Nicholas Salterio^1,8^, Thomas J. Zwimpfer^1,8^, Rich Holubkov^2^, Heather Katzen^3,8^, Mark G. Luciano^4,8^, Hailey Jensen^2^, Sean J. Nagel^5,8^, Michael A. Williams^6,8^, Mark G. Hamilton^7,8^

#### ^1^Department of Surgery, University of British Columbia, Vancouver, Canada; ^2^Department of Pediatrics, University of Utah, Salt Lake City, USA; ^3^Department of Neurology, University of Miami, USA; ^4^Department of Neurosurgery, Johns Hopkins University, Baltimore, USA; ^5^Department of Neuro-restoration, Cleveland Clinic, USA; ^6^Departments of Neurology and Neurological Surgery, University of Washington, Seattle, USA; ^7^Department of Clinical Neurosciences, University of Calgary, Canada; ^8^Adult Hydrocephalus Clinical Research Network (AHCRN)

##### **Correspondence:** Thomas J. Zwimpfer - thomas.zwimpfer@ubc.ca

*Fluids and Barriers of the CNS* 2019, **16(Suppl 3):**O68

**Introduction:** In addition to symptoms of raised ICP, adults with obstructive hydrocephalus (OH) often present with cognitive, gait, and/or bladder dysfunction. We previously reported improvement of cognition and gait 3 months following primary adult ETV. This abstract presents long-term results in this group.

**Methods:** OH was identified based on tri-ventriculomegaly on CT and/or MRI. This report focuses on gait velocity (10 m timed gait) and cognitive function (Montreal Cognitive Assessment [MoCA]) at two time points: pre-ETV and ≥ 9 months post-ETV.

**Results:** Sixteen adults underwent primary ETV and completed long-term assessment. Mean age was 60 years and 10 (63%) were male. Etiology: 10 (62.5%) congenital and 6 (37.5%) acquired. Mean long-term follow-up time for cognitive and gait assessments was 14.4 and 13.7 months, respectively. Fifteen of 16 patients completed a long-term MoCA with a median individual change of + 2 points [Q1: + 1; Q3: + 3] (*p* = 0.007). Group medians were 23/30 (pre-ETV) and 26/30 (post-ETV). Twelve of 16 patients completed long-term gait assessments with a median individual change of + 0.4 m/s [Q1: + 0.2; Q3: + .6] in gait velocity (*p* < 0.001). Group medians were 0.7 m/s (pre-ETV) and 1.3 m/s (post-ETV). Improved gait velocity was due to an increase in step rate (cadence), as the number of steps to walk 10 m did not significantly change (*p* = 0.202).

**Conclusions:** ETV in adults with OH results in long-term improvement of cognition and gait velocity when assessed ≥ 9 months post-ETV. Larger cohorts will determine the generalizability of these results. Supported by the Hydrocephalus Association.

## O69 In vitro model of solute transport in the human cerebrospinal fluid system

### Lucas R. Sass, Mohammadreza Khani, Goutham Burla, Elliott Marsden, Gabryel Conley Natividad, Omolola Bangudu, Bryn A. Martin

#### Department of Biological Engineering, University of Idaho, Moscow, 83843, Idaho, USA

##### **Correspondence:** Bryn A. Martin - brynm@uidaho.edu

*Fluids and Barriers of the CNS* 2019, **16(Suppl 3):**O69

**Introduction:** Intrathecal therapeutic approaches for treating diseases of the central nervous system (CNS) often rely on transport in the cerebrospinal fluid (CSF). However, animal models have major physiological limitations with respect to human CSF dynamics. Our group has developed a subject specific in vitro model of the human CSF system. This in vitro model and a computational analogue can provide detailed spatial–temporal distribution of solute concentration over 24 h.

**Methods:** CSF geometry was reconstructed from T2-weighted MRI of a healthy 23-year-old female and included realistic spinal cord nerve roots as well as key intracranial CSF spaces. 3D-printing was used to construct the complete geometry in a transparent material. Time-lapse imaging was used to capture spatial–temporal distribution of fluorescein. Distribution was analyzed under different injection conditions (e.g. injection volume and location, filtration loop, and others) with various CSF flow waveforms (e.g. magnitudes, frequencies). A computational fluid dynamics (CFD) study of the pulsatile in vitro CSF flow field was solved using ANSYS Fluent by computing the steady-streaming velocity field. In vitro results for a specific injection scenario were compared to the CFD simulations by linear regression of the average tracer concentration for 3 mm thick axial slices.

**Results:** Total spinal and intracranial CSF volumes were 100.3 ml and 221.6 ml respectively. Maximum Reynolds number was 461. CFD predicted steady streaming velocities in the cranial subarachnoid space were ~ 50X smaller than in the spine. Agreement of in vitro versus CFD spatial–temporal solute concentration was strong for all injection scenarios analyzed.

## O70 Magnetic resonance imaging quantification of ophthalmic changes due to spaceflight

### Stuart H. Sater^1^, Jesse J. Rohr^1^, Austin M. Sass^1^, Michael B. Stenger^2^, Brandon R. Macias^3^, Doug Ebert^4^, Ashot E. Sargsyan^4^, Karina Marshall Goebel^3^, Alan Hargens^5^, Scott A. Dulchavsky^6^, Robert J. Ploutz-Synder^7^, Bryn A. Martin^1^

#### ^1^Department of Biological Engineering, University of Idaho, Moscow, 83843, Idaho, USA; ^2^Johnson Space Center Cardiovascular and Vision Laboratory, National Aeronautics and Space Administration, Houston, Texas, USA; ^3^Johnson Space Center Cardiovascular and Vision Laboratory, KBR, Houston, Texas, USA; ^4^Johnson Space Center Space and Medical Operations, KBR, Houston, Texas, USA; ^5^University of California San Diego, California, USA; ^6^Henry Ford Hospital, Detroit, Michigan, USA; ^7^Applied Biostatistics Laboratory, University of Michigan, Ann Arbor, Michigan, USA

##### **Correspondence:** Bryn A. Martin - brynm@uidaho.edu

*Fluids and Barriers of the CNS* 2019, **16(Suppl 3):**O70

**Introduction:** Approximately 37% of long-duration spaceflight astronauts develop signs/symptoms of the spaceflight associated neuro-ocular syndrome (SANS), including optic disc edema, chorioretinal folds, ocular globe flattening and hyperopic shifts. Quantification of ophthalmic changes that occur during spaceflight may provide clues into the mechanisms responsible for SANS. Automated and manual methods were developed to quantify optic nerve (ON), optic nerve sheath (ONS), and optic globe geometry to better understand how microgravity may impact these structures.

**Methods:** Magnetic resonance (MR) images were collected from astronauts before and after long-duration spaceflight. 3D ON and ONS geometries were analyzed using threshold-based segmentation to compute cross-sectional area. Threshold segmentation was applied to the optic globe after radially re-slicing MRI sequences. Resulting pre- and post-flight point clouds were aligned using an iterative closest point algorithm. Posterior ocular globe flattening was assessed in terms of volume deformation at a radius of 4 mm around the ONH.

**Results:** No significant changes were observed in ON and ONS geometries after long-duration spaceflight, however some astronauts did exhibit significant flattening of the posterior ocular globe. The average and standard deviation of the posterior globe volume deformation was − 8.3 ± 9.1 mm^3^ (p = 0.0001, N = 20 eyes). Notably, the subject with the greatest degree of posterior ocular globe volume deformation (39.2 mm^3^) was clinically diagnosed grade 1 optic disc edema via fundus imaging. The role of intracranial pressure changes in astronauts presenting with ocular globe deformation in astronauts is unknown.

## O71 Intracranial fluids dynamics alterations and cortical thickness

### Alexandra Vallet^1,2^, Sylvie Lorthois^1^, Nicolas Chauveau^2^, Natalia Del Campo^2^, Laurent Balardy^3^, Patrice Peran^2^, Armelle Lokossou^4^, Olivier Baledent^4^, Pierre Payoux^2^, Eric Schmidt^2,3^

#### ^1^Institut de Mecanique des Fluides de Toulouse UMR 5502, France; ^2^TONIC UMR 1214, INSERM, Toulouse, France; ^3^University Hospital, Toulouse, France; ^4^BIOFLOW EA 7516, Amiens, France

##### **Correspondence:** Eric Schmidt - schmidt.e@chu-toulouse.fr

*Fluids and Barriers of the CNS* 2019, **16(Suppl 3):**O71

**Introduction:** The issue of cortical atrophy is important in normal aging and disease since it is associated with cognitive and physical impairments. Cortical atrophy is potentially a relevant biomarker for the early diagnosis of Alzheimer’s disease (AD). The vascular component is also an integral part of AD and other late-life neurodegenerative diseases. Abnormalities in blood flow appear before accumulation of abnormal proteins in AD. The occlusion of capillaries by neutrophils is significantly higher in AD animal models than control and reduction of those occlusions with an antibody increases both blood flow and cognitive capacities. Vascular alterations lead to hypoperfusion, oxidative stress and inflammation, which in turn lead to damage of neurons, glia and myelin, predominantly in the white mater.

Implication of vascular pathologies for gray matter remains unclear. A recent study showed that altered cerebral hemodyamics in asymptomatic carotid artery stenosis is associated with cortical thinning. However there is no proven link between vascular pathologies and cortical thinning. We propose to explore brain aging with a combined biomechanical and imaging approach in order to assess both fluid dynamics alterations and brain structural modifications.

We hypothesize that there is a link between altered cerebral hemodynamics and loss of cortical thickness during brain aging.

**Methods:** 80 patients suspected of hydrocephalus were prospectively involved. All patients complain of gait alteration, urinary difficulties, mild apathy and ventriculomegaly on brain imaging. They underwent brain MRI with T1 weighted images to quantify cortical thickness and phase contrast images to measure arterial, venous and CSF velocities. Lumbar infusion test was also performed to gauge lumbar pressure, a surrogate marker of intracranial pressure (ICP), and CSF dynamics. The cortical volumetric segmentation was done by an automatic post-processing analysis with FREESURFER. Venous, arterial and CSF velocities were measured from PCMRI with BIOFLOWIMAGE software. ICP and CSF dynamics were extracted from infusion tests. Pearson correlations were calculated between cortical thickness and arterial, venous and CSF velocities, but also ICP and derived indices.

**Results:** Mean cortical thickness was positively correlated with mean ICP (r = 0.48, p = 0.001), ICP pulse amplitude (r = 0.43, p = 0.001), arterial flow (r = 0.44, p = 0.001), aqueductal CSF flow(r = 046, p = 0.001), but negatively correlate with venous flow (r = -0.44, p = 0.001)

**Conclusions:** We demonstrate that cortical thickness is correlated with arterial and CSF pulsatility. The causality is more complex; however the association between intracranial pulsatility and gray matter thickness suggests that there is a relationship between vascular alterations at the macroscale level and the pathobiology of cortical atrophy.

## O72 Ventricular collapse after ventriculo-peritoneal shunting for pseudotumor cerebri: a parallel with pediatric slit-ventricle syndrome

### Riccardo Serra^1^, Giorgia Antonia Simboli^3^, Smruti Mahapatra^2^, Noah Leviton Gorelick^1^, Lacie Manthripragada^1^, Mark Gregory Luciano^1^

#### ^1^Department of Neurosurgery, Johns Hopkins University, Baltimore, Maryland, 21287, USA; ^2^Department of Biomedical Engineering, Johns Hopkins University, Baltimore, Maryland, 21287, USA; ^3^Department of Neurosurgery, Catholic University of the Sacred Heart, Rome, 00100, Italy

##### **Correspondence:** Riccardo Serra - rserra3@jhmi.edu

*Fluids and Barriers of the CNS* 2019, **16(Suppl 3):**O72

**Introduction:** Headaches, collapsed ventricles and slow valve refill are part of the Slit-Ventricle Syndrome (SVS) triad, a serious complication that may follow CSF shunting. Overdrainage and SVS, accurately characterized in pediatric hydrocephalus, are only occasionally reported among adults. With this study we aim to shed light on the clinical presentation, management and treatment outcomes of symptomatic ventricular collapse in a cohort of adults with Pseudotumor Cerebri.

**Methods:** We performed a retrospective IRB-approved analysis of 152 patients followed at the Johns Hopkins Hospital between 2015 and 2019. For each patient, demographic data, type of shunt, number/type of failures/revisions, radiological and symptomatic evidence of collapsed ventricles, treatment, and outcomes were collected. Pre- and post-shunting, post-ventricular collapse and post-treatment images were also reviewed and analyzed.

**Results:** In our retrospective cohort we identified 48 patients presenting with different degrees of ventricular collapse. In particular, 29 patients presented with bilaterally collapsed ventricles, while 19 showed unilateral slit-like appearance. Patients were treated with increased valve resistance in 24 cases, 7 received anti-siphoning devices (ASD), 4 Lumbar-peritoneal shunting (LPS), 13 valve/catheter revision. Twenty-nine patients showed symptom improvement and ventricular re-expansion following treatment (positive outcome). Within those, 13 subjects with shunt adjustment had positive outcomes, with 8 showing temporary/negative responses (no improvement in headaches/ventricular size). On the other hand, LPS, addition of ASD, and valve revision uniformly achieved positive outcomes.

**Conclusions:** With this series we provide evidence that strategies routinely used in pediatric patients (LPS shunting, valve adjustment, and valve replacement) often result in ventricular re-enlargement and symptom resolution in adults with Pseudotumor Cerebri.

## O73 Intracranial pressure in chronic post-traumatic headache and ventriculo-atrial shunt as a possible treatment modality

### Kiyoshi Takagi^1^, AKazuyoshi Kato^2^, Kenji Onouchi^3^

#### ^1^NPH Center, Nagareyama Central Hospital, Nagareyama, Chiba, 2700114, Japan; ^2^Abiko Seijinkai Hospital, Abiko, Chiba, 2701177, Japan; ^3^CTsukuba Hospital, Tsukuba, Ibaraki, 3050043, Canada

##### **Correspondence:** Kiyoshi Takagi - paulktkg@mac.com

*Fluids and Barriers of the CNS* 2019, **16(Suppl 3):**O73

**Introduction:** Chronic post-traumatic headache (CPHT) and/or mild traumatic brain injury (mTBI) are serious sequela of head injury (JAMA 300; 711-9, 2008, J Neurotrauma 34; 1524-30, 2017). Post-traumatic headache in whiplash-associated disorder was suggested to have orthostatic nature similar to that of spontaneous intracranial hypotension (Anesth Analg 105; 809-14, 2007). We investigated the intracranial pressure (ICP) in CPTH and studied the effect of cerebrospinal fluid (CSF) removal in these patients.

**Methods:** ICP was measured by lumbar puncture in consecutive 279 CPTH patients. CSF was removed unless the patient did not complaint headache. Data were shown in mean (SD), Student’s t-test was used and the statistic significant level was set p < 0.03.

**Results:** Mean age was 39.3 (14.1) y. Mean ICP was 149.8 (46.4) mmH2O and the male ICP was significantly higher than the female ICP (162.7 (45.4) mmH2O vs 138.0 (44.7) mmH2O, p < 0.0001). CSF removal did not cause serious adversary events. In 139 patients, their symptoms such as headache and sore eye were temporally reduced. In the 13 patients with repeated CSF removal, ventriculoatrial (VA) shunts were installed and 6 patients needed no further treatment. Other 7 patients also showed reduced symptoms.

**Conclusions:** Despite the orthostatic nature of headache in CPTH, ICP was not low but even high in many patients. CSF removal was effect at least in about 50% of the patients. VA shunt can be a therapeutic modality for CPTH.

## O74 Ventricular catheter misplacement in ventriculoatrial shunt

### Kiyoshi Takagi^1,2^, Ryuzaburo Kanazawa^2^, Takanori Uchida^2^, Tetsuhiro UHigashida^2^, Manabu Osakabe^2^, Yuichi Takahasi^2^

#### ^1^NPH Center, Nagareyama Central Hospital, Chiba, 2700114, Japan; ^2^Nagareyama Central Hospital, Chiba, 2700114, Japan

##### **Correspondence:** Kiyoshi Takagi - paulktkg@mac.com

*Fluids and Barriers of the CNS* 2019, **16(Suppl 3):**O74

**Introduction:** Ventricular shunt insertion is most frequently used to treat normal pressure hydrocephalus (NPH). Although for the dilated ventricular system, ventricular catheter (VC) misplacement is not rare, that is the commonest reason for the early shunt revision. The purpose of this study is to investigate the rate of VC misplacement and shunt revision due to the misplacement in our series.

**Methods:** We operated on 685 consecutive NPH patients by ventriculoatrial (VA) shunt using programmable valve. Right occipital insertion was performed in all cases. Preoperative CT scan was performed to determine the site of bur hole opening and the direction of VC insertion. In the operation room, the insertion direction was determined based on the preoperative CT scan and the target was marked by using laser pointer. Cerebrospinal fluid (CSF) outflow was confirmed just before atrial catheter insertion. Postoperative CT scan was performed just after the surgery to detect the position of the ventricular catheter. When the misplacement was observed and the symptoms of NPH were not improved after reducing the pressure setting, shunt revision was performed.

**Results:** VC misplacement was observed in 11 cases (1.6%). Although the catheter position was not correct, clinical symptoms were improved in 4 cases. Therefore, only 7 cases (1.0%) required early shunt revision because of the misplacement.

**Conclusions:** The rate of VC misplacement has been reported in 36–60% in the treatment of hydrocephalus. The results of this study emphasize the importance of preoperative marking and intraoperative decision of VC insertion direction.

## O75 Risk factors for PHH among extremely premature infants with severe IVH: a PENUT ancillary study

### Hannah M. Tully^1,5^, Kerry Hancuch^2^, Christopher Traudt^3,4^, Bryan Comstock^2^, Daniel Doherty^4,5^, Sandra Juul^3,5^

#### ^1^Division of Pediatric Neurology, University of Washington/Seattle Children’s Hospital, Seattle, 98105, USA; ^2^Department of Biomedical Statistics, University of Washington, Seattle, 98105, USA; ^3^Division of Neonatology, University of Washington, Seattle, 98105, USA; ^4^Department of Pediatrics, University of Washington/Seattle Children’s Hospital, 98105, USA; ^5^Center for Integrative Brain Research, Seattle Children’s Research Institute, WA 98105, USA

##### **Correspondence:** Hannah M. Tully - hmtully@uw.edu

*Fluids and Barriers of the CNS* 2019, **16(Suppl 3):**O75

**Introduction:** Among premature infants, post-hemorrhagic hydrocephalus (PHH) is a common consequence of severe intraventricular hemorrhage (IVH), yet little is known about why only some infants with severe IVH develop PHH. We sought to assess intrinsic and potentially modifiable risk factors associated with risk of PHH among a cohort of infants with severe IVH.

**Methods:** Retrospective cohort study using data prospectively collected part-of the multi-site PENUT clinical trial from infants born at 24–27 weeks’ gestational age. Demographic and perinatal clinical variables were analyzed by logistic regression to detect factors associated with the subsequent development of PHH. We performed a qualitative and quantitative review of head ultrasounds (HUS) collected at standardized intervals

**Results:** Among the 940 premature infants participating in the PENUT study, 131 experienced severe IVH, 33 (26%) of whom subsequently developed PHH. Compared to infants whose ventricular dilatation resolved, those who developed PHH were no more likely to be male (OR: 1.1, 95% CI: 0.4, 2.8), born by C-section (OR 0.99, 95% CI: 0.4, 2.5), or to have received steroids prior to delivery (OR: 2.5, 95% CI: 0.5, 11.5). Among hemorrhage characteristics, PHH was only seen in infants with bilateral hemorrhage. Periventricular infarction was associated with a threefold higher risk of PHH (RR: 3.1, 96 = 5% CI: 1.8, 5.5). There was no difference in baseline ventricle size on HUS, but by 1 week, lateral ventricles were 16% larger in infants who subsequently developed PHH. Ballooning of the 4th ventricle was seen exclusively in infants who developed PHH, but was a relatively late finding.

**Conclusions:** PHH is observed in one quarter of extremely premature infants with severe PHH. We did not identify easily modifiable clinical factors associated with increased risk, but certain imaging characteristics correlated with subsequent development of PHH. The role of periventricular hemorrhagic infarction, which is visible on early scans and triples the risk of PHH, deserves further exploration.

## O76 Assessment by caregivers in patients with normal pressure hydrocephalus subjected to CSF tap test: an observational-comparative study

### Francesco Tuniz^1^, Renzo Moreale^1^, Maria Caterina Vescovi^1^, Palazzese Paola^1^, Enrico Belgrado^2^, Miran Skrap^1^

#### ^1^Department of Neurosurgery, ASUI S.M. della Misericordia, Udine, Italy; ^2^Department of Neurology, ASUI S.M. della Misericordia, Udine, Italy

##### **Correspondence:** Francesco Tuniz - francesco.tuniz@asuiud.sanita.fvg.it

*Fluids and Barriers of the CNS* 2019, **16(Suppl 3):**O76

**Introduction:** Normal pressure hydrocephalus (iNPH) is a treatable neurological disease. A cerebrospinal fluid (CSF) extraction (tap) test is performed to confirm the diagnosis.

**Methods:** The study aim was to investigate which variables affect the assessment of patient symptoms by caregivers. A questionnaire was developed using the domains of the main scales (Mini Mental State Examination, Tinetti, Barthel) investigating cognition, gait and urinary incontinence. Caregivers were required to complete a questionnaire before and after the CSF extraction test. Furthermore, after the test the caregivers indicated the improvement through a numerical rating scale (NRS). The data were compared with the neurologist and neurosurgeon’s assessments.

**Results:** 25 patients were involved. Average age was 74.42. The patient’s age is correlated with a score in the pre (ß − .480; *p *= 0.018) and post (ß − .539; *p *= 0.007) questionnaire. There was a general improvement in symptoms, with an increase in the final questionnaire score (+ 1.38; range: 18.79 to 20.17 out of the 23 total). This improvement was indicated by caregivers using NRS (0–10) with an average of 4.17 points (0–7). Furthermore, difference between pre and post questionnaire score correlated with pre-test score (ß − .567; *p *= 0.004) and the improvement reported by caregivers through NRS scoring (ß .589; *p *= 0.002). The surgical candidates showed a higher NRS score (5.46 vs 2.20; *p *= 0.002).

**Conclusions:** Caregivers in the assessment of symptoms are influenced by severity of patients’ symptoms in the pre-test. From the data it emerges how the judgment of caregivers through NRS could be a useful tool in the ventriculoperitoneal derivation decision process.

## O77 Resting-state-functional MRI (RS-FMRI) in patients affected by iNPH: changes in default mode network (DMN) and motor network after tap test and surgery. a tool to improve patient selection and outcome

### Francesco Tuniz^1^, Marta Maieron^3^, Daniele Bagatto^2^, Maria Caterina Vescovi^1^, Daniele Piccolo^1^, Renzo Moreale^1^, Maria Cristina De Colle^2^, Enrico Belgrado^4^, Miran Skrap^1^

#### ^1^Department of Neurosurgery, ASUI S.M. della Misericordia, Udine, Italy; ^2^Department of Neuroradiology, ASUI S.M. della Misericordia, Udine, Italy; ^3^Department of Physics, ASUI S.M. della Misericordia, Udine, Italy; ^4^Department of Neurology, ASUI S.M. della Misericordia, Udine, Italy

##### **Correspondence:** Francesco Tuniz - francesco.tuniz@asuiud.sanita.fvg.it

*Fluids and Barriers of the CNS* 2019, **16(Suppl 3):**O77

**Introduction:** Resting-state-functional MRI has drawn attention as a tool to help with a clinical diagnosis and the evaluation of several neuropsychiatric diseases. The aim of this prospective study is to understand if rsMRI could improve the selection of patients for shunt surgery and improve the outcome in the follow-up.

**Methods:** A total of 35 consecutive patients with diagnosis of probable iNPH were submitted to a diagnostic MR examination before and immediately after a tap test and 3 months after surgery. 25 subjects were positive to lumbar infusion test (Group1) while 10 patients were negative (Group2). All the MR-examinations included a T1w-mprage and rsMRI SS-EPI (*200 vol*). Functional data were processed by FSL using MELODIC-ICA and analysis was performed with GLM by dual-regression, p < 0.05. Differences in rsMRI data were assessed within and between Group1 and 2 and in a cohort of healthy controls (HC).

**Results:** DMN z-values 16.99 HC, 13.42_Group1, 10.06_Group2 at baseline. MN z-values 12.92 in HC, 12.31_Group1_7.56 Group2 at baseline.

After invasive tests DMN has a z-values of 14.56_Group1 and 10.6_Group2, MN has a z-values of 14.57 Group1 and 7.23 _Group2. In Group1 we found a significant positive difference from pre to post tap-test and surgery for motor network (p < 0.04), and DMN (p < 0.02). The analysis performed within group 2 pre and post tap-test do not show any improvement.

**Conclusions:** Our data demonstrated that DMN and MN connectivity in patients with iNPH compared with healthy controls are less represented. After tap test and after surgery there is a strong improvement in DMN and MN connectivity. rsMRI could be a promising method to be considered for the selection of iNPH patients for shunt surgery and follow the patient after surgery.

## O78 Transorbital ultrasound with epidural pressure measurements during epidural blood patch in patients with spontaneous intracranial hypotension

### Enrico Belgrado^1^, Francesco Tuniz^2^, Simone Lorenzut^1^, Daniela Cargnelutti^1^, Caterina Vescovi^2^, Miran Skrapp^2^

#### ^1^Department of Neurology, Azienda Ospedaliero-Universitaria, Santa Maria della Misericordia Udine, Italy; ^2^Department of Neurosurgery, Azienda Ospedaliero-Universitaria, Santa Maria della Misericordia Udine, Italy

##### **Correspondence:** Enrico Belgrado - enrico.belgrado@asuiud.sanita.fvg.it

*Fluids and Barriers of the CNS* 2019, **16(Suppl 3):**O78

**Introduction:** Optic nerve sheath diameter (ONSD) can be used to estimate intracranial pressure in a non-invasive way. The aim of our study was to evaluate the changing of ONSD during epidural blood patch (EBP) in spontaneous intracranial hypotension (SIH) as a tool to guide the effectiveness of the induced raise in intracranial pressure. We also measured the corresponding epidural pressure during the infusions.

**Methods:** We enrolled consecutive patients clinically affected by SIH who failed conservative management. We performed an EBP with continuous ultrasound measurement of ONSD with a 7.5 MHz linear probe, in a semi-fixed dose step injection method (5 ml of blood each time). We injected blood according to real-time OSND enlargement. We also measured epidural pressures during the injections in all the patients with LiquoGuard 7^®^ device.

**Results:** We studied 7 patients with SIH. During blood patch test we obtained a significant expansion of ONSD of 0.875 mm (range 0.7–1 mm) in all patients (pre-treatment value 5.4 mm; post treatment value 6.3 mm). All patients except one showed resolution of symptoms; 3/3 patients had the subdural hematomas completely reabsorbed in 4–6 months. In the only one patient who failed, epidural pressures were low and did not change during the procedure.

**Conclusions:** Continuous ultrasound measurement of ONSD during EBP can became an important instrument to guide the correct execution of blood patch in SIH according to a pressure injection rationale instead of a fixed volume-based method.

## O79 Life-line of 17 patients with schizophrenia and idiopathic normal pressure hydrocephalus

### Vasco Vanhala^1^, Tuomas Rauramaa^2^, Ville E. Korhonen^1^, Mitja I. Kurki^1,3^, Mikko Hiltunen^4,5^, Anne M. Koivisto^6^, Anne Remes^4,7^, Hilkka Soininen^4^, Soili M. Lehto^8,9^, Anna Sutela^10^, Ritva Vanninen^10^, Heimo Viinamäki^11^, Juha E. Jääskeläinen^1^, Ville Leinonen^1^, Antti Junkkari^1^

#### ^1^Neurosurgery of Neurocenter, Kuopio University Hospital (KUH) and University of Eastern Finland (UEF), Finland; ^2^Department of Pathology, Kuopio University Hospital (KUH) and University of Eastern Finland (UEF), Finland; ^3^Analytical and Translational Genetics Unit, Department of Medicine, Massachusetts General Hospital, USA, Program in Medical and Population Genetics, Broad Institute of MIT and Harvard, USA, Stanley Center for Psychiatric Research, Broad Institute for Harvard and MIT, USA; ^4^Department of Neurology, Kuopio University Hospital (KUH) and University of Eastern Finland (UEF), Finland; ^5^Institute of Biomedicine, University of Eastern Finland (UEF), Kuopio, Finland; ^6^Neurology of Neurocenter, Kuopio University Hospital (KUH) and University of Eastern Finland (UEF), Finland; ^7^Department of Neurology, University of Oulu, Finland; ^8^Psychiatry and Clinical Research Centre, University of Eastern Finland (UEF), Kuopio, Finland; ^9^Department of Psychology and Logopedics, University of Helsinki, Finland; ^10^Department of Radiology Kuopio University Hospital (KUH) and University of Eastern Finland (UEF), Finland; ^11^Department of Psychiatry, Kuopio University Hospital (KUH) and University of Eastern Finland (UEF), Finland

##### **Correspondence:** Vasco Vanhala - vascov@student.uef.fi

*Fluids and Barriers of the CNS* 2019, **16(Suppl 3):**O79

**Introduction:** Schizophrenia (SCZ) seems to occur three times more frequently among iNPH patients compared to the general aged population in Finland. Enlargement of lateral ventricles has been described in a subpopulation of persons with schizophrenia. Our aim is to further describe the temporal relationship between these two chronic conditions and their radiological findings.

**Methods:** All medical records of the 17 iNPH patients with comorbid SCZ out of altogether 521 iNPH patients, were retrospectively analyzed and lifelines for each person were drawn accordingly. We also systemically searched for computed tomography (CT) or magnetic resonance imaging (MRI) images for the patients that were performed at least a year before the diagnosis of iNPH. Images were re-analyzed by neuroradiologist. Histopathological findings from the cortical brain biopsies were incorporated to the lifeline analysis.

**Results:** SCZ patients seem to have asymptomatic ventricular enlargement in CT several years before clinical diagnosis of iNPH. Onset age for iNPH seems to be significantly lower for SCZ patients.

**Conclusions:** Due to the younger age, iNPH among persons with SCZ might be considered as a unique or secondary form of the NPH-syndrome. Further study is motivated to evaluate the potential common pathophysiological mechanisms of SCZ and iNPH.

## O80 Treatment effect on disorders of CSF dynamics: do changes in ICP correlate with radiology?

### Hasan Asif^1^, Anna Vassiliou^2^, Claudia Craven^1^, Lewis Thorne^1^, Laurence D. Watkins^1^, Ahmed K. Toma^1^

#### ^1^Victor Horsley Department of Neurosurgery, National Hospital for Neurology and Neurosurgery, London, WC1N 3BG, UK; ^2^Bart’s and the London School of Medicine, Queen Mary University of London, E1 2AT, UK

##### **Correspondence:** Hasan Asif - hasan.asif@live.co.uk

*Fluids and Barriers of the CNS* 2019, **16(Suppl 3):**O80

**Introduction:** Disorders of CSF dynamics demonstrate characteristic imaging findings involving the sella turcica and optic nerves. In our centre these patients undergo ICP monitoring. We aim to assess the ICP predictability of these features before and after treatment.

**Methods:** MR imaging for sella volume, optic nerve vertical tortuosity and sheath distension scores were reviewed against respective ICP monitoring data, before and after CSF diversion. Imaging data was blindly collected, with triple reviews for discordance.

**Results:** Four-hundred and thirty-two patients (128M:304F) with suspected or established disorders of CSF dynamics underwent ICPM with recent MR imaging of which 201 had CSF diversion and 231 had not.

Mean ICP of sella morphologies (full/flat/concave/empty) were 0.50, 4.62, 7.53 and 10.1 mmHg respectively in the primary group vs 0.47, 5.45, 7.59 and 11.8 mmHg in the secondary group (ns). AUROC for predicting ICP before treatment was 0.83 and 0.86 after treatment.

Mean ICP of vertical tortuosity scores (none/uni/bilateral) were 3.75, 7.54 and 7.86 mmHg respectively in the primary group vs 3.20, 9.32 and 11.0 mmHg in the secondary group (ns). AUROC for predicting ICP before treatment was 0.69 and 0.77 after treatment.

Mean ICP of rail tracking scores (none/uni/bilateral) were 3.45, 7.38 and 8.32 mmHg respectively in the primary group vs 3.01, 9.79 and 9.06 mmHg in the secondary group (ns). AUROC for predicting ICP before treatment was 0.74 and 0.77 after treatment.

**Conclusions:** The described radiographic features do not change with intervention and remain reliable markers of ICP despite intervention.

## O81 Normal pressure hydrocephalus in the landscape of gerontology: current state of play

### Hélène Villars^1^, Fati Nourhashemi^1^, Eric Schmidt^2^

#### ^1^Department of Gerontology, University Hospital, Toulouse, France; ^2^Department of Neurosurgery, University Hospital, Toulouse, France

##### **Correspondence:** Fati Nourhashemi - nourhashemi.f@chu-toulouse.fr

*Fluids and Barriers of the CNS* 2019, **16(Suppl 3):**O81

**Introduction:** Among age-related neurological diseases, Normal Pressure Hydrocephalus (NPH) is a chronic condition inducing functional decline in older adults. NHP is reversible, since after shunt insertion, autonomy can be recovered. Our purpose is to characterize the impact of NPH on autonomy in older adults.

**Methods:** We prospectively studied a cohort of 76 older adults in a geriatric day care hospital unit with NPH syndrome (gait disturbance, cognitive impairment, bladder control problems and enlarged ventricles on brain imaging). We performed a comprehensive geriatric assessment (CGA). Depending on resistance to CSF outflow measured by lumbar infusion test and clinical examination, we dichotomized the population into two groups: likely NPH/unlikely NPH. We compared the groups using MannWhitney U and Chi^2^ tests.

**Results:** There is no significant difference between the groups.

**Table:** 

	Unlikely NPH (n = 36)	Likely NPH (n = 40)
Age (years)	79 ± 8.3	75.8 ± 9.4
Male	23 (64%)	22 (54%)
BMI	26.8 ± 5.8	26.8 ± 4.95
MMSE	22 ± 5.6	21 ± 5.7
ADL	4.68 ± 1.48	4.75 ± 1.32
IADL	3.50 ± 3.13	4.40 ± 2.80
Gait speed m s^−1^	0.62 ± 0.31	0.69 ± 0.93

**Conclusions:** Half of the patients suspected of NPH had an altered CSF dynamics. CGA is not an appropriate tool to differentiate the two populations. Hence the NPH burden might be underestimated by geriatricians. We plan to detail our understanding of the NPH in this population, with particular interest in functional decline but also in caregivers’ burden, healthcare system utilization and ultimately costs.

## O82 In idiopathic normal pressure hydrocephalus (iNPH) setting the valve opening pressure at the lumbar puncture opening pressure decreases over-drainage

### Tito Vivas-Buitrago, Johan Heemskerk, Juan Pablo Herrera, Ricardo Domingo, Sanjeet Grewal, Nicholas L. Zalewski, Alfredo Quinones-Hinojosa, Ronald Reimer, Robert E. Wharen, Neil R. Graff-Radford

#### Department of Neurosurgery, Mayo Clinic, Jacksonville, Florida, USA

##### **Correspondence:** Tito Vivas-Buitrago - titovivasbuitrago@gmail.com

*Fluids and Barriers of the CNS* 2019, **16(Suppl 3):**O82

**Introduction:** Mayo Clinic Florida published that in shunting for iNPH over drainage complications occurred frequently when a patient’s lumbar puncture opening pressure (LPOP) was set at a standard valve opening pressure (VOP) of 120 cm-H_2_0. After this publication our clinical practice has been to set the initial VOP at the patient’s LPOP. This study compared the prevalence of over-drainage before and after the change in practice.

**Methods:** This is a retrospective analysis of iNPH patients treated at a single institution by two surgeons from 2004 to 2018. The change in policy to set the VOP at the LPOP was in 2012. Objective over-drainage was defined as the radiological presence of subdural hematoma or hygroma.

**Results:** 181 iNPH shunted patients were identified during this period (n = 97 before and n = 84 after). A delta value was calculated as the differential pressure between LPOP and VOP. Mean delta value in the prior practice was 38.76 cmH_2_O ± 34.30, while for the new practice mean delta was − 1.15 H_2_O ± 23.99, p < 0.001. Hematoma/hygroma was seen in 42 patients (30.2%) with a mean-delta-pressure of 27.02 mmH_2_O ± 38.40, while patients with no over-drainage had a mean-delta-pressure of 14.5 ± 33.95 (p = 0.043; CI 0.4–24.7). No significant differences in clinical recovery was seen between shunted patient before and after the implementation of this policy (p = 0.128). Furthermore, no significant association was found for the delta value and recovery (p = 0.309)

**Conclusions:** This study strongly suggests the value of an initial VOP setting should be set close to the LPOP (low delta) to decrease the risk of over-drainage. This occurs without loss of improvement.

## O83 Complications of elective intracranial pressure monitoring for investigation of chronic hydrocephalus

### Craig R. Vonhoff^1,2^, Thomas Wallis^1^, Matthias Jaeger^1,3^

#### ^1^Department of Neurosurgery, Wollongong Hospital, NSW, Australia; ^2^University of Wollongong, NSW, Australia; ^3^ Southwestern Sydney Clinical School, University of New South Wales, Sydney, Australia

##### **Correspondence:** Craig R. Vonhoff - crvonhoff@gmail.com

*Fluids and Barriers of the CNS* 2019, **16(Suppl 3):**O83

**Introduction:** Continuous monitoring of intracranial pressure (ICP) with computerized analysis of ICP waveform characteristics is a diagnostic tool for the management of chronic hydrocephalus. It can be used alone or in combination with other diagnostic modalities, such as CSF tap test or lumbar drainage. The perceived risks of ICP monitoring may be an impediment to its use in the diagnosis of chronic hydrocephalus and prediction of successful treatment with CSF diversion. The aim of this study was to analyze and describe complications of continuous ICP monitoring for the diagnostic management of chronic hydrocephalus.

**Methods:** A retrospective review of prospectively collected cases was performed. 130 consecutive cases of elective intracranial pressure monitoring for investigation of hydrocephalus were reviewed between May 2010 and May 2018. Complications attributable to the procedure, patient demographics, relevant comorbidities and duration of follow up were recorded

**Results:** The majority of patients had idiopathic NPH (n = 93), other conditions were posttraumatic and post-hemorrhagic hydrocephalus (n = 10), testing of shunt function (n = 7) and aqueductal stenosis (n = 8). We saw seizures attributable to the ICP probe insertion (n = 1), hardware malfunction requiring reoperation (n = 5) and subclinical small intracranial hemorrhage (n = 2). There were no major complications from elective ICP monitoring in this cohort with neurological sequelae. There were no recorded cases of infection, no cases of prolonged seizure disorder, and no lasting morbidity.

**Conclusions:** Risks of elective ICP monitoring for investigation of hydrocephalus are low, and should not preclude clinicians suggesting this method of investigation to patients presenting with possible symptomatic hydrocephalus.

## O84 Elucidation of waste clearance in mouse brains

### Hanbing Xu^1^, Masakazu Miyajima^1,2^, Ikuko Ogino^1^, Chihiro Akiba^1,2^, Madoka Nakajima^1^, Hajime Arai^1^, Nobuhiro Tada^3^

#### ^1^Department of Neurosurgery, Graduate School of Medicine, Juntendo University, Tokyo, Japan; ^2^Department of Neurosurgery, Juntendo Tokyo Koto Geriatric Medical Center, Japan; ^3^Genetic Analysis Model Laboratory, Juntendo University, Tokyo, Japan

##### **Correspondence:** Masakazu Miyajima - mmasaka@juntendo.ac.jp

*Fluids and Barriers of the CNS* 2019, **16(Suppl 3):**O84

**Introduction:** We registered quantitatively fluorescence from mouse brain applying an in vivo imaging system (IVIS) and established a method of quantitative measurement for the clearance of solutes.

**Methods:** Small amounts of fluorescent agents [Genhance™ (1086 g/mol), AngioSense^®^ (70,000 g/mol)] were injected into the caudate nuclei of mice to validate (i) the impact of aging on clearance of solutes from the brain, (ii) the effect of CSF drainage on clearance, by quantifying the observations over time with IVIS. Alexa594 (758 MW) and Alexa488 (45 kD) were injected. Frozen brain sections were prepared 15 min and 1 h later, and were evaluated by confocal microscopy.

**Results:** The comparison of the clearance of Genhance™ between 7-week-old mice and 48-week-old mice showed that 22% of the injected substance was cleared in 8-week-old mice within 4 h. In contrast, for the same period only 7% was cleared in the 48-week-old mice. Next, opening of cisterna magna and draining CSF resulted in approx. 2.5-times faster clearance. AngioSense^®^ was not cleared within 4 h. Observations through the confocal microscope revealed that substances with Alexa594 were excreted mainly through capillaries, while those with Alexa488 either entered the perivascular space or were excreted via the choroid plexus.

**Conclusions:** This study established a method for measuring the clearance of waste products from the brain over time in living animals. The clearance of solutes from the brain stagnated due to aging and enhanced CSF drainage promoted faster clearance of low molecular weight solutes.

## O85 Cerebrospinal fluid dynamics in idiopathic and secondary NPH on 4D flow imaging

### Shigeki Yamada^1,2,3^, Masatsune Ishikawa^1,4^, Hiroki Itou^5^, Jun Masumoto^5^, Makoto Yamaguchi^1^, Kazuo Yamamoto^1^, Marie Oshima^3^, Kazuhiko Nozaki^2^

#### ^1^Department of Neurosurgery, Normal-Pressure Hydrocephalus Center, Rakuwakai Otowa Hospital, Kyoto, Japan; ^2^Department of Neurosurgery, Shiga University of Medical Science, Shiga, Japan; ^3^Interfaculty Initiative in Information Studies/Institute of Industrial Science, The University of Tokyo, Japan; ^4^ Rakuwa Villa Ilios, Kyoto, Japan; ^5^Medical System Research & Development Center, FUJIFILM Corporation, Tokyo, Japan

##### **Correspondence:** Shigeki Yamada - shigekiyamada39@gmail.com

*Fluids and Barriers of the CNS* 2019, **16(Suppl 3):**O85

**Introduction:** To elucidate the mechanisms of iNPH and sNPH, we evaluate CSF movement and wall shear stress in iNPH and sNPH on 4D flow MRI.

**Methods:** Eighty patients with iNPH, 10 with sNPH, and 33 controls underwent 4D flow and 3D T2-weighted imaging on 3-Tesla MRI. The flow vectors (velocity and direction) with synchronized heart beat and wall shear stresses in each region of interest were measured using the 4D flow application on SYNAPSE 3D.

**Results:** The reciprocating CSF movement in the control group was the largest at the foramen magnum and decreased as the distance from the foramen magnum increased. The patients with iNPH had higher mean velocities of reciprocating CSF movements in the ventricles and subarachnoid space than the controls. In sNPH, the mean flow velocities significantly increased in the ventricles, but extensively diminished in the subarachnoid spaces compared with controls. CSF swirling and turbulent flow in the third and fourth ventricles was observed only in sNPH. The high-wall shear stress due to the increase of oscillating CSF motion surrounding the cerebral aqueduct was observed both in iNPH and sNPH.

**Conclusions:** This study performed the first quantitative evaluation of flow volumes and wall shear stresses in the wide CSF spaces from the ventricular system to basal subarachnoid spaces among patients with iNPH and sNPH by using 4D flow MRI. The high-wall shear stress surrounding the cerebral aqueduct might have a potential role in the progressive symptoms, common to patients with iNPH and sNPH.

## O86 Automated pipeline for hydrocephalus diagnosis within medical-image repositories. An artificial intelligence approach

### Fernando Yepes-Calderon^1^, Jeffrey Quezada^1^, J. Gordon McComb^1,2^

#### ^1^Division of Neurosurgery, Children’s Hospital Los Angeles, California, 90027, USA; ^2^Department of Neurological Surgery, University of Southern California, Los Angeles, 90033, USA

##### **Correspondence:** Fernando Yepes-Calderon - fernandoyepesc@gmail.com

*Fluids and Barriers of the CNS* 2019, **16(Suppl 3):**O86

**Introduction:** Since delineation exists between the ventricles and the surrounding parenchyma, one can segment the two using magnetic resonance (MR) or computed tomography (CT) images. Employing artificial intelligence (AI) techniques we propose a method to automatically determine ventricular volume within the Picture Archiving Communications System (PACS) that does not violate confidentiality, perturb the system’s daily operation, is secure, and vendor independent.

**Methods:** A strategy employing voxel-based classification through AI and features derived from positional and intensity constraints was used to determine the lateral and third ventricular volume from MR images. Using the same MR imaging data, 3D printed models were created whose volumes were determined using a precise water displacement technique and compared with the volume as measured by AI. The subjects were five pediatric patients with normal size ventricles and three with hydrocephalus.

**Results:** The correlation between the AI determined ventricular volume and those of the 3D models was between 87 and 94% (Jaccard index of similarity).

**Conclusions:** The accuracy of ventricular volume determination is expected to improve with further AI machine learning. When implemented, accurate ventricular volume can be automatically be made a part of the radiology report. Volume and geometrical features of anything that can be segmented should be able to be quantified.

## O87 Quantifying errors induced by manual segmentation in determining ventricular volume

### Fernando Yepes-Calderon^1^, Jeffrey Quezada^1^, J. Gordon McComb^1,2^

#### ^1^Division of Neurosurgery, Children’s Hospital Los Angeles, California, 90027, USA; ^2^Department of Neurological Surgery, University of Southern California, Los Angeles, 90033, USA

##### **Correspondence:** Fernando Yepes-Calderon - fernandoyepesc@gmail.com

*Fluids and Barriers of the CNS* 2019, **16(Suppl 3):**O87

**Introduction:** In hydrocephalus, it is crucial to estimate the volume of the lateral and third ventricles. This volume is calculated using manual segmentation (Mseg) that is tedious, time-consuming and yields operator dependent results. There exist automatic alternatives to estimate volumes, but their validation is performed based on Mseg. Validating with Mseg is not only a scientific contradiction but may lead to unexpected results due to human errors in delineating boundaries. We intend to know how unstable are human-operator estimations.

**Methods:** Printed 3D Models of the lateral and third ventricles are generated from healthy patients in ages [1, 6, 15, 24, 48, 66, 78, 96, 114] months old and from two hydrocephalus patients. The volume of the models (MV) is measured by an electronic device that detects water displacement with a sensitivity of 0.1 ml/s. Once the volume of each model is known, magnetic resonance phantoms are created and scanned. Then, four trained operators perform Mseg on the images of the phantoms. The Intra and extra operator variability, the influence of image view and the absolute errors respect to the MV were analyzed.

**Results:** Variability among operators was of 31%. Intra-operator variability was of 18%. Volume differences respect to MV was of 33%. Errors associated with the view ascended to 101%. All percentages are mean values.

**Conclusions:** Manual segmentation values for ventricular volume are inaccurate, and thus their results should not be used a solely driven concept for diagnosing or treating hydrocephalus, neither to validate automatic segmentation tools.

## O88 The implantation of adjustable gravitational-valve prosa on the sternum to treat pediatric slit ventricle syndrome

### Changming Zhang, Ruoheng Xuan, Chao Yang, Jinlong Liu

#### Department of Neurosurgery, The First Affiliated Hospital of Sun Yat-sen University, Guangzhou, 510080, China

##### **Correspondence:** Jinlong Liu - 2339186268@qq.com

*Fluids and Barriers of the CNS* 2019, **16(Suppl 3):**O88

**Introduction:** The slit ventricle syndrome (SVS) is a chronic complication in pediatric hydrocephalus patients with shunts, characterized with intermittent headaches, slow refilling of the shunt reservoir and small slit-like ventricles on imaging studies. The management of SVS still remains difficult and challenging. In this study we aim to evaluate the effectiveness of implanting proSA on the sternum to treat pediatric SVS.

**Methods:** Three post-VP-shunt pediatric patients diagnosed SVS were selected. Implanting the adjustable g-valve proSA (FV701T, Aesculap AG, Germany) on the sternum was performed for anti-siphon. Clinical outcome, ventricle expansion based on axial CT scans, fronto-occipital horn ratio (FOHR), gray-to-white matter ratio (GWR) of Hounsfield unit (HU) value were evaluated and recorded during 2 years follow-up.

**Results:** Three pediatric patients (10y/o, 11y/r, 4y/o) received proSA treatment, with proSA pressure setting from primary 25, 20, 25 cmH2O gradually to final 35, 25, 36 cmH2O. Clinical symptoms like headache, dizzies were completely released. The ventricular systems recovered from slit-like to normal-shaped on CT scans. Three pediatric patients’ FOHR increased gradually from 0.114, 0.141, 0.108 to 0.293, 0.402, 0.214 at 2y follow-up, reflecting the decent expansion of lateral ventricle. Three pediatric patients’ GWR of HU value at basal ganglia level increased gradually from 1.348, 1.205, 1.270 to 1.403, 1.511, 1.383, indicating that brain edema was released.

**Conclusions:** The implantation of adjustable g-valve proSA on the sternum is a feasible and effective method for treating post-VP-shunt pediatric SVS.

## O89 Defining molecular function and its associated pathway of ETINPH

### Jun Zhang^1^, Madan Baral^2^, Raymond Otoo^2^, Akhil Padarti^1^, Michael A. Williams^3^, Daniele Rigamonti^4^, Abhay Moghekar^4^

#### ^1^Texas Tech University Health Science Center El Paso, TX 79905, USA; ^2^University of Texas at El Paso, TX 79905, USA; ^3^The Adult and Transitional Hydrocephalus Program at University of Washington Medical Center, Seattle, USA; ^4^The Adult Hydrocephalus Center, Johns Hopkins University School of Medicine, Baltimore, Maryland, USA

##### **Correspondence:** Jun Zhang - jun.zhang2000@gmail.com

*Fluids and Barriers of the CNS* 2019, **16(Suppl 3):**O89

**Summary:** We previously reported a novel heritable form of hydrocephalus termed ETINPH (essential tremor-idiopathic normal pressure hydrocephalus) in a large 5-generation pedigree, mapped this ETINPH locus to chromosome 19q12-13.31, narrowed down ETINPH gene to 25 genes on chr 19 with WES (Whole Exome Sequencing), and eventually further narrowed down to 8 genes with non-synonymous mutations. In this report, we employed NGS (Next-generation sequencing) technologies to define the genomic structure of a new ETINPH patient from an unrelated ETINPH pedigree identified recently. NGS was performed with approximate 100X coverage and 100 bp paired-end reads in Illumina 2000. All reads were mapped to human genome GRCh38 to generate genetic variants files, SNP and INDEL. Newly identified genetic variants (SNPs and INDELs) have been compared with the known 8 candidate mutations in the original family with functional prediction, pathway analysis and interactome. The molecular function of ETINPH and its associated pathway will be discussed.

## O90 To explore the value of tap test and monitoring of intracranial pressure in the treatment of idiopathic normal pressure hydrocephalus

### Yan Zheng

#### Department of Neurosurgery, Renji Hospital, Shanghai Jiaotong University School of Medicine, China

##### **Correspondence:** Yan Zheng - zhengyanrj@126.com

*Fluids and Barriers of the CNS* 2019, **16(Suppl 3):**O90

**Introduction:** To reveal the value of cerebrospinal fluid (CSF) tap test and correlation coefficient between pulse amplitude and ICP (RAP) as surgical indications for idiopathic normal pressure hydrocephalus (iNPH).

**Methods:** Clinical data of 260 patients with suspected idiopathic normal pressure hydrocephalus admitted to Renji Hospital, Shanghai Jiaotong University School of Medicine from January 2013 to December 2017, were retrospectively analyzed. Those who were positive for the Tap Test and negative but met the criteria of RAP index > 0.6 after 12 h continuous cerebrospinal fluid pressure monitoring test, accepted shunt surgery. We evaluated the clinical and predictive value of the Tap test, and the RAP index in the diagnosis and treatment of iNPH.

**Results:** Of the 260 patients, 185 patients accepted shunt operation, 181 patients improved after surgery, and 4 patients were without improvement. The overall surgical rate was 71.2%, and the surgical efficiency was 97.8%. The Tap test was positive in 165 patients, 157 patients accepted shunt surgery. There were 155 cases with improvement of clinical symptoms after shunting and 2 cases without improvement. The Tap test has a positive predictive rate of 98.7% for iNPH shunt surgery. While the Tap test was negative, RAP index > 0.6 was positive in 28 cases, including 26 that improved after shunting and 2 unchanged. The RAP index > 0.6 has a positive predictive rate of 92.9% for iNPH shunt surgery.

**Conclusions:** The RAP index > 0.6 can also be used as an effective supplement of surgical indication for therapeutic effect of shunting in patients with iNPH

## P1 Dolichoectasia in patients with normal pressure hydrocephalus

### Andreas Eleftheriou^1^, Fredrik Lundin^2^

#### ^1^Department of Neurology, Linköping University, Sweden; ^2^Department of Clinical and Experimental Medicine, Linköping University, Sweden

##### **Correspondence:** Andreas Eleftheriou - andelef2002@yahoo.gr

*Fluids and Barriers of the CNS* 2019, **16(Suppl 3):**P1

**Introduction:** Vertebrobasilar dolichoectasia (VBD) is a condition encountered in the elderly population characterized by marked elongation, dilatation, and tortuosity of the vertebral and basilar arteries. Arteriosclerosis and hypertension are risk factors that are believed to be partly involved in the pathogenesis. Obstructive hydrocephalus has been described as an infrequent condition of VBD, usually caused by compression of the third ventricle. We report two patients with Normal Pressure Hydrocephalus (NPH) and VBD but without an obstruction raising questions about a possible link between these conditions.

**Methods:** Case 1 was a 72-year-old male and the case 2 was a 71-year-old man. Both of them were referred to our outpatient clinic with cognitive impairment, gait disturbance and urinary urgency. Both patients underwent radiological brain investigations with Computer Tomography (CT), CT-angiography and Magnetic Resonance Imaging which were compatible with NPH and VBD, without appearance of any compression of third ventricular outflow. In addition, there were also periventricular microangiopathic changes in the patients. Both of them fulfilled the international criteria for idiopathic normal pressure hydrocephalus and were improved after shunt surgery.

**Results:** VBD is an arterial disease, mostly attributed to arteriosclerosis. In literature, cases of VBD caused by an obstructive hydrocephalus have been described. However, there is some evidence of a disturbed CSF-dynamics in hydrocephalic patients with VBD but, without an obstruction. Those two patients with NPH and VBD raise the question about a potential link and possible overlooked cause of NPH. Further studies, especially on CSF-dynamics, are needed to clarify this.

* Informed consent to publish has been obtained from the patients

## P2 Cortical thinning and its relation to gait function in idiopathic normal pressure hydrocephalus

### Kyunghun Kang^1^, Jaehwan Han^2^, Yong-Hyun Lim^3^, Ho-Won Lee^1^, Uicheul Yoon^4^, Ki-Su Park^5^, Myoung Nam Kim^6^

#### ^1^Department of Neurology, School of Medicine, Kyungpook National University, Daegu, South Korea; ^2^Department of Medical and Biological Engineering, Graduate School, Kyungpook National University, Daegu, South Korea; ^3^Center of Self-Organizing Software-Platform, Kyungpook National University, Daegu, South Korea; ^4^Department of Biomedical Engineering, College of Health and Medical Science, Catholic University of Daegu, Gyeongsan-si, South Korea; ^5^Department of Neurosurgery, School of Medicine, Kyungpook National University, Daegu, South Korea; ^6^Department of Biomedical Engineering, School of Medicine, Kyungpook National University, Daegu, South Korea

##### **Correspondence:** Kyunghun Kang - kangkh@knu.ac.kr

*Fluids and Barriers of the CNS* 2019, **16(Suppl 3):**P2

**Introduction:** When considering the underlying pathophysiological mechanisms involved in idiopathic normal pressure hydrocephalus (INPH), white matter is often the main locus of investigation. However, when an axon in the brain is damaged, degeneration of the neuron can occur proximally (dying back) and Alzheimer’s disease, associated with cortical thinning, is a common pathologic comorbidity with INPH. We investigated differences in cortical thickness between healthy controls and INPH patients who had a positive response to the CSF tap test. We also evaluated relationships between cortical thinning and Gait Status Scale in INPH patients.

**Methods:** Forty-nine patients diagnosed with INPH and 26 healthy controls were imaged with MRI, including 3-dimensional volumetric images for cortical thickness analysis across the entire brain.

**Results:** INPH patients, when compared to control subjects, showed statistically significant cortical thinning in the left superior frontal gyrus (orbital part), left superior frontal gyrus (medial orbital part), bilateral gyrus rectus, right insula, bilateral parahippocampal gyrus, left fusiform gyrus, right heschl gyrus, right superior temporal gyrus, bilateral temporal pole of the superior temporal gyrus, bilateral middle temporal gyrus, bilateral temporal pole of the middle temporal gyrus, and bilateral inferior temporal gyrus after FDR correction (p < 0.05). Cortical thinning of the right superior frontal gyrus (medial orbital part), right gyrus rectus, right insula, right temporal pole of the superior temporal gyrus, and right superior temporal gyrus was correlated with worse performance in the Gait Status Scale (p < 0.01 uncorrected).

**Conclusions:** Our results indicate that INPH might be a disease exhibiting a characteristic pattern of cortical thinning.

## P3 Lumbo-peritoneal shunt surgery with initial valve setting “virtual off mode” for iNPH patient

### Takashi Kawahara^1^, Masamichi Atsuchi^1^, Takuichiro Higashi^2^, Ryuji Awa^2^, Takuya Iwasaki^1^, Koji Yoshimoto^2^

#### ^1^Division of Neurosurgery, Atsuchi Neurosurgical Hospital, Kagoshima, Japan; ^2^Department of Neurosurgery, Graduate School of Medical and Dental Sciences, Kagoshima University, Japan

##### **Correspondence:** Takashi Kawahara - takashi.kawahara@jifukai.jp

*Fluids and Barriers of the CNS* 2019, **16(Suppl 3):**P3

**Introduction:** Over drainage after lumboperitoneal shunt (LPS) surgery might cause intracranial hypotension. Sometimes, it would induce severe subdural hematoma. To prevent of this complication, controllable valves are available. However, some patients suffer these complications in the condition of highest valve setting.

We report our experience of “Virtual off mode” of the Codman CERTAS Plus Valve for initial valve setting in the LPS surgery for idiopathic normal pressure hydrocephalus (iNPH) patients. We describe the usefulness of highest valve setting of controllable valve for initial valve setting of LPS.

**Methods:** A single-center retrospective study of iNPH patients undergoing LPS procedure with initial valve setting 8 of CERTAS Plus valve from December 2018 to April 2019. Patients’ records were retrospectively reviewed for clinical and subjective outcomes.

**Results:** Continuous 21 iNPH patients underwent LPS surgery with initial valve setting 8 of CERTAS Plus valve. A month after LPS surgery, nineteen cases (90.5%) presented good outcome for NPH symptoms. Seven patients (33%) were not necessarily set lower and setting was kept at 8. There were no severe complications after LPS surgery. For example, postural headache, subdural effusion, chronic subdural hematoma. We compared the outcomes of initial setting of programmable valve seven, we already reported at Hydrocephalus 2019 and the outcomes of setting eight (Virtual off mode). As Virtual off is not completely off, the improvement rate of the symptoms was the same as initial setting 7. And the rate of over drainage is less than initial setting 7. So we think that Virtual off mode is recommended as the initial setting of LPS for iNPH patients.

**Conclusions:** Virtual off mode of Codman CERTAS Plus valve is available for initial valve setting for LPS surgery of iNPH patients. This strategy avoid severe over drainage symptoms and severe subdural hematoma. But some cases were not completely improved, as virtual off setting. So we recommend to adjust the valve setting after 1 week after surgery.

## P4 Hypertension and severe urinary dysfunction are associated with poor outcomes after shunt surgery in idiopathic normal pressure hydrocephalus

### Erena Kobayashi, Shigenori Kanno, Wataru Narita, Kyoko Suzuki

#### Department of Behavioral Neurology and Cognitive Neuroscience, Tohoku University Graduate School of Medicine, Sendai, Japan

##### **Correspondence:** Erena Kobayashi - erena17ren@yahoo.co.jp

*Fluids and Barriers of the CNS* 2019, **16(Suppl 3):**P4

**Introduction:** The aim of the study was to detect the preoperative factors of poor outcomes after shunt surgery in patients with idiopathic normal pressure hydrocephalus (iNPH).

**Methods:** Eighty-eight consecutive patients with iNPH who underwent shunt surgery were enrolled in this study. Shunt responsiveness was defined as an improvement by one or more points on modified Rankin Scale at 1-year follow-up after surgery. To evaluate patients’ symptoms, we administered the Mini-Mental State Examination (MMSE), Frontal Assessment Battery (FAB), the timed up and go test (TUG), and the iNPH grading scale (iNPHGS) before and 1 year after surgery. We also assessed the presence of risk factors for cerebrovascular diseases, including hypertension, diabetes mellitus, hyperlipidemia, and smoking.

**Results:** Thirty-eight patients (43.2%) had good outcomes to surgery (responders), and 50 patients (56.8%) had poor outcomes (non-responders). The prevalence of hypertension in the non-responders was significantly higher than that in the responders. In addition, the median score of iNPHGS urinary dysfunction in the non-responders was significantly higher than that in the responders. Moreover, the logistic regression analysis revealed that the best predictor of poor outcomes was the presence of hypertension (odds ratio = 3.324, 95% CI = 1.334–8.282).

**Conclusions:** There is a possibility that irreversible brain damage in iNPH is facilitated by hypertension. In addition, severe urinary dysfunction in iNPH might be a sign of irreversibility of the brain damage and/or be related to co-morbidity with other neurodegenerative disorders that cause urinary dysfunction.

## P5 Withdrawn

## P6 Corticospinal excitability in iNPH—a transcranial magnetic stimulation study

### Jani Sirkka^1^, Laura Säisänen^2^, Petro Julkunen^2^, Mervi Könönen^2,3^, Elisa Kallioniemi^2,4^, Ville Leinonen^1,5^, Nils Danner^1^

#### ^1^Department of Neurosurgery, Kuopio University Hospital and University of Eastern Finland, Finland; ^2^Department of Clinical Neurophysiology, Kuopio University Hospital, Finland; ^3^Department of Clinical Radiology, Kuopio University Hospital, Finland; ^4^Department of Psychiatry, University of Texas Southwestern Medical Center, USA; ^5^Unit of Clinical Neuroscience, Neurosurgery, University of Oulu, Finland

##### **Correspondence:** Jani Sirkka - janisir@uef.fi

*Fluids and Barriers of the CNS* 2019, **16(Suppl 3):**P6

**Introduction:** Idiopathic normal pressure hydrocephalus (iNPH) is a neurodegenerative disease with an unknown etiology. Lately, disturbed cortical inhibition in motor cortex has been observed in iNPH. Cortical excitability can be evaluated in a noninvasive and painless manner using neuronavigated transcranial magnetic stimulation (nTMS). We characterized for the first time impact of cerebrospinal fluid (CSF) drainage on cortical excitability.

**Methods:** Twenty suspected iNPH patients (16 women and 4 men, mean age 74.4 years ± 4.1 years) with the classical symptom triad and radiological findings were evaluated with nTMS and with motor function tests (10-meter walk test, Grooved Pegboard and Box & Block test). Evaluations were repeated immediately after the CSF drainage via lumbar puncture. From nTMS parameters, we used silent period (SP), motor threshold (MT) and input–output curve (IO-curve) to characterize/assess cortical inhibition, cortical excitability and synaptic plasticity.

**Results:** At the baseline measurement, NPH patients presented shorter SPs (p = 0.002) and lower MTs (p < 0.001) as compared to the healthy, matched control group. Significant positive correlation was detected between SP duration and Box & Block test (r = 0.64, p = 0.002) in iNPH patients. CSF drainage enhanced walking speed (p = 0.003) and IO-slope (p = 0.028).

**Conclusions:** Shorter SPs and lower MTs in iNPH suggest impaired cortical inhibition and cortical hyperexcitability. The steeper IO-slope in patients who improve walking after CSF drainage may indicate a higher potential to benefit from shunt surgery. Cortical excitability correlated with gross motor function of the upper limb implying that the disturbance in motor performance extends beyond the classically reported gait impairment in iNPH.

## P7 Characterization of cardiac- and respiratory-driven csf motions under free breathing using real-time phase contrast technique followed by S-transform and correlation mapping

### Satoshi Yatsushiro^1^, Mitsunori Matsumae^2^, Kagayaki Kuroda^1^

#### ^1^Department of Human and Information Science, School of Information Science and Technology, Tokai University, Hiratsuka, Kanagawa, Japan; ^2^Department of Neurosurgery, School of Medicine, Tokai University, Isehara, Kanagawa, Japan

##### **Correspondence:** Kagayaki Kuroda

*Fluids and Barriers of the CNS* 2019, **16(Suppl 3):**P7

**Introduction:** Understanding of cerebrospinal fluid (CSF) motion consisting of cardiac- and respiratory-driven components, as well as bulk flow is important for clinical diagnosis. To characterize the cardiac- and respiratory-driven CSF motion in the intracranial space under free-breathing, correlation mapping technique in conjunction with Stockwell transform (S-transform) and real-time 2-dimensional phase contrast (2D-PC) imaging was performed for 3 healthy volunteers and 3 patients with hydrocephalus.

**Methods:** Total CSF velocity signal obtained by the 2D-PC technique was separated to the cardiac- and respiratory-driven components by using S-transform (1), a technique to convert a time-varying signal into a spectrogram. The two components were then analyzed by the correlation mapping technique (2) to have delay time map providing propagation delay of CSF motion from a reference point and maximum correlation map representing spatiotemporal similarity of the motion.

**Results:** The maps characterized the cardiac- and respiratory-driven CSF motions. The spatiotemporal correlation of cardiac-driven CSF motion was higher than that of the respiratory in all the healthy subjects. The motion correlations in the patients were lower than those of the healthy.

**Conclusions:** The correlation mapping technique in conjunction with S-transform and real-time 2D-PC imaging was useful for characterizing CSF dynamics in the intracranial space.

## P8 Deep learning for automated tissue segmentation of routine magnetic resonance brain imaging in cerebrospinal fluid disorders

### Mohamad Zeina^1^, Linda D’Antona^2^, Robert Gray^3^, Mikael Brudfors^4^, Suraj Sennik^2^, Sophie Mullins^2^, Laura Pradini Santos^2^, Manjit Matharu^5^, Laurence D Watkins^2^, Ahmed Toma^2^, Parashkev Nachev^3,6^

#### ^1^GKT School of Medical Education, King’s College London, UK; ^2^Victor Horsley Department of Neurosurgery, The National Hospital for Neurology and Neurosurgery, London, UK; ^3^High-Dimensional Neurology Group, University College London Queen Square Institute of Neurology, London, UK; ^4^Wellcome Centre for Human Neuroimaging, University College London Queen Square Institute of Neurology, UK; ^5^Headache and Facial Pain Group, UCL Queen Square Institute of Neurology, UK; ^6^The National Hospital for Neurology and Neurosurgery, University College London Hospital, UK

##### **Correspondence:** Mohamad Zeina - mohamadzeina@gmail.com

*Fluids and Barriers of the CNS* 2019, **16(Suppl 3):**P8

**Introduction:** Modelling brain changes in disorders of cerebrospinal fluid flow requires segmentation of the tissue compartments that abnormal flow tends to disrupt. The morphological abnormalities frequently present in such disorders limit the accuracy and robustness of traditional computational methods. The difficulty arises from the extreme heterogeneity of possible anatomical appearances, which a standard template—the reference for conventional methods—is likely to find great difficulty with. Here we sought to exploit the greater expressive power of deep learning-based methods to create a fully automated algorithm for tissue segmentation that is robust to a wide range of pathological changes, evaluating it on real-world hospital magnetic resonance imaging.

**Methods:** We investigated an unselected set of 301 consecutive patients with cerebrospinal fluid (CSF) flow disorders who had undergone routine magnetic resonance imaging and intracranial pressure monitoring at the National Hospital for Neurology and Neurosurgery. A 3D convolutional artificial neural network architecture was trained and optimized on brains from OASIS-3, and a subset of the clinical scans, to generate probabilistic tissue partitions of grey & white matter, and CSF. Tissue segmentation based on SPM12 was used as a reference conventional approach.

**Results:** We quantified the differences in performance of the two approaches across the dataset, categorized by diagnosis, by visual inspection and comparison with manually defined tissue compartments.

**Conclusions:** Deep learning methods of tissue segmentation are promising, robust alternatives to the traditionally used methods, such as SPM software, which can be brittle when asked to segment anatomically deformed brains.

